# What Singles out Aluminyl Anions? A Comparative Computational Study of the Carbon Dioxide Insertion Reaction in Gold–Aluminyl, −Gallyl, and −Indyl Complexes

**DOI:** 10.1021/acs.inorgchem.1c03579

**Published:** 2022-01-06

**Authors:** Diego Sorbelli, Leonardo Belpassi, Paola Belanzoni

**Affiliations:** † Department of Chemistry, Biology and Biotechnologies, University of Perugia, Via Elce di Sotto, 8, 06123 Perugia, Italy; ‡ CNR Institute of Chemical Science and Technologies “Giulio Natta” (CNR-SCITEC), Via Elce di Sotto, 8, 06123 Perugia, Italy

## Abstract

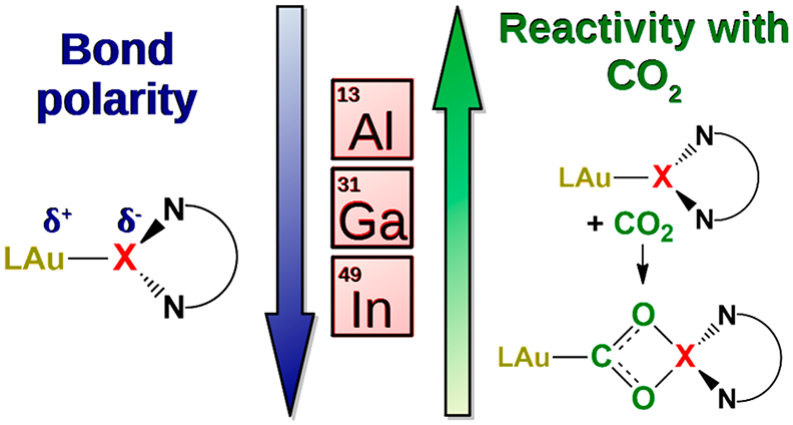

Anionic aluminum(I) anions (“aluminyls”) are the most recent discovery along Group 13 anions, and the understanding of the unconventional reactivity they are able to induce at a coordinated metal site is at an early stage. A striking example is the efficient insertion of carbon dioxide into the Au–Al bond of a gold–aluminyl complex. The reaction occurs via a cooperative mechanism, with the gold–aluminum bond being the actual nucleophile and the Al site also behaving as an electrophile. In the complex, the Au–Al bond has been shown to be mainly of an electron-sharing nature, with the two metal fragments displaying a diradical-like reactivity with CO_2_. In this work, the analogous reactivity with isostructural Au–X complexes (X = Al, Ga, and In) is computationally explored. We demonstrate that a kinetically and thermodynamically favorable reactivity with CO_2_ may only be expected for the gold–aluminyl complex. The Au–Al bond nature, which features the most (nonpolar) electron-sharing character among the Group 13 anions analyzed here, is responsible for its highest efficiency. The radical-like reactivity appears to be a key ingredient to stabilize the CO_2_ insertion product. This investigation elucidates the special role of Al in these hetero-binuclear compounds, providing new insights into the peculiar electronic structure of aluminyls, which may help for the rational control of their unprecedented reactivity toward carbon dioxide.

## Abstract 2

The reactivity of gold−aluminyl, −gallyl, and −indyl complexes with carbon dioxide is predicted to be favorable only for gold−aluminyl complexes. The increased polarity of the Au−Ga and Au−In bonds makes these compounds weaker nucleophiles in a radical-like reaction mechanism.

## Introduction

The problems connected with the increasing concentration of carbon dioxide in the atmosphere^1^ require a continuous effort toward the exploration of efficient and novel solutions for its capture and reduction. Among these, CO_2_ capture with transition metal (TM) complexes is surely one of the most interesting, due to the well-known ability of TMs of activating kinetically and thermodynamically inert CO_2_.^2^ The relative structural simplicity of TM complexes bearing CO_2_ also offers an ideal playground for characterizing in detail the CO_2_ activation mechanisms.

**Scheme 1:**
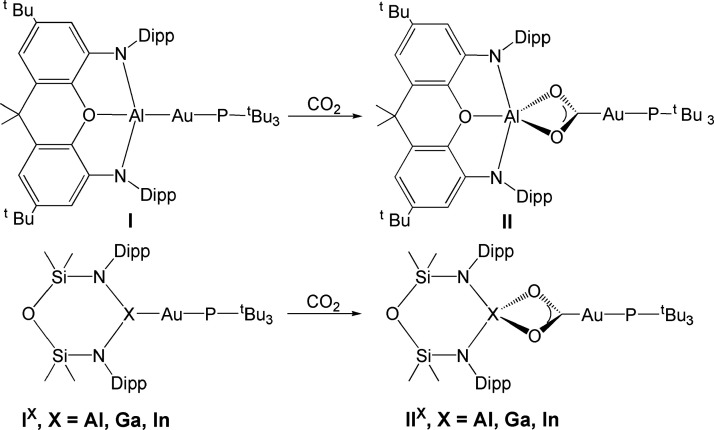
Complexes in This Work Top: Experimentally characterized gold–aluminyl complex (**I**) and the corresponding insertion product upon reaction with CO_2_ (**II**).^3^ Bottom: examples of Group 13 six-membered heterocyclic anions [X(^Si^NON)]^−^ (X = Al, Ga, and In) forming model gold–aluminyl, −gallyl, and −indyl complexes (**I**^**X**^) and their corresponding CO_2_ insertion products (**II**^**X**^).

In this framework, the exceptional reactivity of a molecular gold–aluminyl complex, [^t^Bu_3_PAuAl(NON)] (NON = 4,5-bis(2,6-diisopropylanilido)-2,7-di*tert*-butyl-9,9-dimethylxanthene, complex **I**) was recently reported, in which **I** was easily capable of capturing carbon dioxide at room temperature by inserting it into the Au–Al bond, yielding insertion product **II** (Scheme 1).^3^

Product **II**, with the CO_2_ carbon atom coordinated to Au, was considered to be a probe for a nucleophilic reactivity at the gold site, due to the presence of a supposedly strongly polarized Au^δ−^–Al^δ+^ bond, despite gold complexes being widely known for their electrophilic behavior.^4−7^ This became even more surprising when the CO_2_ insertion into a slightly polarized copper–aluminyl bond was reported, featuring the same CO_2_ insertion mode.^8^

Since a mechanistic description for the formation of **II** was missing, we have recently embarked in a thorough computational exploration of the reaction mechanism and of the nature of the Au–aluminyl bond in **I**.^9^ Our study revealed the bimetallic (Au–Al) activation of CO_2_ with the Au–Al bond being the actual nucleophilic site for the reaction and Al also assisting the activation with the electrophilicity induced by its vacant valence 3p_*z*_ orbital. We showed that the Au–Al bond is of the electron-sharing type, with Au and Al cooperatively inserting CO_2_ with a radical-like reactivity.^9^

In accordance with above reactivity, a zinc–aluminyl complex featuring a highly covalent electron-rich Al–Zn bond was recently found to react with CO_2_ forming an insertion product analogous to **II**.^10^ Following our work, a combined experimental and theoretical paper on the insertion of CO_2_ into Cu–Al, Ag–Al, and Au–Al bonds appeared in the literature, showing a very similar reaction mechanism also for Cu and Ag,^11^ in agreement with the TM–Al bond behaving as a nucleophilic site.

This novel and unexpected reactivity of aluminyls raises questions, mainly about the peculiar chemical nature of the aluminyl and its implications for a rational design of similar bimetallic compounds capable of reacting with carbon dioxide in analogous conditions. While the very recent aluminyls^12^ surely represent promising species for this aim, analogue Group 13 anions (boryls, gallyls) have been known for a longer time:^12^ The first five-membered heterocyclic gallyl anion was reported more than 20 years ago.^13^ Notably, examples of molecular gold–gallyl complexes have been also reported in the past^14,15^ and very recently the silver–gallyl analogue of **I** (i.e., the [^t^Bu_3_PAgGa(NON)] complex) has been characterized.^11^ Formally anionic boryls have also been known for years,^16,17^ and recently, the nucleophilic reactivity of a nonheterocyclic gold–boryl complex toward multiple polar bonds has been reported.^18^ Notably, a copper–boryl complex has been reported in the past to catalyze the reduction of CO_2_ to CO.^19,20^ Concerning the heavier analogue, indium, a six-membered heterocyclic indyl anion (i.e., In(^Ar^NON)]^−^, ^Ar^NON = [O(SiMe_2_NAr)_2_]^2–^, Ar = 2,6-^i^Pr_2_C_6_H_3_) has also been recently reported,^21^ bearing the same heterocyclic backbone of the diamido aluminyl [Al(^Si^NON)]^−^ (^Si^NON = [O(SiMe_2_NDipp)_2_]^2–^, Dipp = 2,6-^i^Pr_2_C_6_H_3_).^22^

The electronic properties of Group 13 heterocyclic anions have been widely investigated theoretically, highlighting that a “gap” separates boryls from their heavier homologues.^23,24^ For instance, heterocyclic boryls are found to have a very small singlet–triplet energy gap,^23,24^ which implies a remarkably low stability, making their experimental isolation very difficult. Indeed, six-membered heterocyclic boryls have not been synthesized yet.^25,26^ However, aluminyl, gallyl, and indyl have a much larger singlet–triplet energy gap,^23,24^ which makes the synthesis of the corresponding heterocyclic anions (and consequently of the reactive TM–X (X = Al, Ga, and In) complexes) much more promising even to explore additional patterns for the bimetallic cooperative TM–X reactivity toward CO_2_.

The aim of this work is to analyze and characterize systematically any analogy/difference between aluminyls and their heavier Group 13 analogues in the framework of the carbon dioxide insertion reaction. We computationally study here the insertion of CO_2_ in the model complexes **I**^**X**^ (Scheme 1). These complexes feature an Al, Ga and In anion with the same heterocyclic structure ([X(^Si^NON)]^−^, X= Al, Ga, and In) combined with a common [^t^Bu_3_PAu]^+^ gold moiety. It is worth reminding that the aluminyl and indyl ligands in complexes **I**^**Al**^ and **I**^**In**^ have been actually synthesized and characterized experimentally^21,22^ and that similar heterocyclic structures appear to be reasonably accessible for Ga. Analogous boryl complex **I**^**B**^ has not been included in this work since the constrained six-member heterocyclic ring structure ([B(^Si^NON)]^−^) represents a poorly realistic model complex, not directly comparable to the aluminyl, gallyl, and indyl analogues. We mention that the experimentally accessible coinage-metal–boryl complexes possess mainly acyclic (such as the very recent gold boryl complex bearing a boryl with two *o*-tolyl substituents)^18^ or five-membered structures.^15,24,27^ A comparative study of the electronic structure of boryl/aluminyl anions, including a systematic analysis of the structural and substituent effects, is currently under way in our laboratory.

By exploring the reaction mechanism for the CO_2_ insertion and carrying out an extensive electronic structure analysis, we will highlight that despite all the complexes (**I**^**Al**^, **I**^**Ga**^, and **I**^**In**^) proceed toward the CO_2_ insertion with the same mechanism observed for complex **I**(9) the reaction is kinetically and thermodynamically significantly disfavored for **I**^**Ga**^ and **I**^**In**^ complexes with respect to **I**^**Al**^. Upon detailed analysis, this is explained by the higher electron-sharing character of the Au–Al bond, which makes it a suitable active site for attacking CO_2_. A radical-like reactivity is shown here to be fundamental for stabilizing the CO_2_ insertion product.

## Results and Discussion

### Reaction Mechanism

For CO_2_ insertion into the Au–Al bond of the [^t^Bu_3_PAuAl(NON)] complex **I**,^3^ we found a two-step mechanism characterized (i) by a nucleophilic attack to the CO_2_ carbon atom performed by the Au–Al bond also assisted by the electrophilic Al “empty” p orbital, followed (ii) by a rearrangement driven by an electrophilic attack to the oxygen atom of CO_2_ by the aluminum center, leading to the formation of the insertion product where the CO_2_ carbon atom is coordinated to gold and both the CO_2_ oxygen atoms are coordinated to Al (complex **II**, see Scheme 1).^9,11^ Transition states and intermediate structures pointed out a radical-like insertion of CO_2_ in the Au–Al bond, which was consistently shown to have mainly an electron-sharing character. In the following, we applied the same systematic computational strategy used in ref (9), that is, density functional theory (DFT) with inclusion of scalar relativistic effects, solvation (toluene), and dispersion corrections (see the “Computational Details” section), for the study of CO_2_ insertion into the Au–X bond in complexes **I**^**X**^ (X = Al, Ga, and In). Analogous to [^t^Bu_3_PAuAl(NON)], complexes **I**^**X**^ have been slightly simplified at the ^Si^NON site by replacing the two Dipp substituents on the nitrogen atoms with phenyl groups (denoted as ^Si^NON′). This modeling has been shown to give good agreement with available experimental geometrical data for complex **I** in ref (9).

**Figure 1:**
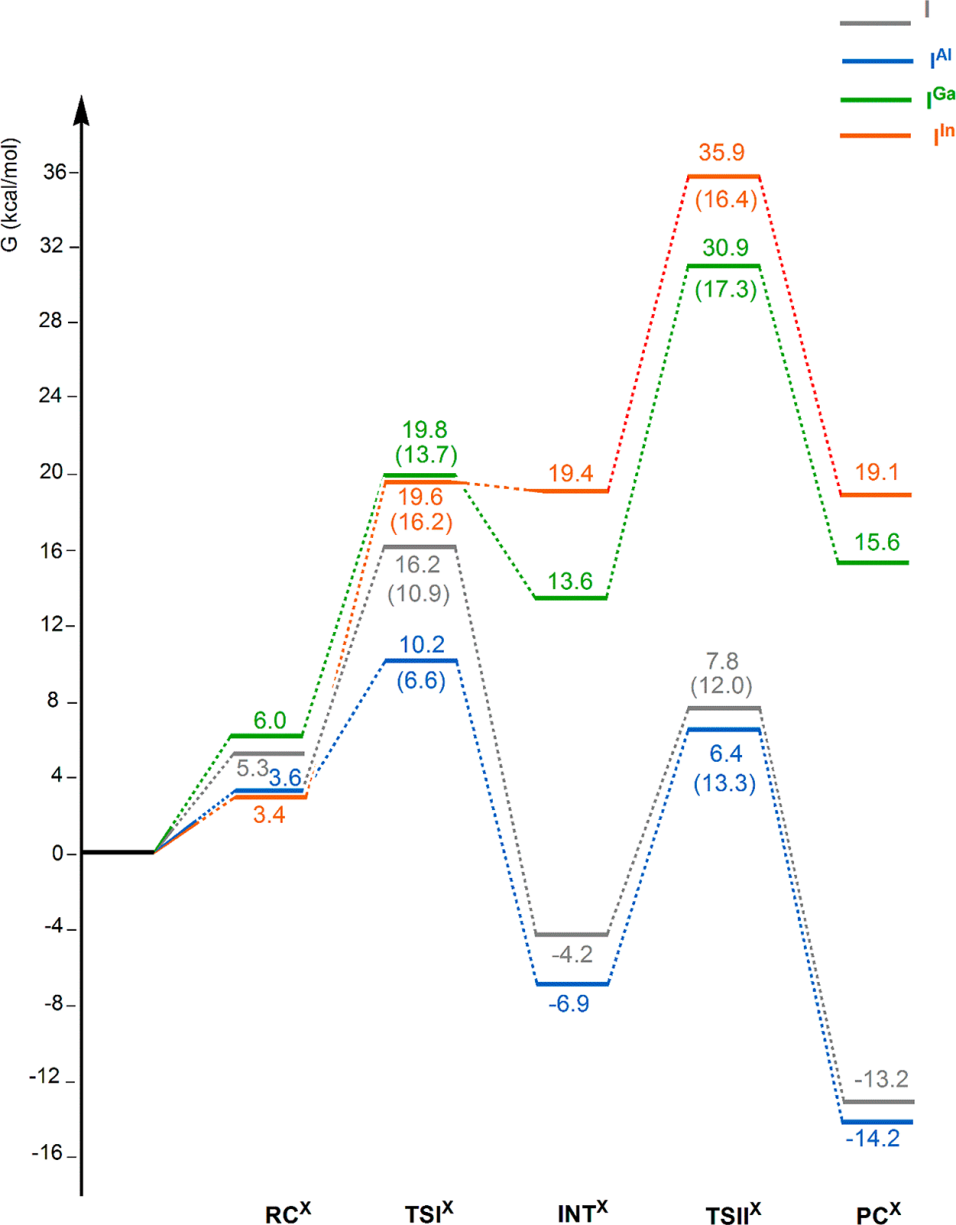
Free energy reaction profile for the CO_2_ insertion into the Au–X (X = Al, Ga, and In) bond in [^t^Bu_3_PAuAl(^Si^NON′)], [^t^Bu_3_PAuGa(^Si^NON′)], and [^t^Bu_3_PAuIn(^Si^NON′)] complexes **I**^**Al**^ (blue lines), **I**^**Ga**^ (green lines), **I**^**In**^ (red lines) and into the Au–Al bond in [^t^Bu_3_PAuAl(NON′)] complex **I** (gray lines) (taken from ref (9)). Δ*G* values refer to the energy of the separated reactants taken as zero. Activation free energy barriers are reported in parentheses. Energy values are in kcal/mol.

**Figure 2:**
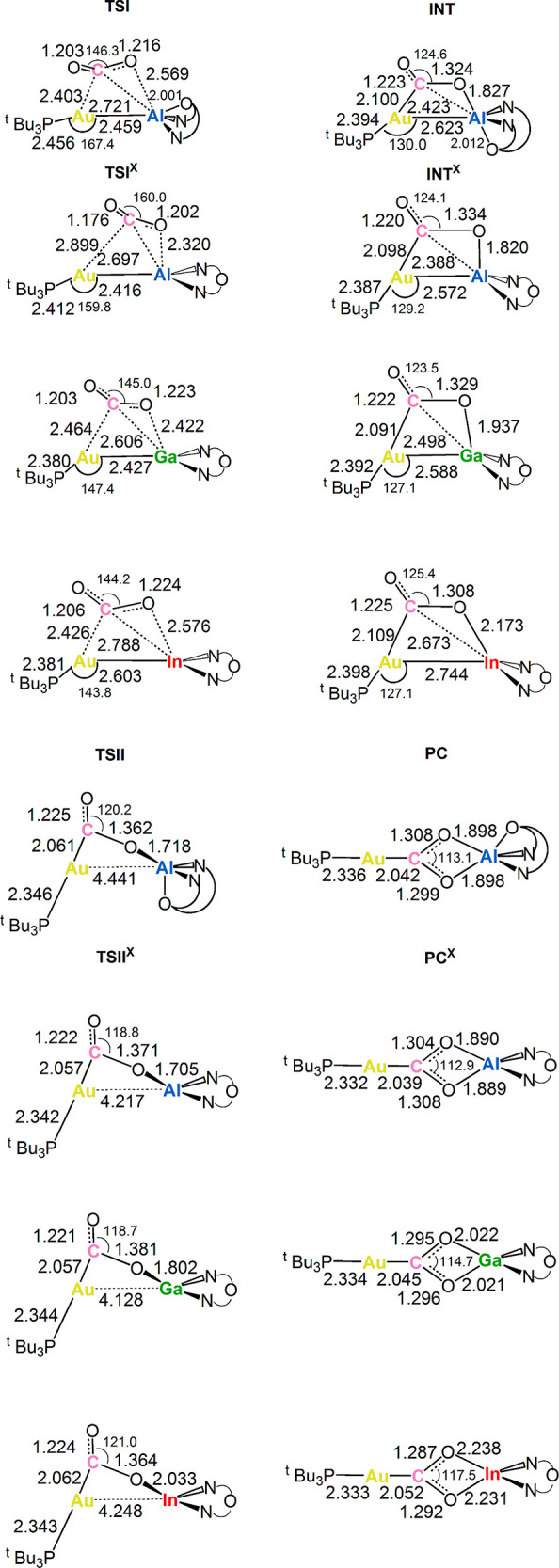
Selected interatomic distances (in Å) and bond angles (degrees) are given with the sketched **TSI**, **TSI**^**X**^, **INT**, **INT**^**X**^, **TSII**, **TSII**^**X**^, and **PC**, **PC**^**X**^ structures of **I** and **I**^**X**^ (X = Al, Ga, and In) complexes.

The free energy profiles for all systems are shown in Figure 1. For the reader’s convenience we also include the reaction profile for the gold–aluminyl [^t^Bu_3_PAuAl(NON′)] complex reported in ref (9). Optimized structures of reactants (RC), transition states (TSI, TSII), intermediates (INT), and products (PC) for [^t^Bu_3_PAuAl(NON′)] and [^t^Bu_3_PAuX(^Si^NON′)] (X = Al, Ga, and In) complexes are sketched with selected geometrical parameters in Figure 2, whereas fully optimized geometries for all the species involved in the whole path are reported in Figures S1–S4. Calculations of the singlet–triplet energy gap in [X(^Si^NON′)]^−^ (X= Al, Ga, and In) anions show that these systems are stable. In particular, it is large for [Al(^Si^NON′)]^−^ (1.47 eV) and even increases from [Ga(^Si^NON′)]^−^ to [In(^Si^NON′)]^−^ (2.14 and 2.23 eV, respectively).^28^

The nucleophilic attack to the CO_2_ carbon atom has a relatively low activation free energy barrier for all the complexes, with the lowest value for complex **I**^**Al**^ (Δ*G*^#^ = 6.6 kcal/mol), the highest for complex **I**^**In**^ (Δ*G*^#^ = 16.2 kcal/mol), and intermediate for complexes **I** and **I**^**Ga**^ (Δ*G*^#^ = 10.9 and 13.7 kcal/mol, respectively) (see Table S1 for the imaginary frequency values of **TSI**^**X**^). With the exception of **TSI**^**Al**^, the transition state structures of the different systems are very similar: The carbon atom of CO_2_ is both close to Au (distances 2.4–2.5 Å) and at a relatively short distance from Al, Ga, and In (2.6–2.4 Å). A substantial bending of CO_2_ and asymmetry between the two C–O bonds are also found. In **TSI**^**Al**^, CO_2_ presents a larger asymmetric coordination, where the Au–C distance increases up to 2.899 Å and the O–Al distance reduces to 2.320 Å. Notably, in this case the CO_2_ distortion is significantly decreased, passing from a bending angle of about 144–146° found for the other systems to 160°. Thus, the structure of **TSI**^**Al**^ is quite unique and apparently difficult to rationalize, both in terms of structure and energy profile along the whole reaction path, particularly in light of its similarity to complex **I**. We will return on this interesting point at the end of this section.

The formation of intermediate **INT**^**X**^ shows substantial differences between systems containing Al and those involving the heavier Group 13 elements. **INT**^**X**^ is stabilized with respect to **TSI**^**X**^ by 20.5 and 17.1 kcal/mol for **I** and **I**^**Al**^, respectively, and by only 6.2 and 0.2 kcal/mol for **I**^**Ga**^ and **I**^**In**^, respectively, thus resulting in an exergonic step for **I** and **I**^**Al**^, an endergonic step for **I**^**Ga**^ and a highly endergonic step for **I**^**In**^. Nonetheless, the **INT**^**X**^ structures share some common features: (i) a slightly increased Au–X bond distance and (ii) an almost linear coordination of the ^t^Bu_3_PAu moiety to the carbon atom of CO_2_. Similar structures are also observed for transition states **TSII**^**X**^ (see Table S1 for imaginary frequency values of **TSII**^**X**^), in which the Au–X bond is significantly elongated and the second oxygen of CO_2_ is approaching X. However, while for **TSII** and **TSII**^**Al**^ similar lower activation barriers are found (12.0 and 13.3 kcal/mol, respectively), **TSII**^**Ga**^ and **TSII**^**In**^ lie at a much higher energy, with corresponding higher Δ*G*^#^ values (17.3 and 16.4 kcal/mol, respectively), suggesting that the **INT**^**X**^ to **PC**^**X**^ conversion would be less favorable for **I**^**Ga**^ and **I**^**In**^. Remarkably, the reverse step from **INT**^**X**^ to **RC**^**X**^ is expected to be kinetically favorable for Ga and, particularly, for In, predicting that the reaction of **I**^**Ga**^ and **I**^**In**^ with CO_2_ to give insertion products **II**^**Ga**^ and **II**^**In**^ is not feasible neither kinetically nor thermodynamically. Product complex **PC**^**X**^ (corresponding to compounds **II** and **II**^**X**^ in Scheme 1) has been calculated to be stable for **I** and **I**^**Al**^ (−13.2 and −14.2 kcal/mol, respectively) and highly unstable for **I**^**Ga**^ and **I**^**In**^ (15.6 and 19.1 kcal/mol, respectively). In our previous study, we investigated the possibility that the product complex **PC** (complex **II** in Scheme 1) may evolve to CO elimination^3^ and we found that the resulting oxide complex [^t^Bu_3_PAuOAl(NON′)][CO] is highly unstable (Δ*G* = 16.6 kcal/mol). Here, the corresponding [^t^Bu_3_PAuOX(^Si^NON′)][CO] (X = Al, Ga, and In) complexes have been also calculated to be unstable with Δ*G* = 14.9, 37.2, and 46.3 kcal/mol, respectively, consistent with the recent results reported in ref (11).

**Figure 3:**
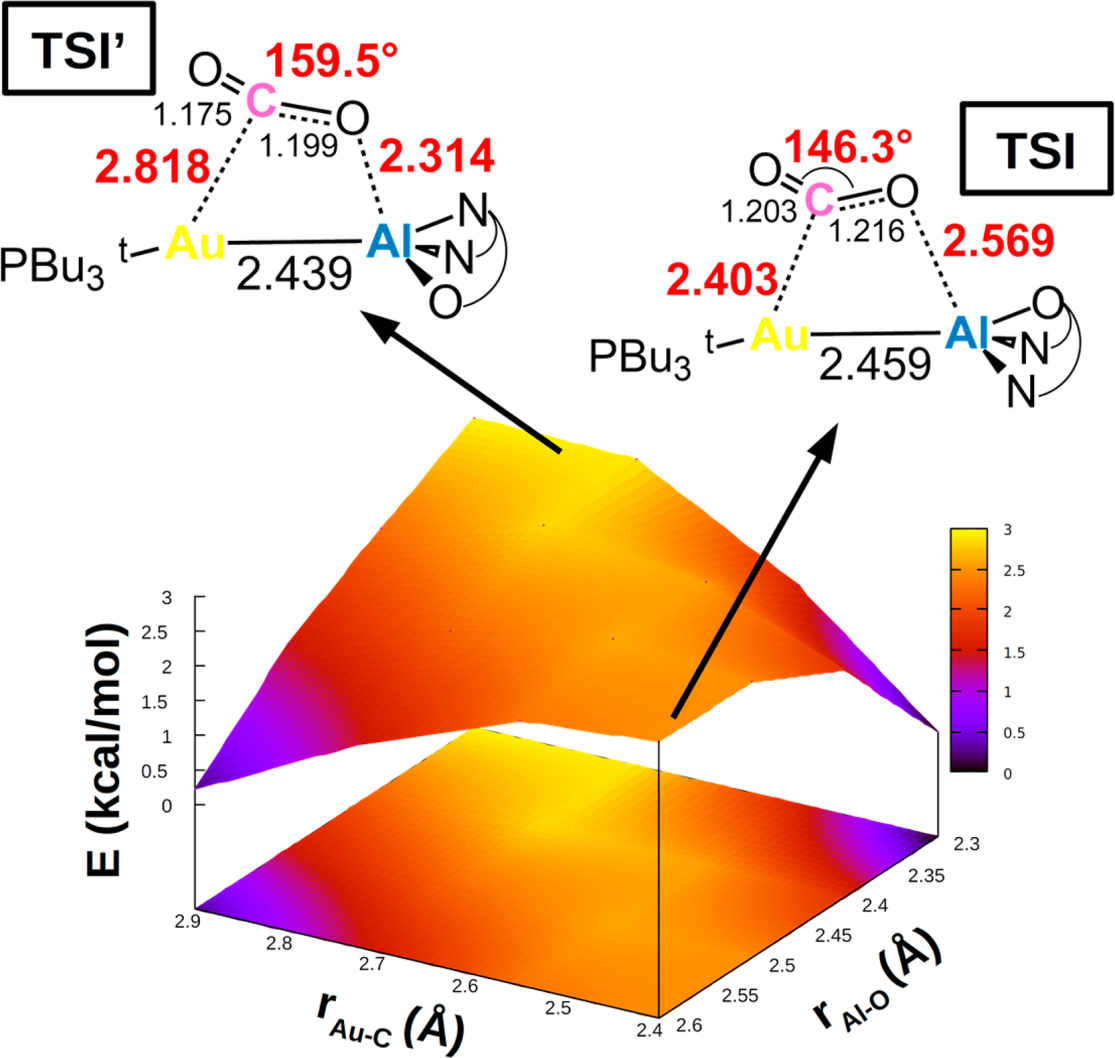
Potential energy surface (PES) in the region neighboring **TSI** for complex **I**. Insets: Position on the PES and schematic structure of **TSI** and **TSI′**. Energy has been shifted in each case according to the minimum energy structure.

It can be clearly surmised that upon substitution of the Group 13 element the reactivity with CO_2_ becomes much more difficult, thus signaling that key differences in the Au–X bond nature should be expected. However, before proceeding in the following sections to detail a comparative analysis of the electronic structures and nature of the Au–X bond in complexes **I**^**X**^, precisely in order to rationalize these findings, we briefly return on the eye-catching differences in the transition state structures of **I** and **I**^**Al**^ (**TSI** and **TSI**^**Al**^, respectively). This point is particularly interesting since the degree of activation of CO_2_ is often monitored by following its bending distortion along the reaction path. For instance, in heterogeneous catalysis studies, the bending of the OCO angle in the surface-adsorbed CO_2_ molecule relative to the gas-phase value of 180° (linear) has been proposed^29^ and widely accepted as a good indicator of activation. The interpretative framework lies on the fact that upon reduction the gas-phase CO_2_ accepts electron charge in its LUMO, which is of antibonding (π*) character and becomes energetically more favored in a bent structure.^2^ However, this is not the case here, since a one-to-one mapping between the OCO angle in **TSI**^**X**^ (and also at **INT**^**X**^) and the activation barrier (and stability) is not found (as discussed above, **I**^**Al**^ features the lowest barrier for the activation and yet the smallest OCO bending angle). To clarify this issue, we explored the topology of the potential energy surface (PES) around each TS, by varying the Au–C and Al–O distances in the 2.90–2.40 and 2.60–2.30 Å ranges, respectively. The PES around **TSI** is shown in Figure 3.

**Figure 4:**
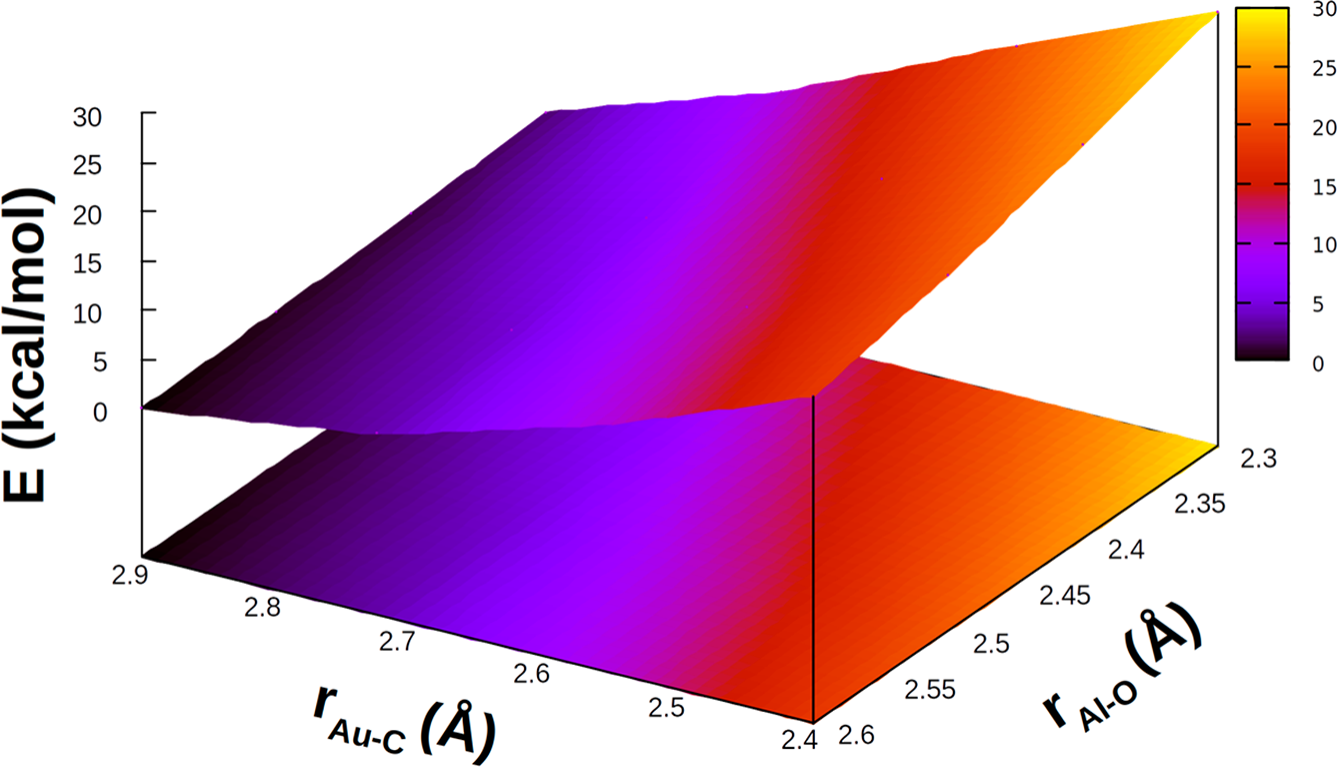
Relative electronic energy of the in-adduct geometry of CO_2_ for each structure sampled in the PES around **TSI**. In each case, energy has been shifted according to the minimum energy structure.

We also explored the PES of carbon dioxide constrained at the geometry of the corresponding structure. The results for **TSI** are reported in Figure 4. The results for the other systems are depicted in Figures S5–S7, and all the numerical values are reported in Tables S2–S5.

The analysis of the PES in Figure 3 is illuminating. It clearly shows that this bimetallic CO_2_ activation occurs in a PES which is very flat around **TSI** in a wide range of interfragment distances, indicating that similar energies correspond to very different structures. Indeed, the energy varies overall in the tight 3 kcal/mol ca. range. This becomes emblematic when considering the structure of CO_2_ in each structure sampled along this PES cut (Figure 4). The CO_2_ bending angle varies significantly along the PES and, in particular, tightens in the direction of both shorter Au–C and Al–O distances. As shown in Figure 4, these changes cause the associated distortion penalty of in-adduct CO_2_ to increase in a much wider range (30 kcal/mol). In other words, in different structures the orbital interactions between **I** and CO_2_ always efficiently counterbalance the variable and increasing distortion penalty of CO_2_.

The complexity of the PES is further confirmed by the fact that we could locate an alternative transition state structure for complex **I** (**TSI′**) with a very different structure with respect to **TSI** (CO_2_ bending angles and the Au–C and Al–O distances are 159.5° and 2.818 and 2.314 Å for the former and 146.3° and 2.403 and 2.569 Å for the latter, respectively) which lies extremely close in energy (**TSI′** is 2.2 and 0.9 kcal/mol higher than **TSI** in terms of electronic and Gibbs’ free energy, respectively) and is almost isostructural with **TSI**^**Al**^. Visualization of the vibrational modes associated with the imaginary frequency of **TSI′** (−212.5 cm^–1^) indicates (analogous to **TSI**) a concerted transition state associated with a vibrational mode involving Au–C, Al–O, and Al–C interactions. We should also mention that in ref (11), using a different computational protocol, a transition state has been reported which is very similar to that of **TSI′** in Figure 3, consistent with the very flat PES we show here.

The PES around **TSI**^**Al**^ (Figure S5) is also flat: Along the same scanned range of Al–O and Au–C distances around **TSI**^**Al**^, an overall variation of 6 kcal/mol is observed, and as can be envisaged by the numerical data reported in Table S3, this range is even tighter right around **TSI**^**Al**^. In the 2.550–2.900 Å and 2.300–2.600 Å ranges for the Au–C and Al–O distances, respectively, two structures with substantial geometrical differences (particularly concerning the CO_2_ structure) have been found with a variation of less than 3 kcal/mol in the electronic energy. Concerning the PESs for **TSI**^**Ga**^ and **TSI**^**In**^, less flat PESs are found, with a variation of 7 and 8 kcal/mol in the overall scanned range, respectively, showing a steeper topology in the closest region to **TSI**^**X**^ (see Figures S6 and S7 and Tables S4 and S5), and suggesting that the orbital interactions between **I**^**Ga**^ and **I**^**In**^ and CO_2_ may not be strong enough to efficiently counterbalance the geometrical distortion of CO_2_.

Notably, as shown in Figure 2, the O–C–O angle is very similar for all the complexes at the INT^X^ structure (124.1, 123.5, and 125.4° for **INT^Al^**, **INT^Ga^**, and **INT^In^**, respectively, and 124.6° for **INT**).^9^ Clearly, this indicates that, as for the intermediate structure, the bending angle of CO_2_ does not represent a good parameter for quantitatively evaluating the capability of the different complexes of activating carbon dioxide, probably due to the cooperative role of Au and X in reacting with CO_2_ (*vide infra*). Very interestingly, these results are in nice agreement with the recent finding that the decrease of the OCO angle is not an appropriate indicator of CO_2_ activation on semiconductor oxides.^30^ As a final remark, we should note that despite the change in the coordination at Al from a tridentate N,O,N′– to a bidentate N,N′– scaffold in **I** and **I**^**Al**^ very similar free energy reaction profiles and PES topology at the TSI have been found. Previous computational results on the electronic structures of the parent naked aluminyls^12^ suggested a possible role of the oxygen atom in the [Al(NON)]^−^ anion and a reduced N-to-Al π donation (“folded” nonplanar [Al(NON)]^−^ vs planar [Al(^Si^NON)]^−^), which seem here to have only a negligible effect on the reactivity of their gold complexes. A detailed analysis of the N-to-Al π donation issue in the aluminyl scaffolds is still lacking, and it certainly deserves to be further investigated.

### Electronic Structure/Reactivity Relationship

To further investigate the effect of the substitution of the Group 13 element on the reactivity discussed above, a comparative analysis of the electronic structure of the transition states and stationary points has been carried out. Several theoretical methods have been applied. In particular, we employed the Activation Strain Model (ASM), Energy Decomposition Analysis (EDA) in combination with ETS-NOCV, Charge Displacement (CD) analysis, and dual descriptors for chemical reactivity.

**Figure 5:**
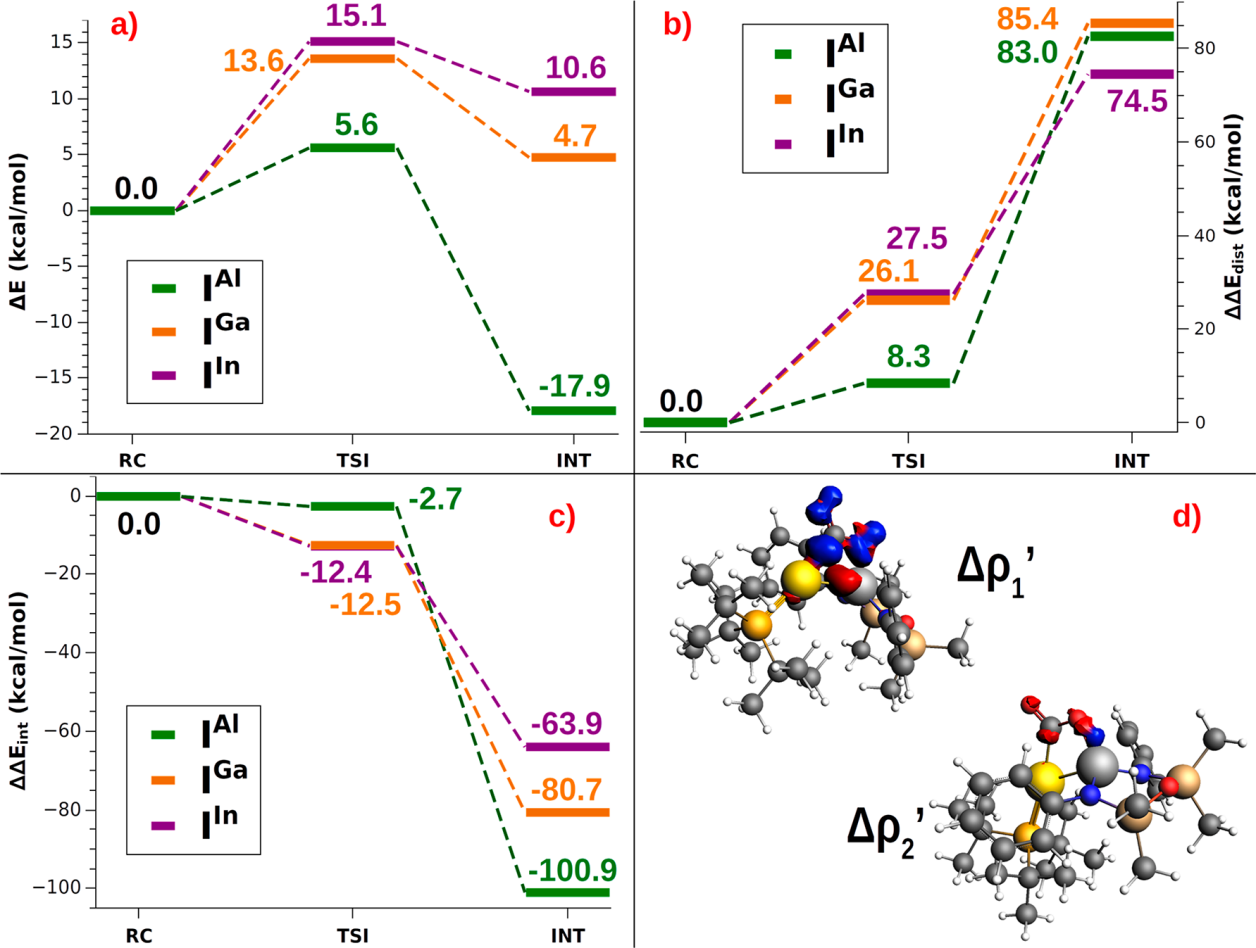
(a) ASM diagrams for the electronic energy variation (Δ*E*) along the reaction path connecting **RC**^**X**^, **TSI**^**X**^, and **INT**^**X**^ structures (X = Al, Ga, and In). (b) ASM diagrams for the variation of the distortion energy penalty (ΔΔ*E*_dist_) along the reaction path connecting **RC**^**X**^, **TSI**^**X**^, and **INT**^**X**^ structures (X = Al, Ga, and In). (c) ASM diagrams for the variation of the interaction energy stabilization (ΔΔ*E*_int_) along the reaction path connecting **RC**^**X**^, **TSI**^**X**^, and **INT**^**X**^ structures (X = Al, Ga, and In). (d) Isodensity surfaces of the main NOCV deformation densities (Δρ_1_′ and Δρ_2_′) for the [CO_2_]–[^t^Bu_3_PAuAl(^Si^NON′)] interaction in **INT**^**Al**^. The isodensity value is 8 me/a_0_^3^ for Δρ_1_′ and 5 me/a_0_^3^ for Δρ_2_′. Charge flux is shown as red → blue. See the “Methodology” section in the Supporting Information for insights into the ASM and ETS-NOCV schemes used here.

We start by decomposing the electronic energy reaction path connecting each **RC**^**X**^ with the corresponding **TSI**^**X**^ and **INT**^**X**^ using the ASM,^31−33^ which decomposes the path into two contributions: the distortion penalty ΔΔ*E*_dist_ of the increasingly deformed reactants and the interaction ΔΔ*E*_int_ between these deformed reactants (see the Supporting Information for details). The ASM allows to get insights into the factors involved in the stabilization/destabilization of **TSI**^**X**^ and **INT**^**X**^ structures. The main results are reported in Figure 5 (Δ*E* energy profile, ΔΔ*E*_dist_, and ΔΔ*E*_int_ in panels a–c, respectively) and discussed below. All the numerical ASM results are reported in Tables S6 and S7.

Upon inspection of Figure 5, we see that the activation barrier for **I**^**Al**^ is the lowest (5.6 kcal/mol) due to an overall small distortion penalty (8.3 kcal/mol), which is efficiently counterbalanced by a small interaction stabilization (−2.7 kcal/mol). For **I**^**Ga**^ and **I**^**In**^, the interaction stabilization (−12.5 and −12.4 kcal/mol, respectively) is not able to efficiently counterbalance the larger distortion penalty (26.1 and 27.5 kcal/mol, respectively), yielding higher activation barriers (13.6 and 15.1 kcal/mol), consistently with the Δ*G*^#^ values discussed in the previous section. As a comparison, the electronic energy activation barrier for **I** (8.9 kcal/mol) was found to result from a 21.8 kcal/mol distortion penalty efficiently counteracted by a −12.9 interaction stabilization.^9^ A complementary picture arises from the analysis of the differences between the three complexes at **INT**^**X**^, where CO_2_ presents a similar degree of distortion. Indeed, as it can be surmised from Figure 5a, the formation of **INT**^**X**^ is only favored for X = Al (Δ*E* = −17.9 kcal/mol), whereas formation of **INT**^**Ga**^ and **INT**^**In**^ is energetically disfavored (Δ*E* = +4.7 and +10.6 kcal/mol, respectively). This is due to the increased distortion penalty which is differently counterbalanced by the interaction stabilization for the three complexes. As it can be seen from Figure 5b, from **RC**^**X**^ to **INT**^**X**^ a similarly increased distortion penalty is observed for all the three complexes, with **I**^**In**^ having a slightly less increased penalty at **INT**^**In**^ with respect to **INT**^**Al**^ and **INT**^**Ga**^ (ΔΔ*E*_dist_ increases up to 85.4, 83.0, and 74.5 kcal/mol for **I**^**Al**^, **I**^**Ga**^, and **I**^**In**^, respectively), consistent with the slightly less bent CO_2_ structure in **INT**^**In**^ (see Figure 2). The interaction stabilization, however, is significantly different, and the extent of the stabilization decreases sharply on going from **INT**^**Al**^ (ΔΔ*E*_int_ = −100.9) to **INT**^**Ga**^ (ΔΔ*E*_int_ = −80.7 kcal/mol) and **INT**^**In**^ (ΔΔ*E*_int_ = −63.9 kcal/mol).

To shed light on the nature of the interactions taking place when CO_2_ approaches **I**^**X**^, we resort to the use of the EDA,^34,35^ and we applied the ETS-NOCV^36^ approach also in combination with the CD^37−39^ function on both **TSI**^**X**^ and **INT**^**X**^ structures. All the results are reported in Tables S8–S9 and Figures S8–S14. The isodensity pictures of the main NOCV deformation densities associated with **INT**^**Al**^ are reported in Figure 5d.

By analyzing the nature of the interactions occurring at the three **TSI**^**X**^ and **INT**^**X**^, the interaction scheme between CO_2_ and the [^t^Bu_3_PAuAl(^Si^NON′)] is qualitatively unaltered, following the same scheme reported in ref (9) for **TSI** (see Figures S8–S14). As an example, for **INT**^**Al**^ in Figure 5d the interaction between the CO_2_ and **I**^**X**^ consists mainly of two opposite charge fluxes: a charge transfer from the Au–X bond toward the LUMO of CO_2_ (Δρ_1_′) and a charge transfer toward the vacant valence np_*z*_ orbital of the Group 13 element from the HOMO of CO_2_ (Δρ_2_′). We mention that the presence of these two active sites, namely, the Au–X bond and the np_*z*_ orbital of X as nucleophilic and electrophilic centers, respectively, can be also visualized in the plot of the dual descriptors (nucleophilicity and electrophilicity) for chemical reactivity, introduced by Morell et al.^40^ (see Figure S15).

The decomposition into the donor and acceptor NOCV orbitals provides a clear picture of the nature of the molecular orbitals (MOs) involved in the interactions depicted above and points out significant differences between the systems under study. Concerning the NOCV deformation density Δρ_1_′ (see Figures S8, S10, and S12), in all cases the main acceptor MO is the LUMO of CO_2_, while the main donor MOs are high-lying σ bonding molecular orbitals of the Au–X complex (namely, HOMO–1 for **I**^**Al**^ and **I**^**Ga**^ and HOMO–2 for **I**^**In**^). However, a quantitative inspection of the atomic composition of these donor MOs reveals that the donor features of complexes **I**^**Ga**^ and **I**^**In**^ differ from those of **I**^**Al**^. Indeed, as shown by the data reported in Table S10, while the energy of the MOs which mainly contribute to the donor NOCV is comparable for all three complexes, their atomic composition varies significantly. The plot and composition of the main donor MO of **I**^**Al**^ (HOMO–1, see Figure S8) clearly suggest an Au–Al-centered σ MO (overall 23.4 and 14.4% contribution from valence s and p orbitals of Al and Au, respectively). However, the HOMO–1 of **I**^**Ga**^ and HOMO–2 of **I**^**In**^ (main donor MOs, see Figures S10 and S12, respectively) are less centered on Au and Ga (16.0 and 4.8% contribution from Ga and Au, respectively) and on Au and In (17.0 and 8.2% contribution from In and Au, respectively), thus indicating a delocalization on the [(^Si^NON′)]^2–^ backbone and suggesting possibly less electron-rich (and less nucleophilic) Au–Ga and Au–In bonds. We quantitatively inspected the extent of the interaction and charge transfer occurring between the CO_2_ and different complexes at **INT**^**X**^ by relying on the EDA, ETS-NOCV, and CD-NOCV approaches. The results are reported in Tables 1 and S9 and Figure S14.

**Table 1:** Main Results of the EDA, ETS-NOCV, and CD Analyses of the [CO_2_]–[^t^Bu_3_PAuX(^Si^NON′)] (X = Al, Ga, and In) Interaction in Intermediates **INT**^**Al**^, **INT**^**Ga**^, and **INT**^**In**^, Respectively^a^

	**INT**^**Al**^	**INT**^**Ga**^	**INT**^**In**^
Δ*E*_oi_	–272.9	–246.2	–193.8
Δ*E*_oi_^1^	–225.3	–204.0	–159.0
|CT^1^|	0.67	0.61	0.60
Δ*E*_oi_^2^	–11.1	–11.9	–9.8
|CT^2^|	0.07	0.07	0.07
Δ*E*	–105.9	–84.8	–68.1

a Energies are given in kcal/mol; charge transfer (CT) values are given in electrons (e).

From a quantitative perspective, data in Table 1 show that the activation process is favored for **I**^**Al**^ over **I**^**Ga**^ and **I**^**In**^. A much stronger interaction between **I**^**Al**^ and CO_2_ is observed at **INT**^**Al**^ (−105.9 kcal/mol) with respect to **I**^**Ga**^ and **I**^**In**^ (−84.8 and −68.1 kcal/mol). The same trend can be noticed for the orbital interaction energy Δ*E*_oi_^1^ and the Au–X bond-to-CO_2_ charge transfer |CT^1^| values (0.67, 0.61, and 0.60 e for **INT**^**Al**^, **INT**^**Ga**^, and **INT**^**In**^, respectively), in Table 1. Remarkably, the three complexes display very similar Δ*E*_oi_^2^ and |CT^2^| values. These results show that the interaction between Au and the Group 13 element does change on descending along the group. The decomposition of NOCVs into MOs presented above suggests that Ga and In may feature a more polarized Au^δ+^–X ^δ−^ bond, which would be consistent with the weaker activation/insertion product stabilization ability in a diradical-like reactivity with CO_2_.^9^ An appropriate bonding analysis is needed to quantitatively assess this hypothesis. The results are presented in the next section.

### Features of the Au–X (X = Al, Ga, and In) Bond and the Impact on the Diradical-like Reactivity

The analysis of the features of the bond between gold and the Group 13 fragments may shed light into the differences in the reactivity observed above. To this aim we use a three-pronged approach: (i) We analyze the nature of the Au–X bond in complexes **I**^**X**^ with the CD-NOCV and ETS-NOCV approaches in order to identify the basic nature of the Au–X in these complexes and the degree of polarization on descending along the Group 13. This method has been successfully applied to complex **I** where revealed the existence of an electron-sharing Au–Al bond. (ii) We assess the variability in the electronic structure of the [X(^Si^NON′)] fragments, by relying on the tools of conceptual DFT^41,42^ (i.e., studying the nature of the lone pairs of the anions by calculating their gas phase proton affinity and quantitatively comparing their HOMO energy). (iii) To assess the radical-like behavior of complexes **I**^**X**^ (if any) and its impact on the formation of the products **II**^**X**^, we model the reactivity of the radical fragments [X(^Si^NON′)]· and [^t^Bu_3_PAu]· with carbon dioxide.

**Figure 6:**
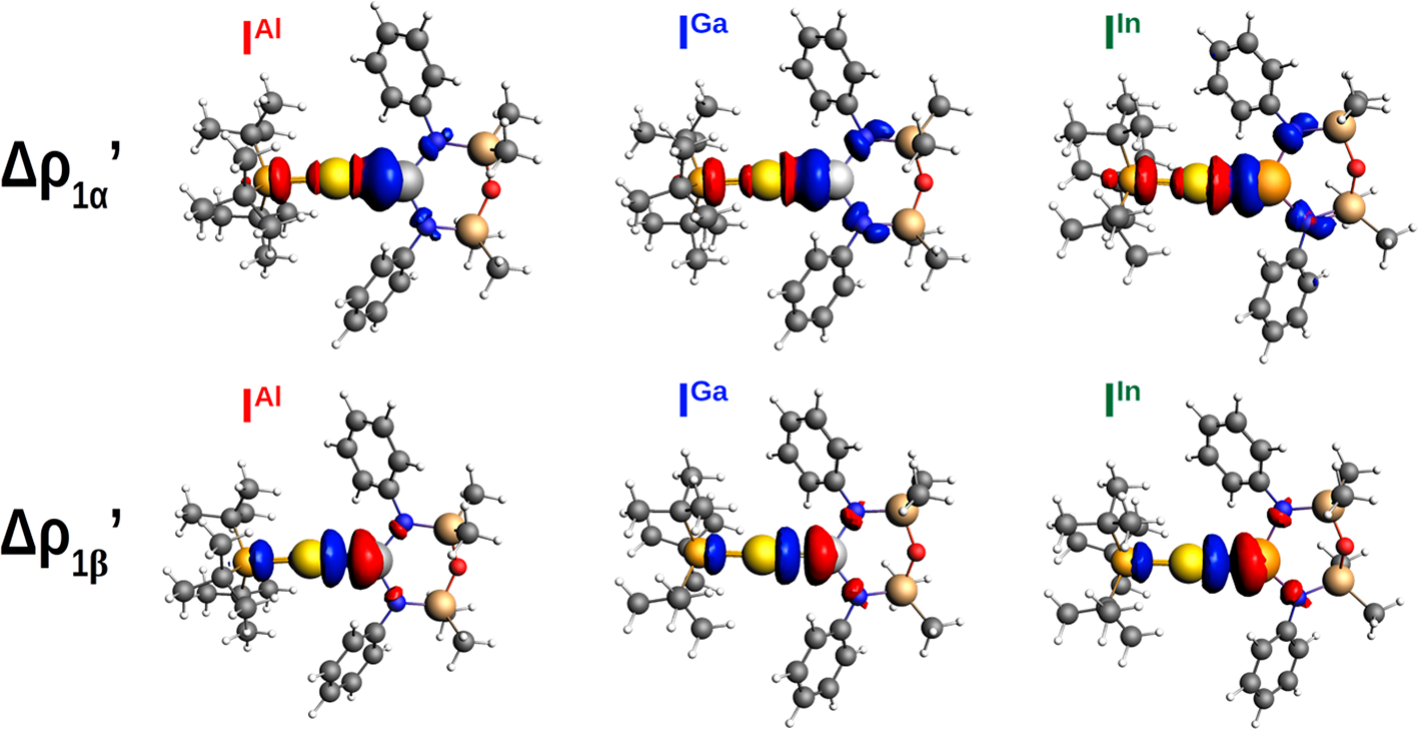
Isodensity surfaces (isodensity value 2 me/a_0_^3^) of the main NOCV deformation densities (Δρ_1α_′ and Δρ_1β_′) for the interaction between doublet [^t^Bu_3_PAu]· and [X(^Si^NON′)]· fragments (X = Al, Ga, and In) in complexes **I**^**Al**^, **I**^**Ga**^, and **I**^**In**^. The charge flux is shown as red → blue.

**Figure 7:**
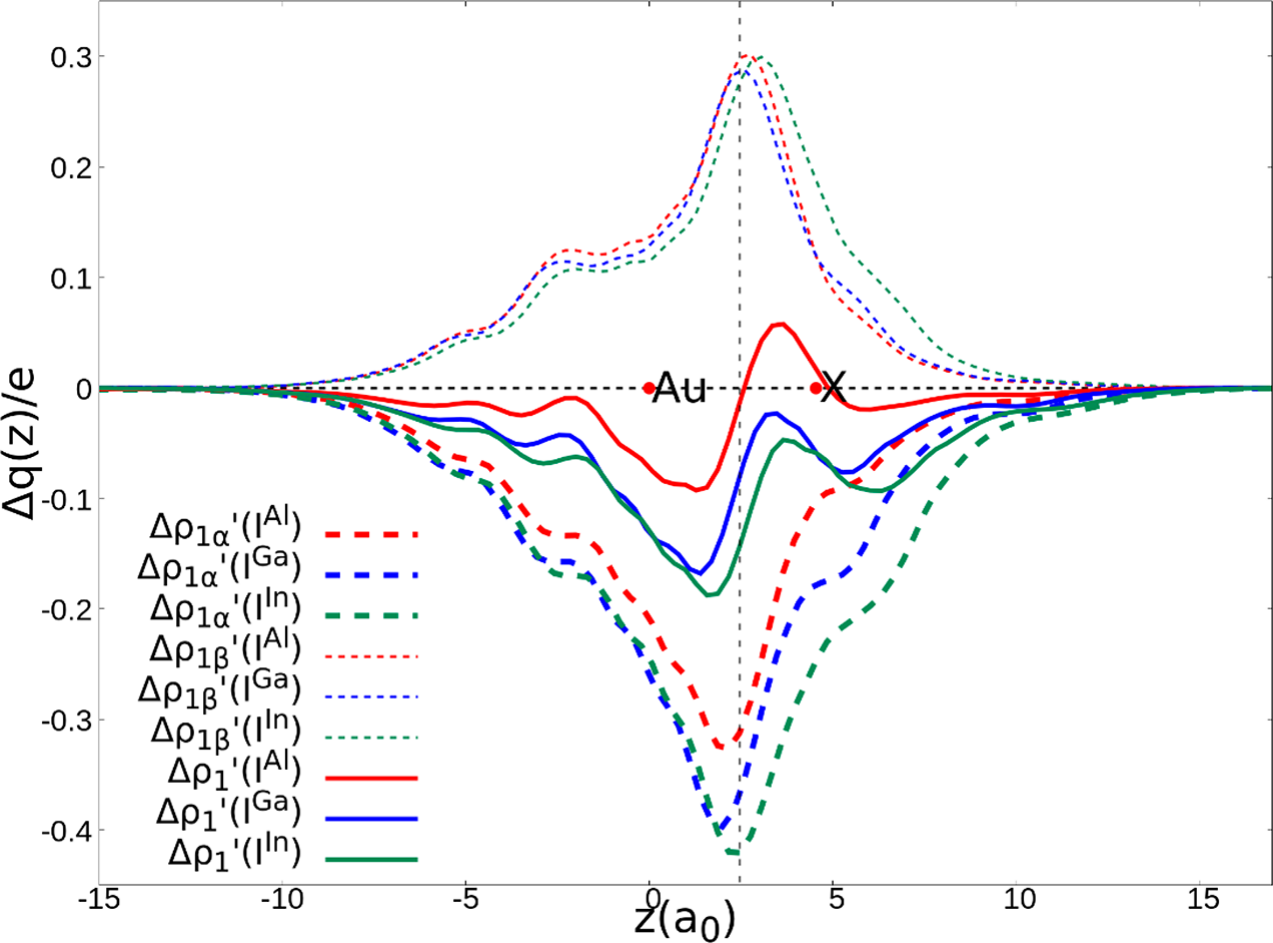
Charge displacement (CD-NOCV) curves associated with the Δρ_1α_′ and Δρ_1β_′ NOCV deformation densities (negative dashed curves and positive dashed curves, respectively) for the interaction between neutral doublet [^t^Bu_3_PAu]· and [X(^Si^NON′)]· fragments (X = Al, Ga, and In) for complexes **I**^**Al**^, **I**^**Ga**^ and **I**^**In**^. The overall Δρ_1_′ curves (solid curves) are also reported. Red dots indicate the average position of the nuclei along the *z*-axis. The vertical dashed line marks the average position of the isodensity boundary between the fragments. Positive (negative) values of the curve indicate right-to-left (left-to-right) charge transfer.

In order to apply the CD-NOCV and ETS-NOCV approaches in a consistent way it is important to determine the most suitable fragmentation (i.e., charged singlet or neutral doublet [^t^Bu_3_PAu] and [X(^Si^NON′)] fragments) for the most accurate description of the bond. Such an assessment, carried out with the protocol reported in refs (43) and (44), which is based on a comparative EDA, is shown in Tables S11–S13. It demonstrates that for all the complexes the most appropriate fragments for describing the Au–X bond are the neutral doublet fragments [^t^Bu_3_PAu]· and [X(^Si^NON′)]· since this fragmentation provides both the smaller orbital interaction and total interaction energies. Application of the NOCV-CD approach allows to quantify the differences between the three Au–X bonds, as shown in Figures 6 and 7 and Table 2. The complete results of the NOCV-CD analysis using neutral doublet fragments are reported in Table S14 and Figures S16–S21.

**Table 2:** Orbital Interaction Energies (Δ*E*_oi_^*k*^) and Charge Transfer (CT^*k*^)^a^

	Δ*E*_oi_^1α^	CT^1α^	Δ*E*_oi_^1β^	CT^1β^	CT^1^
**I**^**Al**^	–33.1	–0.31	–23.4	0.30	–0.01
**I**^**Ga**^	–47.4	–0.37	–21.5	0.29	–0.08
**I**^**In**^	–45.3	–0.42	–20.3	0.26	–0.16

a Associated to the first NOCV deformation density and to the corresponding α and β components of the interaction between neutral doublet [^t^Bu_3_PAu]· and [X(^Si^NON′)]· fragments (X = Al, Ga, and In) for complexes **I**^**Al**^, **I**^**Ga**^ and **I**^**In**^.

From a qualitative perspective, the bond picture between the [^t^Bu_3_PAu]· and [X(^Si^NON′)]· fragments does not change upon substitution of the Group 13 element X. It consists mainly of two components that act in opposite directions: a gold-to-X (Δρ_1α_′) and an X-to-gold (Δρ_1β_′) charge transfer of spherical (σ) symmetry. In addition to these, two dative gold-to-X π back-donation components are also envisioned in each case (see Figures S16–S21), although their contribution is considerably smaller.

From a quantitative point of view, however, the amount of the two opposite charge fluxes changes substantially on descending along Group 13, as it can be clearly seen from the shape and extent of the CD-NOCV curves in Figure 7 and data in Table 2. By comparing the CD-NOCV curves, it is very clear that the gold-to-X (Δρ_1α_′) charge transfer increases in the order Al < Ga < In, thus suggesting that the polarization of the Au–X bond may increase on descending along the group. Quantitatively, for the [Al(^Si^NON′)]· fragment, we see indeed that the two fluxes are practically equivalent (associated CT values of −0.31 and 0.30 e for Δρ_1α_′ and Δρ_1β_′, respectively), resulting in an overall very small net charge transfer (−0.01 e) that can be ascribed to the presence of a slightly polarized electron-sharing Au–Al bond. This picture is also very similar to that of the [^t^Bu_3_PAu]–[Al(NON′)] bond reported in ref (9) and to that of a nonpolar covalent bond system, such as the homonuclear Au_2_ molecule (see Figure S8 in the Supporting Information of ref (9).).

For the bond involving the [Ga(^Si^NON′)]· fragment, while the extent of the Ga-to-Au donation remains practically unaltered (0.29 e), the Δρ_1α_′ component related to the Au-to-Ga charge increases (−0.37 e), thus representing a more polarized Au^δ+^–Ga^δ−^ bond (net charge transfer −0.08 e). For the [^t^Bu_3_PAu]·–[In(^Si^NON′)]· bond, the difference is even more pronounced, with a CT^1α^ of −0.42 e for the donation toward indium and a more negative net charge transfer (−0.16 e). The associated Δ*E*_oi_^1α^ values follow the same trend, with **I**^**Al**^ having the less negative value (−33.1 kcal/mol) which increases for both **I**^**Ga**^ and **I**^**In**^ (−47.4 and −45.3 kcal/mol, respectively).

The different tendency of Al, Ga, and In to form electron-sharing type bonds with Au can be further inferred by relying on the tools of conceptual DFT^41,42^ (see the “Methodology” section in the Supporting Information for details). By using global DFT descriptors (Table S15) on the neutral species, one can easily see that [Ga(^Si^NON′)]· and [In(^Si^NON′)]· are more likely to retain the negative charge with respect to the [Al(^Si^NON′)]· fragment. For instance, the electrophilicity (ω^–^) index peaks at the indium fragment (2.58) and descends toward gallium (2.15) and more rapidly toward the [Al(^Si^NON′)]· fragment (1.68), consistent with the increased tendency of gallium and indium fragments to accept electrons from gold. The nucleophilicity (*N*) index follows, coherently, the opposite trend, decreasing from Al toward In (values are 0.17, 0.14, and 0.13 for Al, Ga and In fragments, respectively).

**Figure 8:**
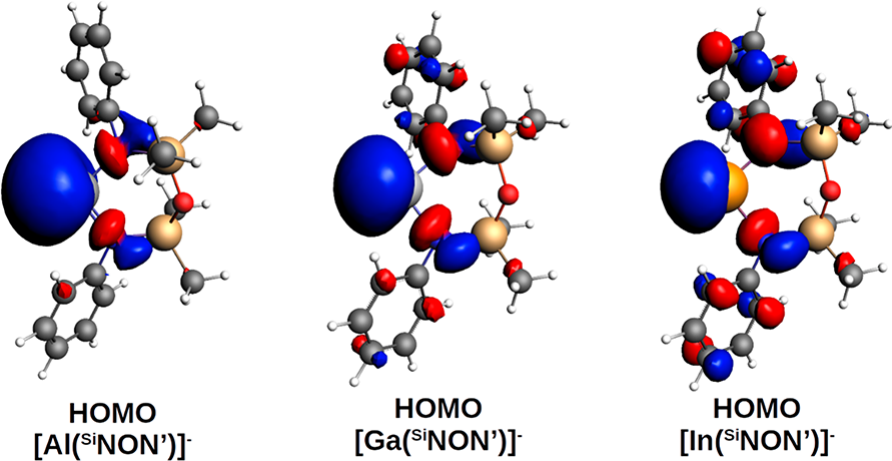
Isodensity pictures of the HOMO of the three [X(^Si^NON′)]^−^ (X = Al, Ga, and In) anions. Isovalue is 30 me/a_0_.

The evaluation of the strength and basicity of the lone pair of the [X(^Si^NON′)]^−^ anions also gives an idea of the different nature of these species.^45^ On the basis of the gas phase proton affinity and the corresponding HOMO energy and composition properties, the [Al(^Si^NON′)]^−^ fragment emerges as the most reactive and basic anion (proton affinity −354.5 kcal/mol vs −334.8 and −294.2 kcal/mol for [Ga(^Si^NON′)]^−^ and [In(^Si^NON′)]^−^, respectively, see Table S16). The peculiarity of the [Al(^Si^NON′)]^−^ lone pair with respect to those of [Ga(^Si^NON′)]^−^ and [In(^Si^NON′)]^−^ is immediately evident by inspection of the isodensity pictures of the corresponding HOMOs, reported in Figure 8.

**Scheme 2:**
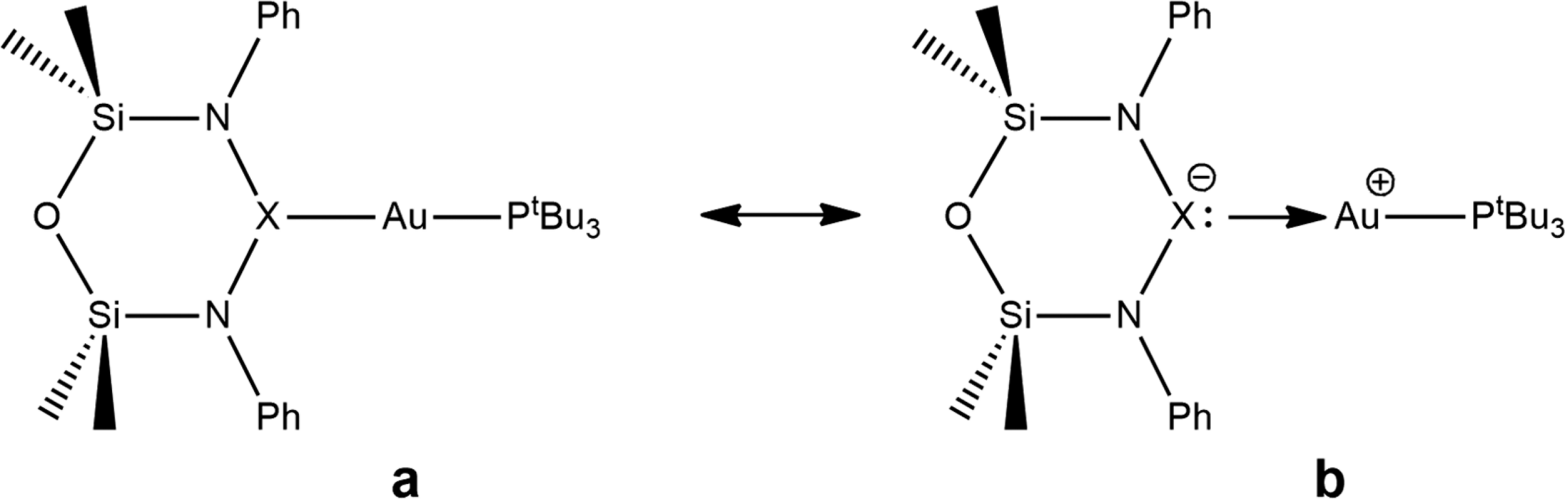
Resonance Canonical Structures Representing a purely electron sharing (structure **a**) and a purely dative (structure **b**) bond between Au and X (X = Al, Ga, and In).

The [Al(^Si^NON′)]^−^ HOMO is very diffuse and mainly centered at the Al site, whereas on descending toward Ga and In, the HOMO becomes much less diffuse and more delocalized on the (^Si^NON′)^2–^ ligand. The HOMO energy and nature for these anions are also consistent with this trend and with the proton affinities trend: the HOMO energy for the aluminyl is the highest (−0.356 eV), whereas gallyl and indyl anions have more stabilized HOMOs (−1.095 and −1.320 eV, respectively), thus reflecting the higher basic character of the aluminyl. Consistently, while the aluminyl HOMO has atomic contributions mostly from s and p orbitals of Al (more than 80%), the gallyl and indyl HOMO contains analogous contributions, but to a much lesser extent (in both cases below 50%), thus indicating more ligand-centered MOs, coherently with their lower basic power and reactivity (see Table S16). On the basis of these results, two canonical resonance structures can be drawn for explaining the changing bonding scheme, as depicted in Scheme 2.

Structure **a** in Scheme 2 represents a purely electron-sharing bond between Au and X, while canonical structure **b** refers to a polarized dative-type bond in which the fragment bearing X behaves more like a ligand toward gold. On the basis of the CD-NOCV analysis, it is clear that in complex **I**^**Al**^ an almost pure electron-sharing type bond between Au and Al occurs, with structure **a** being the dominant one. Such a bond is only slightly polarized, which is consistent with its remarkable ability of activating carbon dioxide as a nucleophile (see also the previously discussed Au and Al centered donor MOs). For complexes **I**^**Ga**^ and **I**^**In**^, while structure **a** is still dominant, the weight of structure **b** progressively increases: The two gallium and indium fragments have a higher tendency to retain their anionic character, which makes the Au–X bond more polarized and with a decreased electron-sharing character.

At this stage, it is worth investigating if such differences in the Au–X bond reflect into differences on the radical-like behavior of the fragments (which is an expected behavior, at least for complex **I**^**Al**^, based on the results reported in ref (9) and on the mechanism depicted in the previous section). Indeed, for the [^t^Bu_3_PAuAl(NON′)] complex,^9^ the electron-sharing character of the Au–Al bond was strictly related to the two fragments behaving cooperatively like radicals for the carbon dioxide insertion, leading to the [^t^Bu_3_PAuCO_2_Al(NON′)] product. Since a similar insertion product has been also calculated for complexes **I**^**Al**^, **I**^**Ga**^, and **I**^**In**^, an analogous geometric and energetic assessment of the radical-like behavior can be carried out.

**Scheme 3:**
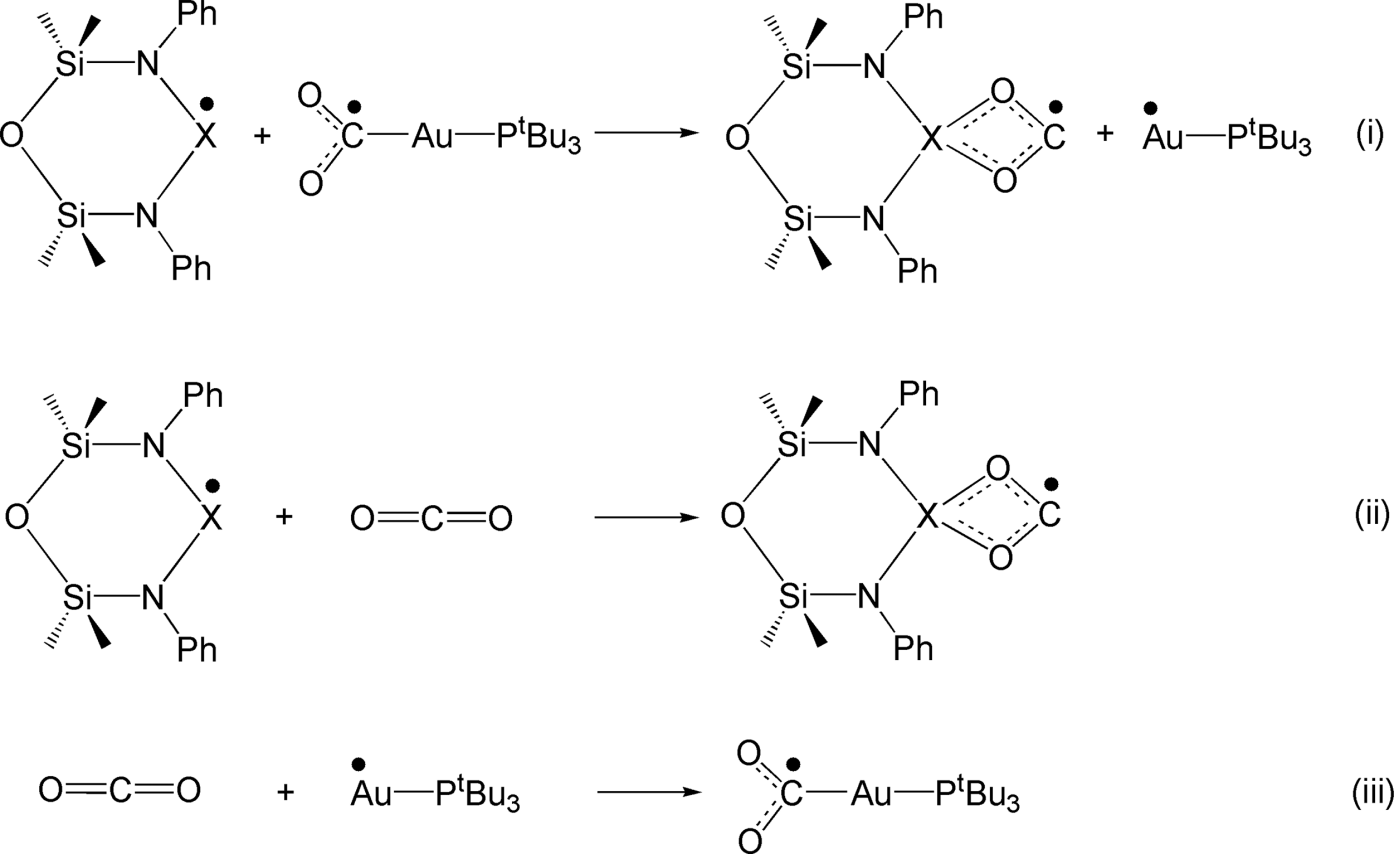
Exchange (i) and Association (ii) and (iii) Reactions Involving Open Shell Neutral Radical [X(^Si^NON′)]· (X = Al, Ga, and In) and [^t^Bu_3_PAu]· Fragments Reacting with CO_2_

From a structural perspective, optimization of the open shell radical [CO_2_X(^Si^NON′)]· leads to structures that are closely reminiscent of the in-adduct structures of the insertion products [^t^Bu_3_PAuCO_2_X(^Si^NON′)], with the main structural parameters of the CO_2_ coordination being quantitatively similar (see the schematic representation in Figure S22). On the basis of dissociation/association reactions (i–iii) involving these open shell species interacting with each other and with carbon dioxide, we were able to quantitatively shed light on the differences between the three Group 13 species, as displayed in Scheme 3 and Table 3.

**Table 3:** Reaction Energies (Δ*E*) for Exchange (i) and Association (ii) and (iii) Reactions Involving Open Shell Neutral Radical [X(^Si^NON′)]· (X = Al, Ga, and In) and [^t^Bu_3_PAu]· Fragments Reacting with CO_2_

	Δ*E* (kcal/mol)		
reaction	X = Al	X = Ga	X = In
(i)	–2.2	17.9	27.5
(ii)	–12.5	7.7	17.3
(iii)	[^t^Bu_3_PAu]·		
	–10.3		

Calculation of the energies for the exchange reaction (i) between the [X(^Si^NON′)]· and [^t^Bu_3_PAu]· fragments, with the formation of the [CO_2_X(^Si^NON′)]· species, points out that the [CO_2_Ga(^Si^NON′)]· and [CO_2_In(^Si^NON′)]· fragments are less stable with respect to [CO_2_Al(^Si^NON′)]·. Indeed, only for X = Al do we observe a negative Δ*E* for the exchange (−2.2 kcal/mol), while positive values are calculated for X = Ga and In (+17.9 and +27.5 kcal/mol, respectively). This is consistent with the much lower affinity of the Ga and In radical fragments toward CO_2_ (positive Δ*E*s of 7.7 and 17.3 kcal/mol for (ii), respectively) with respect to Al (Δ*E* = −12.5 kcal/mol). Notably, the results for reaction (iii), which depict the favorable formation of the [^t^Bu_3_PAuCO_2_]· species (Δ*E* = −10.3 kcal/mol) are clearly consistent with the cooperative radical-like behavior of Al and Au.

These results clearly point out that while in all the cases the observed product is in accordance with a diradical-like cooperative reactivity of Au and X the [Al(^Si^NON′)]· fragment has a higher affinity toward CO_2_, consistent with the exergonic formation of the insertion product and with the spin density of the radical (Figure S23), for which the unpaired electron is practically entirely localized on Al (0.97). However, the [Ga(^Si^NON′)]· and [In(^Si^NON′)]· radicals have a decreased affinity toward CO_2_, consistent with a more delocalized spin density (0.70 and 0.59 e on Ga and In, respectively) and with the endergonic formation of the corresponding insertion products. These findings are fully coherent with those recently reported for lithium– and zinc–aluminyl complexes.^10^ Indeed, the latter, featuring a highly covalent and electron-rich Zn–Al bond, has been shown to react with CO_2_ leading to an insertion product very similar to **II** and **II**^**X**^, thus further corroborating the importance of electron-rich M–X bonds with a highly electron-sharing character for the reactivity with carbon dioxide.

**Figure 9:**
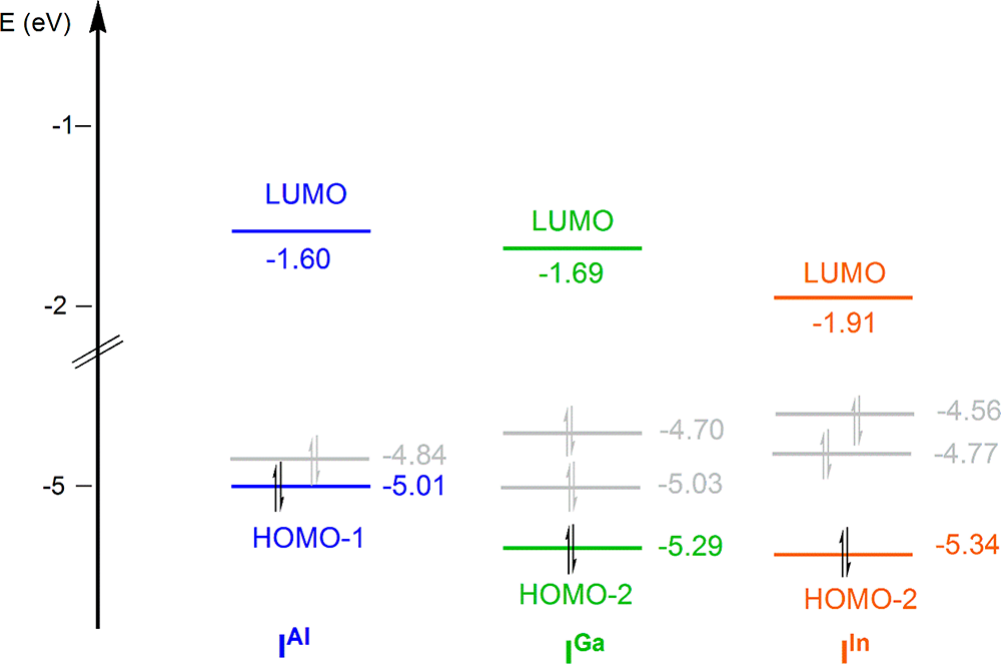
Calculated LUMO (MO associated with the Al/Ga/In empty p-orbital), HOMO and HOMO-*n* energies (HOMO-*n* indicates the Au–X σ bonding MO) of complexes **I**^**X**^ (X = Al, Ga, and In). Orbital energies are given in eV.

We would like to underline that the above results can be hardly inferred from a simple “frontier” MOs diagram. The electronic structures for the series of **I**^**X**^ complexes are depicted in Figure 9, where the energies of the key occupied and virtual orbitals involved in interactions with CO_2_ are highlighted.

The HOMO–LUMO energy gap decreases from **I**^**Al**^ (3.24 eV) to **I**^**Ga**^ (3.01 eV), with **I**^**In**^ featuring the smallest value (2.65 eV), thus suggesting a stability trend of **I**^**Al**^ > **I**^**Ga**^ > **I**^**In**^. More important in terms of reactivity is the energy separation between the key occupied and unoccupied MOs (HOMO–1 and LUMO for **I**^**Al**^, HOMO–2 and LUMO for **I**^**Ga**^ and **I**^**In**^), which is very similar for all the three complexes. However, a correlation can be found for the occupied donor MO energies, with **I**^**Al**^ showing the highest energy donor HOMO–1 (−5.01 eV), and **I**^**Ga**^ and **I**^**In**^ showing a lower energy donor HOMO–2 (−5.29 and −5.34 eV, respectively), consistent with the lowest first step barrier calculated for **I**^**Al**^.

Finally, the possibility that the gold–aluminyl complex would be better described as a diradical rather than a closed-shell singlet has been explored. The reaction profile for complex **I** has been calculated at the open-shell (unrestricted) singlet level, attaining the same geometries and energies as those calculated at closed-shell (restricted) one, whereas the triplet spin reaction profile is much higher in energy. For instance, the electronic energy difference between the singlet and triplet spin states for complex **I** is 66.6 kcal/mol.

## Conclusions

In this work, we have computationally investigated the reactivity toward carbon dioxide and the electronic structure of a series of isostructural gold complexes, in which the gold center is coordinated to a heterocyclic anion of Group 13 elements (Al, Ga, and In).

Both the reaction mechanism and the trends in the electronic structure along the reaction path of all the complexes reveal that the gold–aluminyl complex represents a peculiar case. Indeed, it features the lowest activation barriers and is the only complex for which the insertion product formation is calculated to be exothermic. This different reactivity with CO_2_ reflects changes in the electronic structure of these compounds upon element substitution. By investigating the interactions taking place along the reaction path, it clearly emerges that while in all the cases the same mechanism is observed (i.e., Au–X bond acting as a nucleophilic site coupled with the vacant np_*z*_ orbital of the Group 13 element behaving as an electrophilic site), the gold–aluminyl bond is the most apolar electron-sharing-type bond featuring an enhanced capacity of activating and stabilizing carbon dioxide. All the other Au–X bonds show a decreasing electron-sharing character with an increasing Au^δ+^–X^δ−^ polarization when descending along the group toward the heavier elements Ga and In, which, in turn, makes the activation of CO_2_ much harder both kinetically and thermodynamically. Aluminyl anion is so special because of the highly electron-sharing nature of the Au–Al bond. The decreasing electron-sharing character for gallyl and indyl gold complexes accounts for the endergonic formation of their carbon dioxide insertion products, thus showing that a radical-like reactivity is crucial for CO_2_ capture.

This work fits in the framework of new perspectives on this novel and unconventional reactivity, highlighting the singularity of the aluminyl anions and, more generally, revealing that the kinetics and thermodynamics of TM–X cooperative processes for the activation of carbon dioxide are strictly related to the degree of the electron-sharing character of the TM–X bond, which represents a critical factor for the rational control of this reactivity. In an even more general framework, we also find that carbon dioxide bending is not a good indicator of its activation, similar to what is currently emerging in heterogeneous catalysis, making these TM–X complexes promising good models for studying CO_2_ fixation into nucleophilic/electrophilic sites-containing heterogeneous catalysts.

## Computational Details

All geometry optimizations and frequency calculations on optimized structures (minima with zero imaginary frequencies and transition states with one imaginary frequency) for the CO_2_ insertion into the [^t^Bu_3_PAuX(^Si^NON′)] (X = Al, Ga, and In) complexes reaction have been carried out using the Amsterdam Density Functional (ADF) code^46,47^ in combination with the related Quantum-regions Interconnected by Local Description (QUILD) program.^48^ The PBE^49^ GGA exchange-correlation (XC) functional, the TZ2P basis set with a small frozen core approximation for all atoms, the ZORA Hamiltonian^50−52^ for treating scalar relativistic effects, and the Grimme’s D3-BJ dispersion correction were used.^53,54^ Solvent effects were modeled employing the Conductor-like Screening Model (COSMO) with the default parameters for toluene as implemented in the ADF code.^55^ The same computational setup has also been used for the EDA, CD-NOCV, and ASM calculations and for computing the radical reactions between [X(^Si^NON′)], [CO_2_], and [^t^Bu_3_PAu] fragments. Gas-phase calculation of conceptual DFT descriptors and proton affinities have been carried out by excluding solvent effects from the same computational protocol. This protocol has been used successfully in refs (3) and (9) to study the [^t^Bu_3_PAuAl(NON)] and [^t^Bu_3_PAuCO_2_Al(NON)] complexes. For further details and description of the methods used in this work, see the “Methodology” section in the Supporting Information.

## Supporting Information Available

Methodology, imaginary frequencies of **TSI**^**X**^ and **TSII**^**X**^, energies associated with the PESs, ASM results, EDA, ETS-NOCV and NOCV-CD analysis of **TSI**^**X**^ and **INT**^**X**^, energy and composition of donor MOs of **I**^**X**^, comparative EDA for complexes **I**^**X**^, NOCV results on **I**^**X**^, conceptual DFT results on Au and X fragments, proton affinity and anion’s HOMO analysis of [X(^Si^NON′)]^−^, optimized structures of **I**^**X**^, **RC**^**X**^, **TSI**^**X**^, **INT**^**X**^, **TSII**^**X**^, and **PC**^**X**^ complexes, PESs around **TSI**^**X**^, NOCV analysis of **TS**^**X**^, NOCV analysis of **INT**^**X**^, dual descriptor plots, CD-NOCV results on **I**^**X**^, schematic geometries of radical [CO_2_X(^Si^NON′)] fragments, spin densities of [X(^Si^NON′)] radicals, *xyz* coordinates of all structures (PDF)
