# Supplementary material for: What Singles out Aluminyl Anions? A Comparative Computational Study of the Carbon Dioxide Insertion Reaction in Gold–Aluminyl, −Gallyl, and −Indyl Complexes
Source: Inorg Chem. 2022 Jan 6;61(3):1704–16. doi: 10.1021/acs.inorgchem.1c03579 (PMC8790757; doi:10.1021/acs.inorgchem.1c03579)
Supplement: Supplementary file 1 — ic1c03579_si_001.pdf [file ic1c03579_si_001.pdf]

Supporting Information

What singles out aluminyl anions? A comparative computational study of the carbon dioxide insertion reaction in gold-aluminyl, -gallyl and -indyl complexes

Diego Sorbelli,[*,†] Leonardo Belpassi,[*,‡] and Paola Belanzoni[*,†,‡]

[†]*Department of Chemistry, Biology and Biotechnologies, University of Perugia, Via Elce di Sotto, 8 – 06123, Perugia, Italy*

[‡]*CNR Institute of Chemical Science and Technologies "Giulio Natta" (CNR-SCITEC), Via Elce di Sotto, 8 – 06123, Perugia, Italy*

Index of contents

Methodology 2
Figures S1-S4. Optimized structures 8
Table S1. Imaginary frequencies of $\mathbf{TSI^X}$ and $\mathbf{TSII^X}$ 12
Figures S5-S7. PESs around $\mathbf{TSI^X}$ 13
Tables S2-S5. Energies associated to the PESs. 15
Tables S6-S7. ASM results 17
Tables S8-S9. EDA, ETS-NOCV and NOCV-CD analysis of $\mathbf{TSI^X}$ and $\mathbf{INT^X}$ 18
Figures S8-S13 NOCV analysis of $\mathbf{TS^X}$ 19
Figure S14. NOCV analysis of $\mathbf{INT^X}$ 25
Figure S15. Dual descriptor plots 26
Table S10. Energy and composition of donor MOs of $\mathbf{I^X}$ 27
Tables S11-S13. Comparative EDA for complexes $\mathbf{I^X}$ 28
Table S14. NOCV results on $\mathbf{I^X}$ 30
Figures S16-S21. CD-NOCV results on $\mathbf{I^X}$ 31
Table S15. Conceptual DFT results on Au and X fragments 37
Table S16. Proton affinity and anion's HOMO analysis of $[X(^{Si}NON)]^-$ 37
Figure S22. Schematic geometries of radical $[CO_2X(^{Si}NON')]$ fragments 38
Figure S23. Spin densities of $[X(^{Si}NON')]$ radicals 38
References 39
xyz structures 41

- **Natural Orbitals for Chemical Valence and Charge Displacement analysis**

The Natural Orbitals for Chemical Valence (NOCV)[1,2] is a suitable approach for the description of chemical bonding based on the rearrangement of the electron density occurring when a chemical bond is formed. Such arrangement can be expressed as electron density difference between the formed adduct (AB) and sum of the densities of the two non-interacting fragments (A and B) frozen in their adduct geometries.

This deformation density can be brought into diagonal contributions in terms of NOCVs. In the NOCV scheme, the charge rearrangement taking place upon bond formation is obtained from the occupied orbitals of the two fragments suitably orthogonalized to each other and renormalized (*promolecule*). The resulting electron density rearrangement ($\Delta\rho'$) can be expressed in terms of NOCV pairs which are defined as the eigenfunctions of the so-called "valence operator"[3–5] as follows:

$$\Delta\rho' = \sum_k \nu_k(|\phi_{+k}|^2 - |\phi_{-k}|^2) = \sum_k \Delta\rho'_k \quad \text{[S1]}$$

where ϕ_{+k} and ϕ_{-k} are the NOCV pairs orbitals and $\nu_{\pm k}$ are the corresponding eigenvalues. When the adduct is formed from the promolecule, a fraction ν_k of electrons is transferred from the ϕ_{-k} to the ϕ_{-k} orbital, which are envisaged as donor and acceptor orbitals, respectively. For the sake of interpretation, a population analysis can also be performed in order to single out, for ϕ_{-k} and ϕ_{+k} orbitals, which molecular orbitals (MOs) of the two constituting fragments contribute to the interaction (with a resulting associated coefficient accounting for the magnitude of the contribution).

The NOCV scheme can be coupled with the framework of the Charge Displacement (CD)[6] analysis. The CD analysis allows to quantify the amount of electronic charge that is transferred between the two fragments upon the formation of the A-B bond. The Charge Displacement function (Δq) is defined as the partial progressive integration on a suitable z-axis of the deformation density $\Delta\rho'$:[7]

$$\Delta q(z) = \int_{-\infty}^{z} dz' \int_{-\infty}^{+\infty}\int_{-\infty}^{+\infty} \Delta\rho'(x,y,z')\,dx\,dy \quad \text{[S2]}$$

The CD function, $\Delta q(z)$, quantifies at each point of the bond axis the exact amount of electron charge that, upon formation of the bond, is transferred from the right to the left across a plane perpendicular to the bond axis through z.

When coupled with the NOCV scheme, the density rearrangement due to the bond formation between two fragments, ($\Delta\rho'$), can be partitioned in different NOCV deformation densities ($\Delta\rho'_k$) and therefore one is able to quantify the charge transfer (CT) associated to each different component. It must be noted that only few of the NOCV pairs contributes to the chemical bond. Therefore, when the CD-NOCV analysis is carried out, usually only the first $\Delta\rho_k'$ components are investigated in order to understand which significant chemical contribution to the bond they represent.

In equation [S2], the integration axis is usually conveniently chosen as the bond axis between the two fragments constituting the adduct and usually we choose to evaluate the charge transfer between A and B by taking the CD value at the "isodensity boundary", i.e. the z-point where equally valued isodensity surfaces of the isolated fragments become tangent.[7,8]

In this case, since we also apply this scheme to the transition state TSI, with [^{t}Bu$_3$PAuX(^{Si}NON')] (X=B,Al,Ga,In) and [CO_2] as fragments, such approach is complicated, since the two fragments display multiple interactions with multiple atomic centers and thus it is clearly impossible to define a unique bond axis and it is very hard to rely on the isodensity boundary for the estimation of the charge transfer. In order to avoid any ambiguity in the definition of the z-axis, we recall an approach that may be useful for evaluating the charge transferred between the [^{t}Bu$_3$PAuX(^{Si}NON')] and [CO_2] fragments at TSI.[9]

Within this approach, the electron density rearrangement ($\Delta\rho'$), which typically shows charge accumulation regions (positive values) and charge depletion regions (negative values), defines two different positive functions, $\Delta\rho^+$ and $\Delta\rho^-$, each equal to the magnitude of the appropriate portion, *i.e.*:

$$\Delta\rho^{+/-}(r) = max[\pm\Delta\rho(r)', 0] \qquad [S3]$$

so that

$$\Delta\rho(r)' = \Delta\rho^+(r) - \Delta\rho^-(r) \qquad [S4]$$

By defining two arbitrary regions that are associated with the interacting fragments, we can evaluate the charge transfer as follows:

$$CT = \int_A \Delta\,\rho(r)'dr = -\int_B \Delta\,\rho(r)'dr \qquad [S5]$$

By combining Eqs. [S4] and [S5], CT can also be expressed as:

$$CT = \int_A \Delta\,\rho^{+}(r)dr - \int_A \Delta\,\rho^{-}(r)dr = -\int_B \Delta\,\rho^{+}(r)dr + \int_B \Delta\,\rho^{-}(r) \quad [S6]$$

Ultimately, this approach can also be expressed in the CD-NOCV framework. By combining Equations [S1] and [S5], we can use to this approach for calculating the charge transfer associated to each NOCV deformation density as follows:

$$CT_k = \int_A \Delta\,\rho_k(r)'dr = -\int_B \Delta\,\rho_k(r)'dr \quad [S7]$$

Despite the spatial regions associated to the two interacting fragments being defined arbitrarily, this approach is particularly suitable for the analysis of the interaction between the [$^{t}Bu_3PAuX(^{Si}NON')$] and [CO_2] fragments in TSI, being the two fragments well-separated in space.

- **Energy Decomposition Analysis and ETS-NOCV approach**

In this work the Energy Decomposition Analysis (EDA)[10,11] has been applied to get additional and complementary insights into the interaction between carbon dioxide and the [$^{t}Bu_3PAuX(^{Si}NON')$] complex in the transition state TSI. With this approach, the interaction energy between the [$^{t}Bu_3PAuX(^{Si}NON')$] and [CO_2] fragments can be decomposed in different contributions as follows:

$$\Delta E_{\text{int}} = \Delta E^{Pauli} + \Delta V_{elst} + \Delta E_{oi} + \Delta E_{disp} \quad [S8]$$

where ΔE^{Pauli} represents the Pauli repulsion interaction between occupied orbitals on the two fragments, ΔV_{elst} is the quasiclassical electrostatic interaction between the unperturbed charge distribution of the fragments at their final positions, ΔE_{disp} takes into account the dispersion contribution and ΔE_{oi} is the orbital interaction, which arises from the orbital relaxation and the orbital mixing between the fragments, and accounts for electron pair bonding, charge transfer, and polarization.

The orbital interaction term ΔE_{oi} can be further decomposed within the ETS-NOCV[12] scheme into NOCV pairwise orbital contributions ($\Delta E_{oi} = \sum_k \Delta\, E_{oi}^{k}$) which associates an energy contribution (E_{oi}^{k}) to each NOCV deformation density ($\Delta\rho_k$).

- **Activation Strain Model**

The Activation Strain Model (ASM)[13–15] is a popular approach often used in order to get insights into the factors controlling the activation barrier of a process. Within this framework, the activation barrier ($\Delta E^{\#}$) can be decomposed as follows:

$$\Delta E^{\#} = \left[\Delta E_{\text{dist}}^{TSI} - \Delta E_{dist}^{RC}\right] + \left[\Delta E_{\text{int}}^{TSI} - \Delta E_{\text{int}}^{RC}\right] = \Delta\Delta E_{dist} + \Delta\Delta E_{\text{int}} \qquad [S9]$$

where the "$\Delta E_{dist}{}^{TSI}$" and "$\Delta E_{dist}{}^{RC}$" terms represent the energy penalty due to the distortion of the fragments (i.e. [^{t}Bu$_3$PAuX(^{Si}NON')] and CO_2) constrained in the structures of the transition state (TSI) and the reactant complex (RC) respectively, whereas "$\Delta E_{int}{}^{TSI}$" and "$\Delta E_{int}{}^{RC}$" represent the interaction energies between the fragments (with the geometries constrained at the ones assumed in the TSI and RC, respectively) in the two structures. These terms can be grouped in the "$\Delta\Delta E_{dist}$" and "$\Delta\Delta E_{int}$" terms, that represent the overall distortion and interaction contributions to the activation barrier, respectively.

Additionally, we can also rearrange Equation [S9] in order to express the distortion contributions relatively to the two fragments as follows:

$$\begin{gathered} \Delta\Delta E_{\text{dist}} = E_{CO2}^{TS} - E_{CO2}^{RC} + E_{AuX}^{TS} - E_{AuX}^{RC} \\ \Delta\Delta E_{\text{dist}} = \Delta E_{\text{dist}}^{CO2} + \Delta E_{\text{dist}}^{AuX} \end{gathered} \qquad [S10]$$

where "$\Delta E_{dist}{}^{CO2}$" represent the distortion penalty (or stabilization) due to CO_2 rearranging from its structure in the RC going into TSI and the "$\Delta E_{dist}{}^{AuX}$" term represents the same distortion contribution concerning the rearrangement of the [^{t}Bu$_3$PAuX(^{Si}NON')] complexes.

Since, for the sake of comparison, we analysed in the detail the electronic structure of intermediates INT, in order to exploit the factors controlling the different degrees of stabilization, we extended the ASM to the latters as follows:

$$\Delta E^{\#} = \left[\Delta E_{\text{dist}}^{\text{INT}} - \Delta E_{dist}^{TSI}\right] + \left[\Delta E_{\text{int}}^{\text{INT}} - \Delta E_{\text{int}}^{TSI}\right] = \Delta\Delta E_{dist} + \Delta\Delta E_{\text{int}} \qquad [S11]$$

$$\begin{gathered} \Delta\Delta E_{\text{dist}} = E_{CO2}^{\text{INT}} - E_{CO2}^{TSI} + E_{AuX}^{\text{INT}} - E_{AuX}^{\text{TSI}} \\ \Delta\Delta E_{\text{dist}} = \Delta E_{\text{dist}}^{CO2} + \Delta E_{\text{dist}}^{AuX} \end{gathered} \qquad [S12]$$

where the "$\Delta E_{dist}{}^{INT}$" term represents the energy penalty due to the distortion of the fragments (i.e. [^{t}Bu$_3$PAuX(^{Si}NON')] and CO_2) constrained in the structures of the intermediate (INT), whereas "$\Delta E_{int}{}^{INT}$" represents the interaction energy between the fragments (with the geometries constrained

at the ones assumed in the INT).

- **Conceptual DFT descriptors**

Conceptual DFT[16,17] provides a framework in which, based on DFT calculations, a series of indexes can be defined to quantify the most used and important concepts concerning chemical reactivity, such as electrophilicity and nucleophilicity.

In particular, global reactivity descriptors (GDRs) describe the reactivity of the molecule or molecular fragment as a whole, without explicitly focusing on electrophilic and nucleophilic moieties of the molecule.

In this work we use the reactivity descriptors described in detail in Ref. [18], since these are the ones implemented in the ADF[19] code and all the global descriptors are here defined as follows:

$$\mu = E_e(N+1) - E_e(N-1) \qquad \text{[S13]}$$

$$\mu^{+} = E_e(N-1) - E_e(N) \qquad \text{[S14]}$$

$$\mu^{-} = E_e(N) - E_e(N-1) \qquad \text{[S15]}$$

$$\eta = E_e(N+1) - 2E_e(N) - E_e(N-1) \qquad \text{[S16]}$$

$$\omega = \frac{\mu^2}{2\eta} \qquad \text{[S17]}$$

with *$E_e(N)$*, *$E_e(N+1)$* and *$E_e(N-1)$* being the energies of the neutral, anionic and cationic system, respectively, μ being the electronic chemical potential, η the molecular hardness and ω the electrophilicity index.

A nucleophilicity index N can be also defined as the inverse of the electron donating power ω^{-} (for convention multiplied by 10)[20,21], which, by applying the definitions from Eqs. [S14] and [S15], translates as:

$$N = \frac{10}{\omega^{-}} = \frac{160(\mu^{+} - \mu^{-})}{(3\mu^{-} + \mu^{+})^2} \qquad \text{[S18]}$$

For the investigation of nucleophilic and electrophilic specific sites, one should resort to Local Reactivity Descriptors (GLRs). These descriptors are based on the Fukui functions,[22,23] which are defined as the partial derivatives of the electron density with respect to the number of electrons at a constant external potential and can be expressed as:

$$f(r) = \left(\frac{\delta\rho(r)}{\delta N}\right)_{v(r)} \quad [S19]$$

which can be defined for nucleophilic and electrophilic attacks. By applying the finite difference approximation to Eq. [S19], one can define the functions for nucleophilic and electrophilic attacks as follows:

$$f^{+}(r) = \left(\frac{\delta\rho(r)}{\delta N}\right)^{+}_{v(r)} \approx \rho_{N+1}(r) - \rho_{N}(r)(fornucleophilicattack) \quad [S20]$$

$$f^{-}(r) = \left(\frac{\delta\rho(r)}{\delta N}\right)^{-}_{v(r)} \approx \rho_{N}(r) - \rho_{N-1}(r)(forelectrophilicattack) \quad [S21]$$

with $\rho_N(r)$, $\rho_{N+1}(r)$ and $\rho_{N-1}(r)$ representing the electron densities of the system with N, N+1 and N-1 electrons, respectively. From here, a dual electrophilicity and nucleophilicity descriptor has been defined by Morel *et al.* as follows:[24]

$$\Delta f(r) = [f^{+}(r) - f^{-}(r)] \quad [S22]$$

with $\Delta f(r)$ allowing the simultaneous visualization and evaluation of the electrophilic and nucleophilic sites in a molecule.

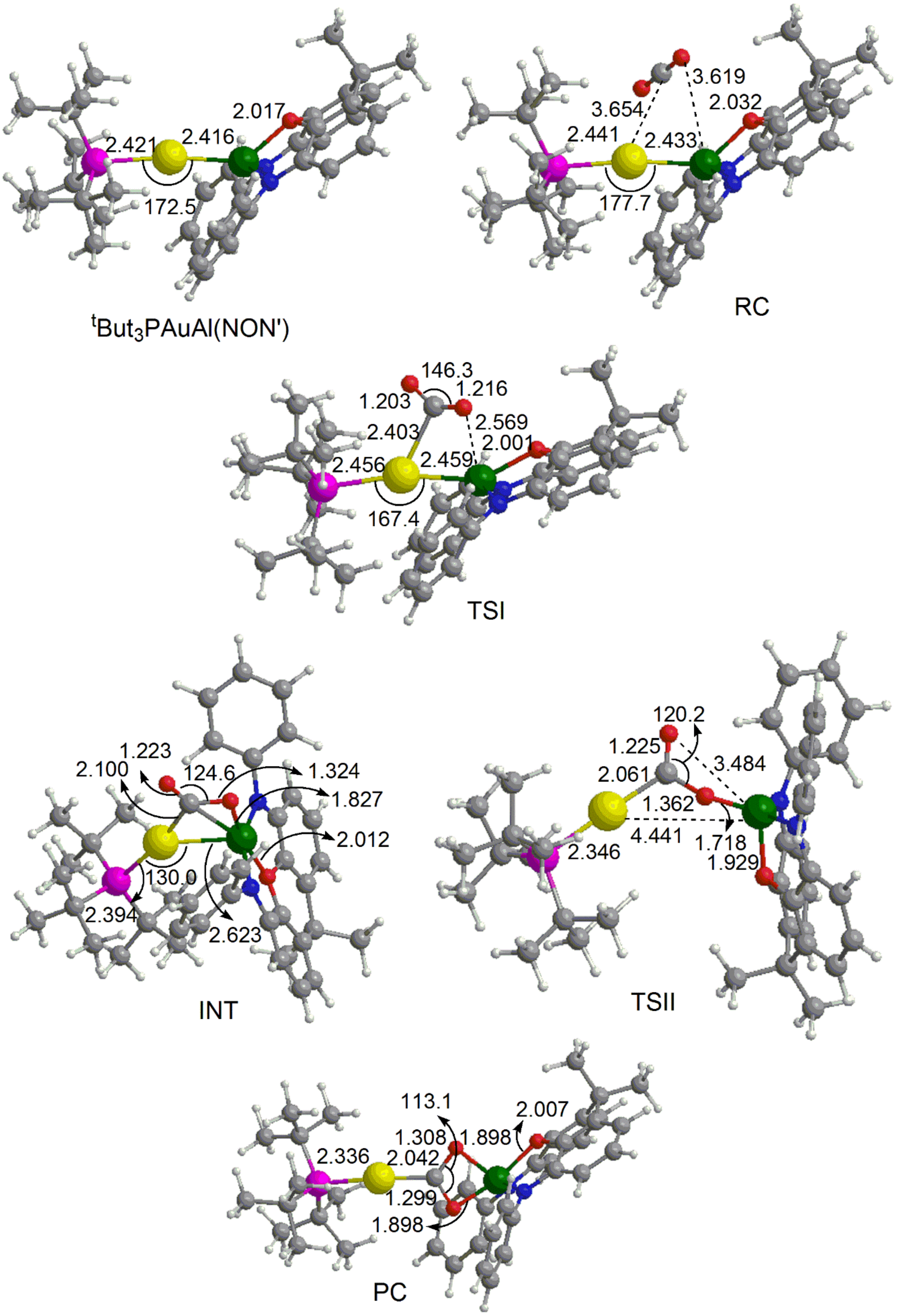

Figure S1. Optimized structures of [$^{t}Bu_3PAuAl(NON')$] and corresponding RC, TSI, INT, TSII and PC complexes. Main geometrical parameters are reported (bond in Å, angles in degree).

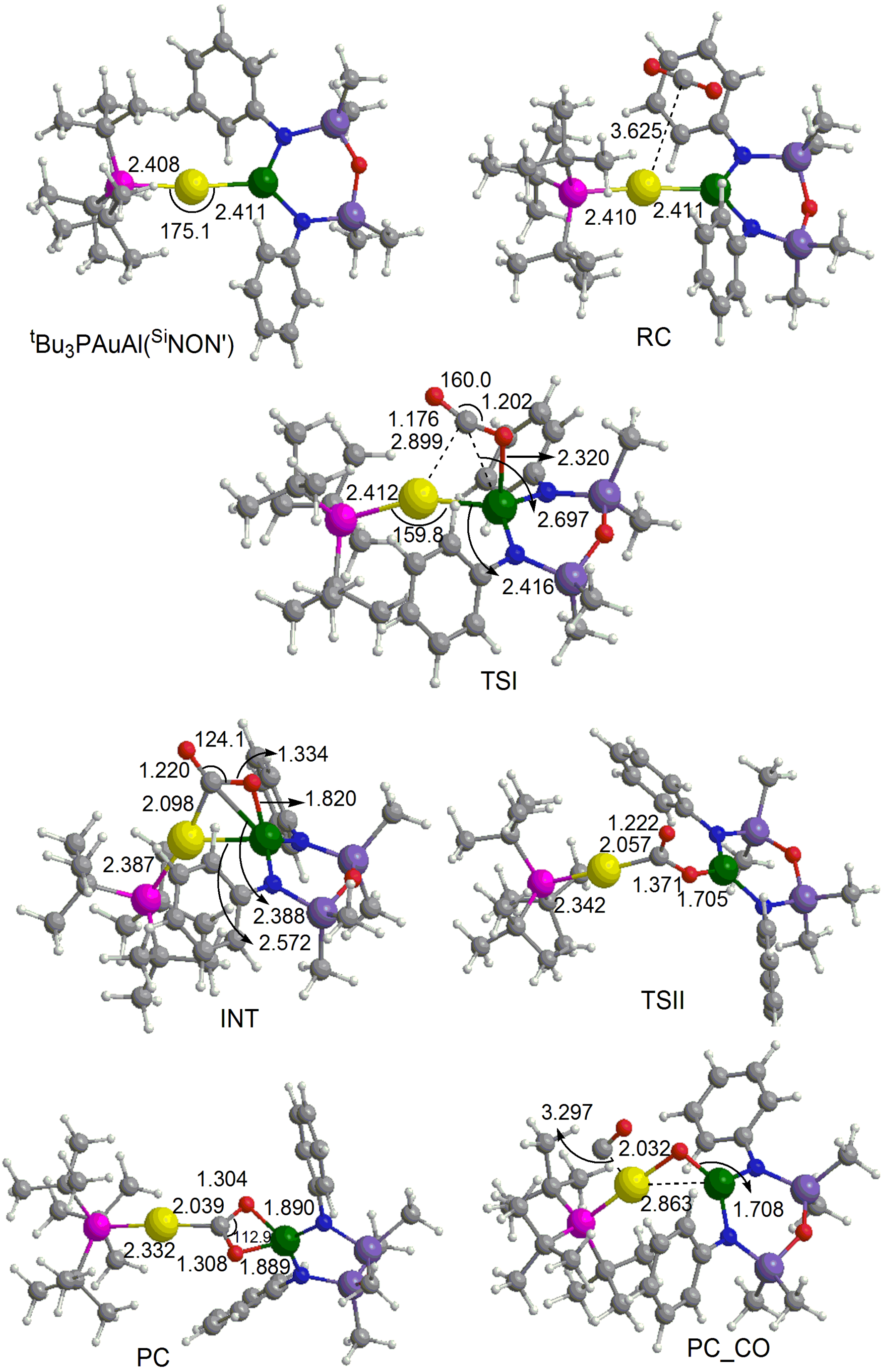

Figure S2. Optimized structures of [^{t}Bu$_3$PAuAl(^{Si}NON')], RC, TSI, INT, TSII, PC and PC_CO complexes. Main geometrical parameters are reported (bond in Å, angles in degree).

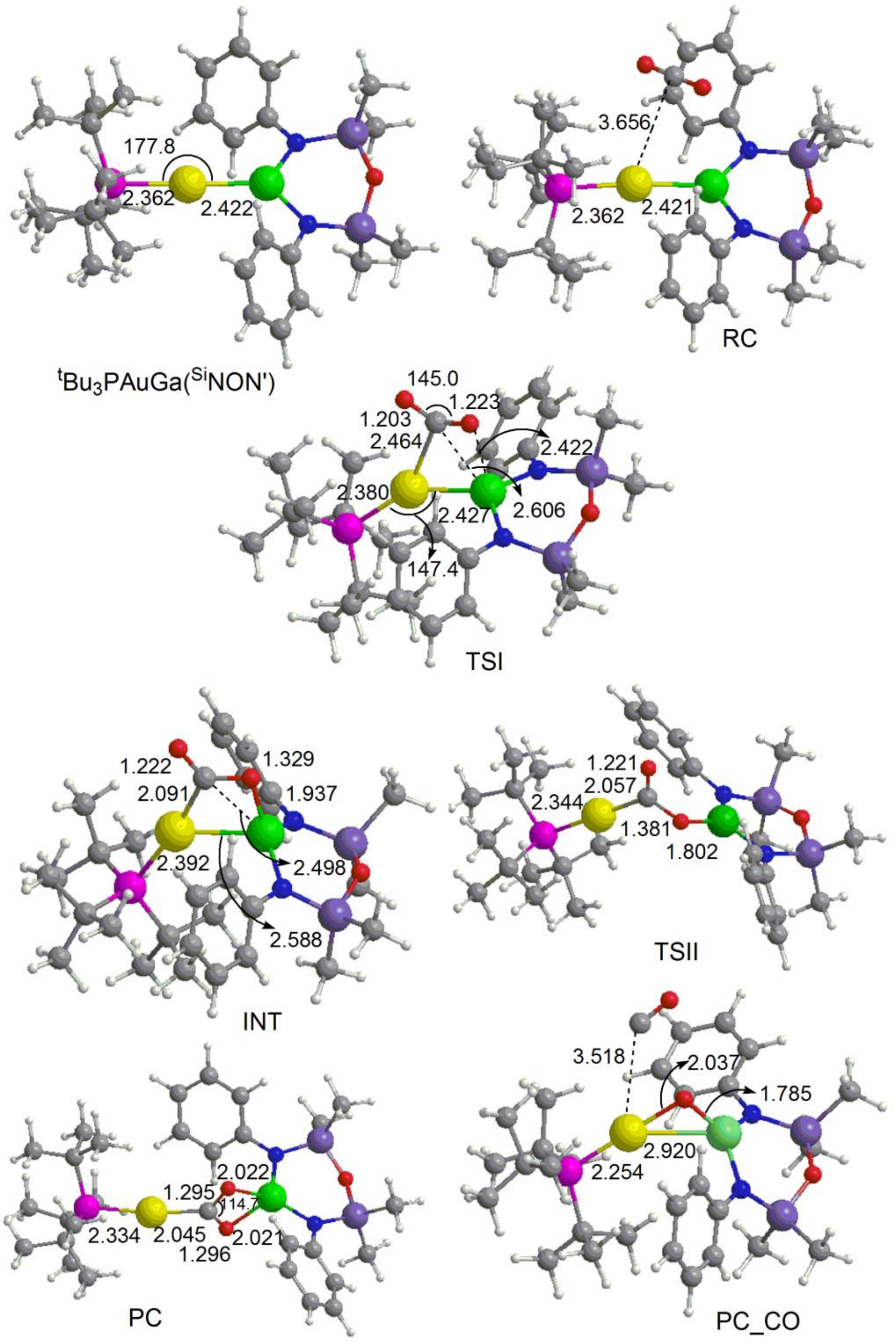

Figure S3. Optimized structures of [^{t}Bu$_3$PAuGa(^{Si}NON')], RC, TSI, INT, TSII, PC and PC_CO complexes. Main geometrical parameters are reported (bond in Å, angles in degree).

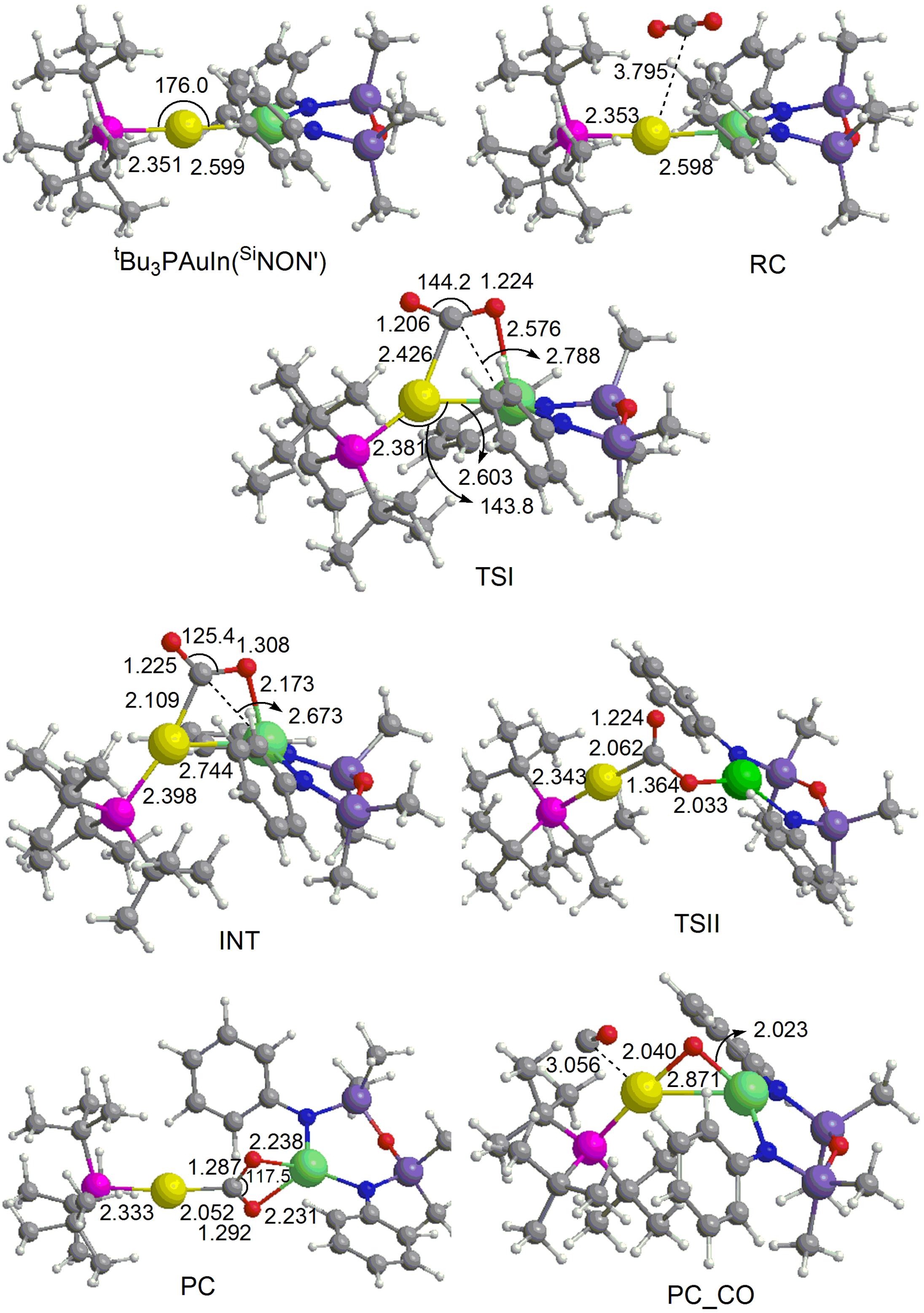

Figure S4. Optimized structures of [^{t}Bu$_3$PAuIn(^{Si}NON')], RC, TSI, INT, TSII, PC and PC_CO complexes. Main geometrical parameters are reported (bond in Å, angles in degree).

	**Al**	**Ga**	**In**
**TSI^X**	-223.6	-172.2	-157.5
**$TSII^X$**	-21.1	-69.1	-35.7

Table S1 Imaginary frequency (in cm^{-1}) corresponding to the concerted transition states **TSI^X** and **$TSII^X$** (X=Al, Ga, In).

For all the **TSI^x** the corresponding imaginary frequency, upon visualization of the related motions, suggests the presence of a concerted transition state involving the Au-CO_2 and X-CO_2 interactions associated to a vibrational motion that involves Au and X and the C and O atoms of CO_2. The smaller imaginary frequencies of **$TSII^X$** is related instead to a mode involving the bending of the two [$^tBu_3PAu$] and [X(^{Si}NON')] fragments between which the CO_2 insertion occurs.

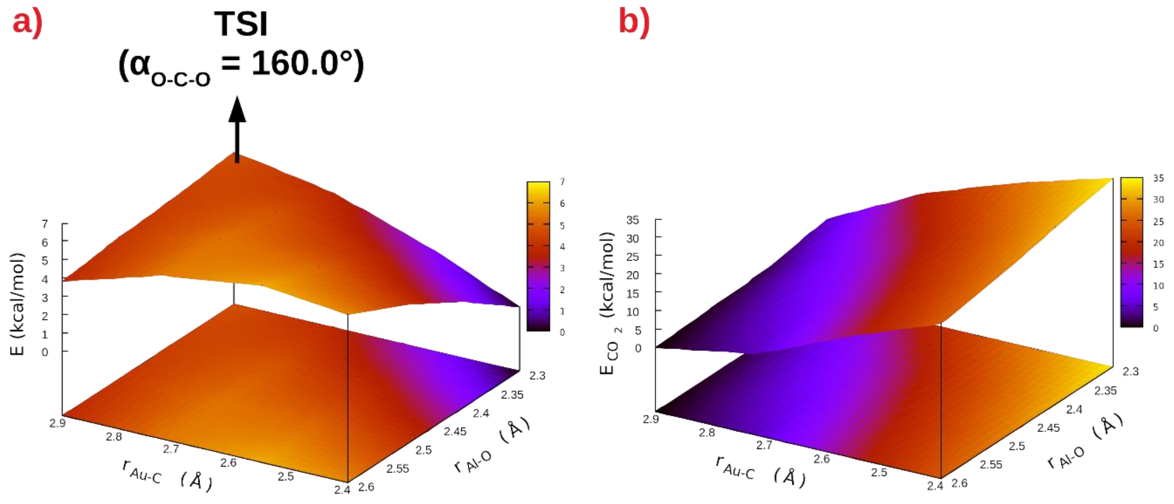

Figure S5. *a)* Potential energy surface (PES) in the region neighbouring **TSI**[Al]. Energy has been shifted in each case according to the minimum energy structure. *b)* Relative energy of the in-adduct geometry of CO_2 for each structure sampled in the PES. In each case, energy has been shifted according to the minimum energy structure.

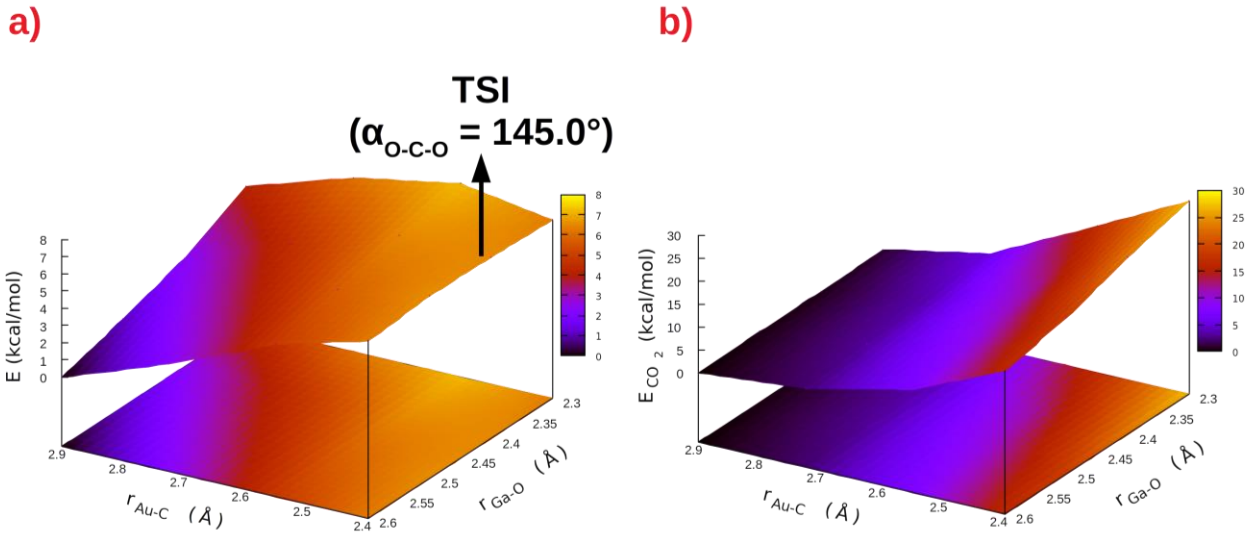

Figure S6. *a)* Potential energy surface (PES) in the region neighbouring **TSI^{Ga}**. Energy has been shifted in each case according to the minimum energy structure. *b)* Relative energy of the in-adduct geometry of CO_2 for each structure sampled in the PES. In each case, energy has been shifted according to the minimum energy structure.

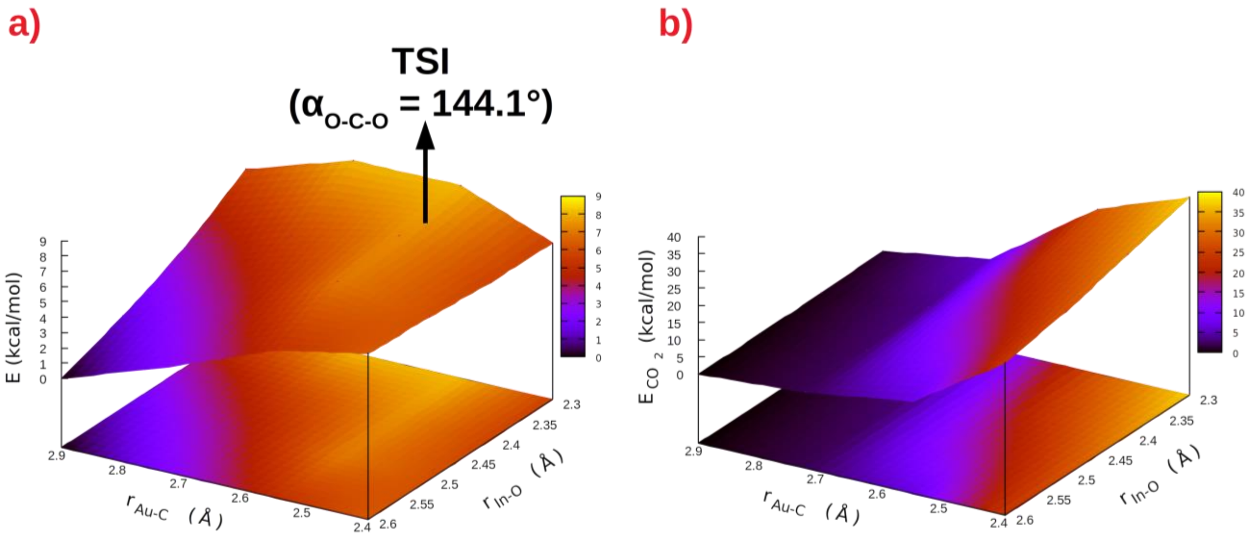

Figure S7. *a)* Potential energy surface (PES) in the region neighbouring **TSI^{In}**. Energy has been shifted in each case according to the minimum energy structure. *b)* Relative energy of the in-adduct geometry of CO_2 for each structure sampled in the PES. In each case, energy has been shifted according to the minimum energy structure.

$r_{Al\text{-}O}$ (Å) $r_{Au\text{-}C}$ (Å)	**2.600**	**2.500**	**2.400**	**2.300**
**2.900**	0.22 *(0.00)*	1.45 *(0.56)*	2.23 *(1.05)*	2.95** *(2.08)*
**2.725**	1.41 *(3.33)*	2.18 *(4.48)*	2.83 *(6.62)*	2.90 *(9.94)*
**2.550**	2.30 *(10.26)*	2.60 *(12.65)*	2.55 *(16.49)*	1.63 *(20.59)*
**2.400**	2.47* *(18.18)*	2.46 *(21.09)*	1.76 *(24.92)*	0.00 *(29.11)*

Table S2. Energies of partially optimized structure associated to the scan of the Potential Energy Surface (PES) along the Au-C and Al-O coordinates around **TSI**. In parenthesis the relative energy of CO_2 in its in-adduct geometry for each point is reported. Energies are given in kcal/mol. In each case, energy has been shifted according to the minimum energy structure.
*closest structure to **TSI**
closest structure to **TSI'

$r_{Al\text{-}O}$ (Å) $r_{Au\text{-}C}$ (Å)	**2.600**	**2.500**	**2.400**	**2.300**
**2.900**	3.82 *(0.00)*	4.20 *(0.84)*	4.41 *(1.74)*	4.78* *(4.61)*
**2.725**	5.43 *(4.69)*	5.37 *(7.90)*	4.83 *(11.99)*	3.83 *(18.11)*
**2.550**	6.13 *(16.16)*	5.13 *(18.96)*	3.72 *(22.40)*	2.00 *(27.29)*
**2.400**	5.66 *(24.72)*	4.18 *(27.31)*	2.31 *(30.68)*	0.00 *(33.95)*

Table S3. Energies of partially optimized structure associated to the scan of the Potential Energy Surface (PES) along the Au-C and Al-O coordinates around $\mathbf{TSI^{Al}}$. In parenthesis the relative energy of CO_2 in its in-adduct geometry for each point is reported. Energies are given in kcal/mol. In each case, energy has been shifted according to the minimum energy structure.
*closest structure to $\mathbf{TSI^{Al}}$

$r_{Ga\text{-}O}$ (Å) $r_{Au\text{-}C}$ (Å)	2.600	2.500	2.400	2.300
**2.900**	0.00 *(0.00)*	1.09 *(0.28)*	2.28 *(0.40)*	4.11 *(0.82)*
**2.725**	2.24 *(2.14)*	3.44 *(3.08)*	4.54 *(3.73)*	6.07 *(5.38)*
**2.550**	4.86 *(7.40)*	5.71 *(9.46)*	6.49 *(11.94)*	7.19 *(16.80)*
**2.400**	6.26 *(16.19)*	6.57 *(18.84)*	6.50* *(23.39)*	6.34 *(26.96)*

Table S4 Energies of partially optimized structure associated to the scan of the Potential Energy Surface (PES) along the Au-C and Ga-O coordinates around **TSI^{Ga}**. In parenthesis the relative energy of CO_2 in its in-adduct geometry for each point is reported. Energies are given in kcal/mol. In each case, energy has been shifted according to the minimum energy structure.
*closest structure to **TSI^{Ga}**

$r_{In\text{-}O}$ (Å) $r_{Au\text{-}C}$ (Å)	2.600	2.500	2.400	2.300
**2.900**	0.00 *(0.00)*	1.45 *(0.14)*	3.30 *(0.47)*	5.82 *(0.94)*
**2.725**	2.42 *(2.04)*	3.91 *(2.56)*	5.74 *(3.46)*	8.03 *(5.84)*
**2.550**	5.13 *(6.88)*	6.97 *(15.24)*	7.38* *(22.27)*	8.06 *(27.67)*
**2.400**	6.30 *(24.05)*	6.10 *(27.55)*	5.77 *(32.50)*	5.72 *(37.35)*

Table S5. Energies of partially optimized structure associated to the scan of the Potential Energy Surface (PES) along the Au-C and In-O coordinates around **TSI^{In}**. In parenthesis the relative energy of CO_2 in its in-adduct geometry for each point is reported. Energies are given in kcal/mol. In each case, energy has been shifted according to the minimum energy structure.
*closest structure to **TSI^{In}**

	Al	Ga	In
**$\Delta E^{\#}$**	5.6	13.6	15.1
**ΔE_{INT}^{RC}**	-5.8	-5.6	-5.6
**ΔE_{INT}^{TS}**	-8.5	-17.9	-18.0
**$\Delta\Delta E_{INT}$**	-2.7	-12.5	-12.4
**ΔE_{DIST}^{CO2}**	7.1	22.3	23.4
**$\Delta E_{DIST}^{complex}$**	1.2	3.8	4.1
**$\Delta\Delta E_{DIST}$**	8.3	26.1	27.5

Table S6. Results of the Activation Strain Model (ASM) analysis of the [CO_2]-[$^{t}Bu_3PAuX(^{Si}NON')$] (X=Al, Ga, In) interaction for the electronic energy activation barrier connecting **RC^{X}** and **TSI^{X}**.

	Al	Ga	In
**ΔE**	-23.5	-8.9	-4.5
**ΔE_{INT}^{TS}**	-8.5	-17.9	-18.0
**ΔE_{INT}^{INT}**	-106.7	-86.1	-69.5
**$\Delta\Delta E_{INT}$**	-98.2	-68.2	-51.5
**ΔE_{DIST}^{CO2}**	59.7	45.1	37.8
**$\Delta E_{DIST}^{complex}$**	15.0	14.2	9.2
**$\Delta\Delta E_{DIST}$**	74.7	59.3	47.0

Table S7. Results of the Activation Strain Model (ASM) analysis of the [CO_2]-[$^{t}Bu_3PAuX(^{Si}NON')$] (X=Al, Ga, In) interaction for the electronic energy activation barrier connecting **TSI^{X}** and **INT^{X}**.

	TSI^{Al}	TSI^{Ga}	TSI^{In}
ΔE_{Pauli}	65.41	110.09	109.23
ΔE_{Elst}	-42.30	-63.71	-63.16
ΔE_{Steric}	23.11	46.38	46.07
ΔE_{oi}	-28.43	-60.12	-60.59
ΔE_{oi}^1	-18.16	-44.42	-45.41
$\|CT_1\|$	0.14	0.35	0.37
ΔE_{oi}^2	-5.36	-5.80	-4.89
$\|CT_2\|$	0.04	0.06	0.07
ΔE_{disp}	-4.72	-4.84	-4.27
ΔE	-10.04	-18.58	-18.79

Table S8. Results of the EDA, ETS-NOCV and CD-NOCV approaches for the $[CO_2]$-$[^tBu_3PAuX(^{Si}NON')]$ (X=Al, Ga, In) interaction at **TSI^{Al}**, **TSI^{Ga}**, and **TSI^{In}**, respectively. All energies are expressed in kcal/mol.

	INT^{Al}	INT^{Ga}	INT^{In}
ΔE_{Pauli}	406.18	366.11	290.03
ΔE_{Elst}	-234.29	-199.85	-160.13
ΔE_{Steric}	171.89	166.26	129.90
ΔE_{oi}	-272.90	-246.16	-193.80
ΔE_{oi}^1	-225.30	-204.00	-159.02
$\|CT^1\|$	0.67	0.61	0.60
ΔE_{oi}^2	-11.10	-11.90	-9.76
$\|CT^2\|$	0.07	0.07	0.07
ΔE_{disp}	-4.87	-4.89	-4.21
ΔE	-105.88	-84.79	-68.11

Table S9. Results of the EDA, ETS-NOCV and CD-NOCV approaches for the analysis of the $[CO_2]$-$[^tBu_3PAuAl(^{Si}NON')]$, $[CO_2]$-$[^tBu_3PAuGa(^{Si}NON')]$ and $[CO_2]$-$[^tBu_3PAuIn(^{Si}NON')]$ interaction at **INT^{Al}**, **INT^{Ga}**, and **INT^{In}**, respectively. All energies are expressed in kcal/mol.

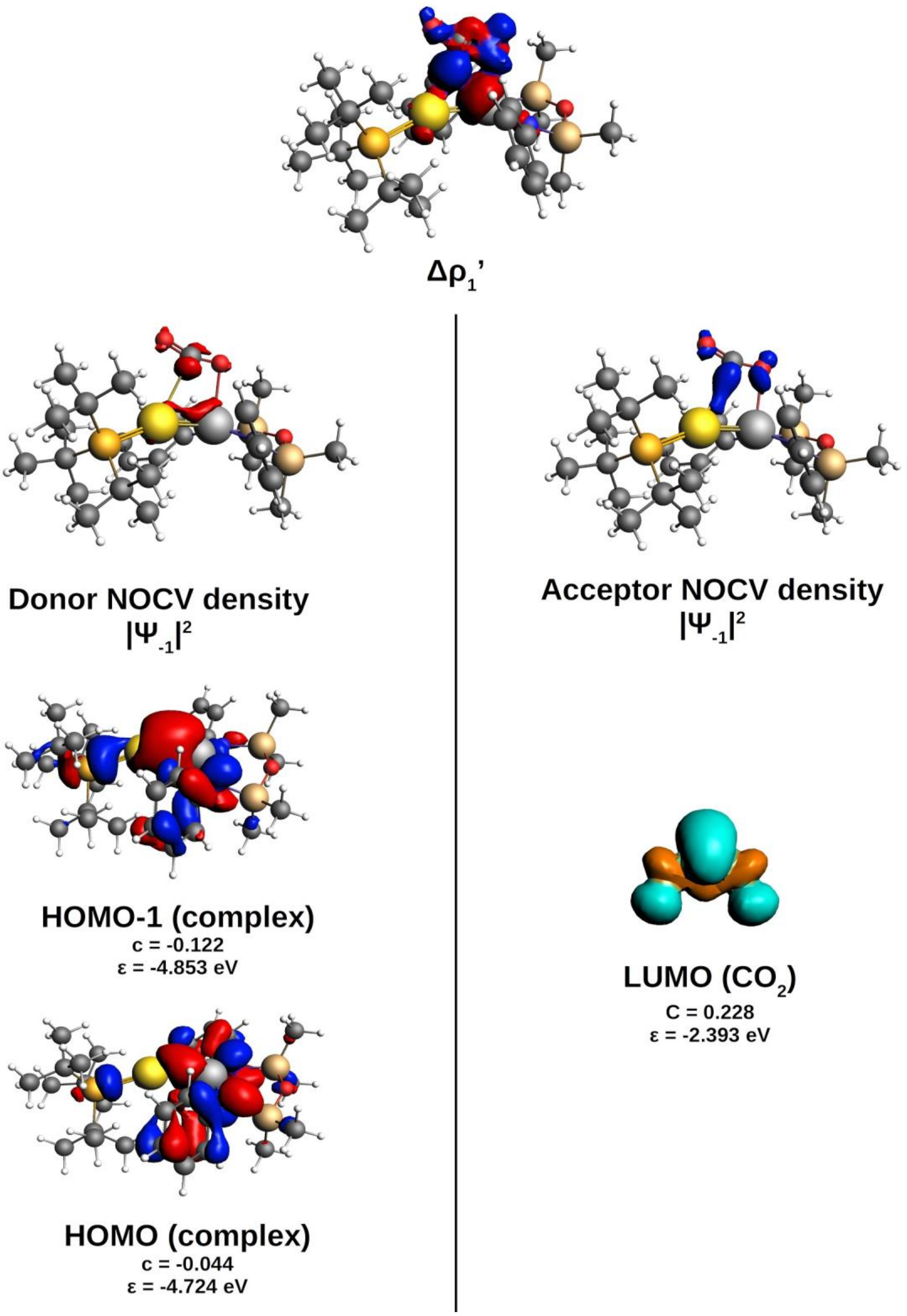

Figure S8. Breakdown of the donor ($|\Psi_1|^2$) and acceptor ($|\Psi_{-1}|^2$) NOCV densities that are associated with the deformation density $\Delta\rho'_1$ in the transition state **TSI^{Al}** into the most important MOs of the fragments frozen at their TS geometry. The mixing coefficients are given in parentheses.

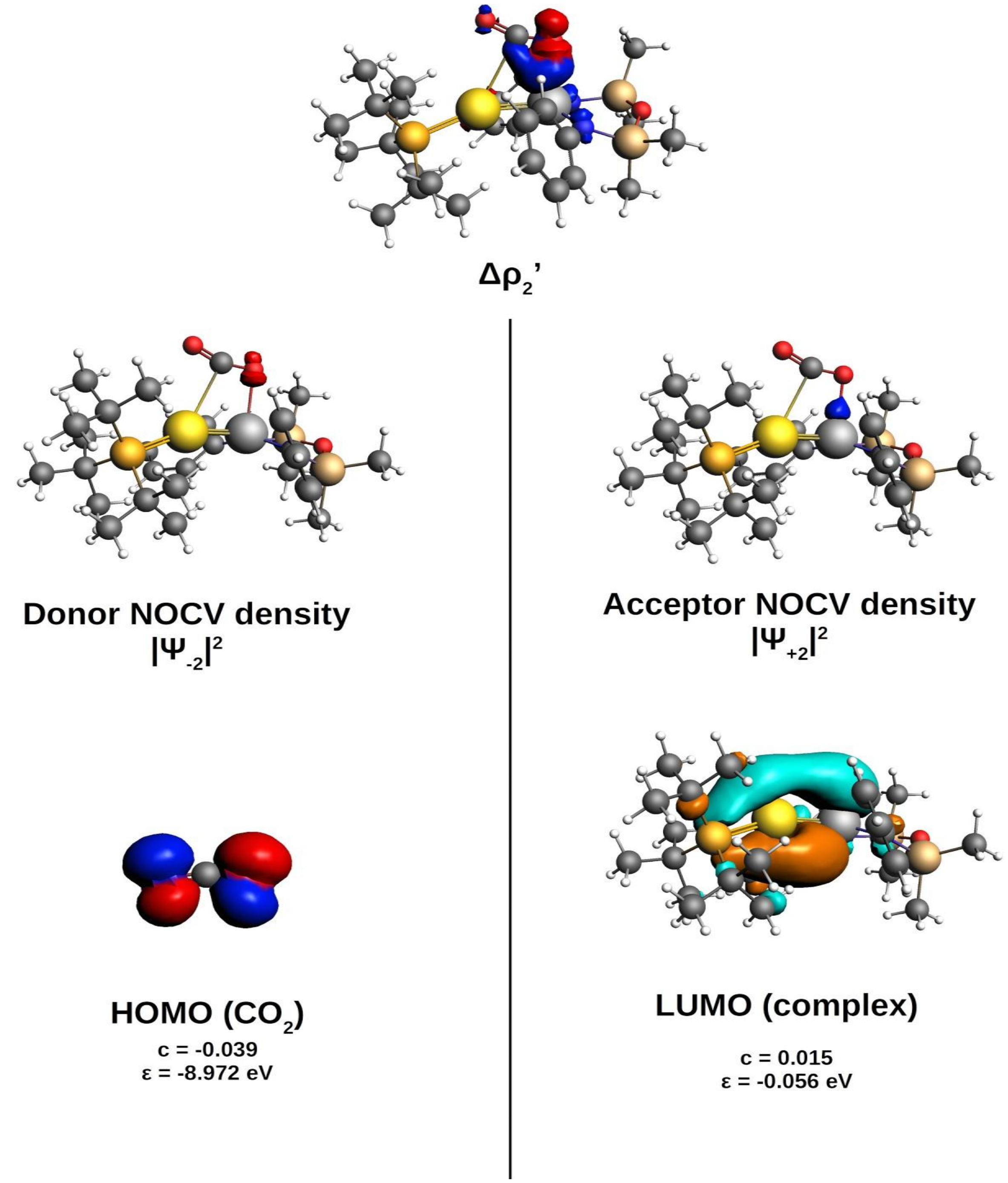

Figure S9. Breakdown of the donor ($|\Psi_2|^2$) and acceptor ($|\Psi_{-2}|^2$) NOCV densities that are associated with the deformation density $\Delta\rho'_2$ in the transition state **TSI$^{\mathbf{Al}}$** into the most important MOs of the fragments frozen at their TS geometry. The mixing coefficients are given in parentheses.

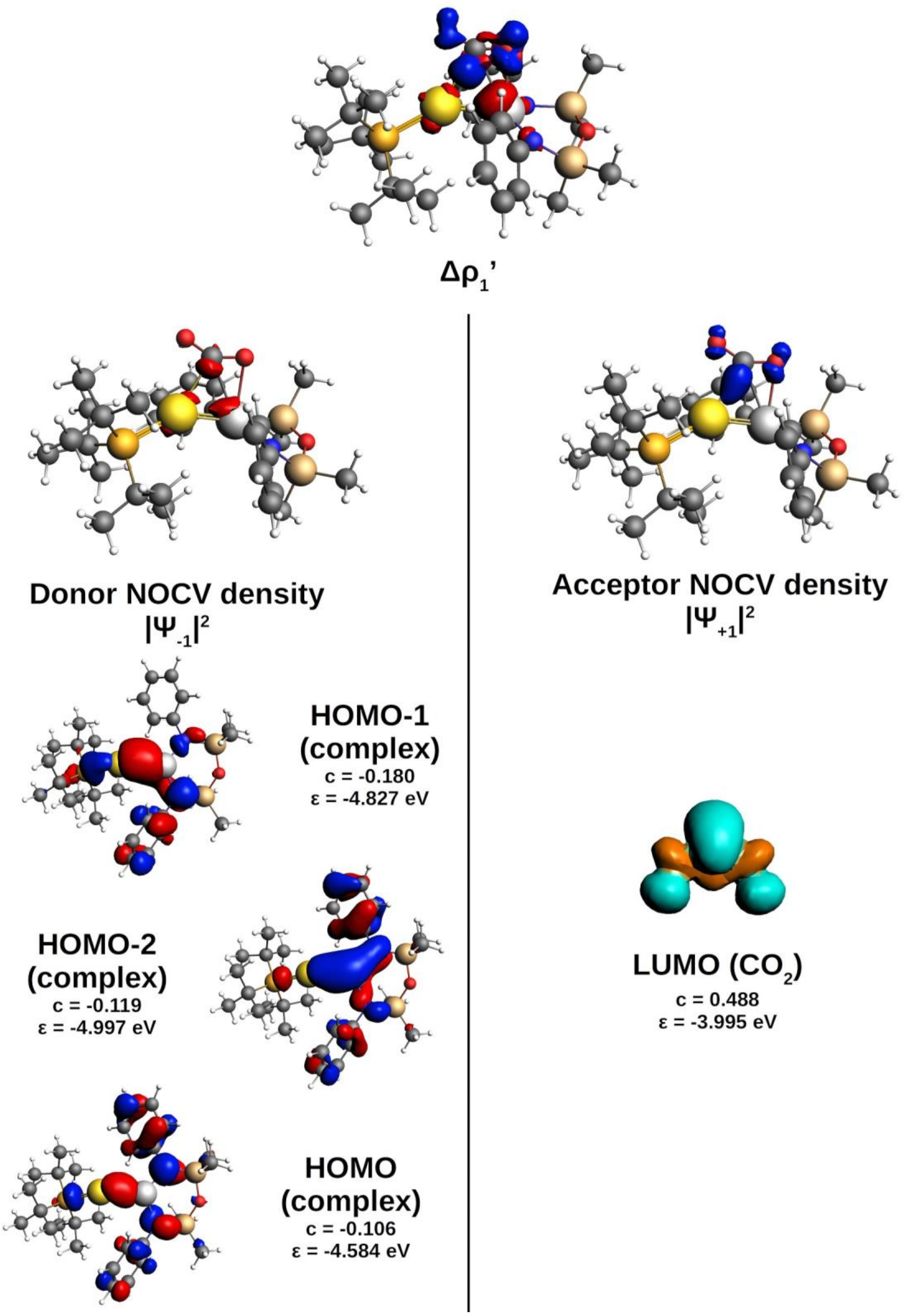

Figure S10. Breakdown of the donor ($|\Psi_1|^2$) and acceptor ($|\Psi_{-1}|^2$) NOCV densitie that are associated with the deformation density $\Delta\rho'_1$ in the transition state **TSI^{Ga}** into the most important MOs of the fragments frozen at their TS geometry. The mixing coefficients are given in parentheses.

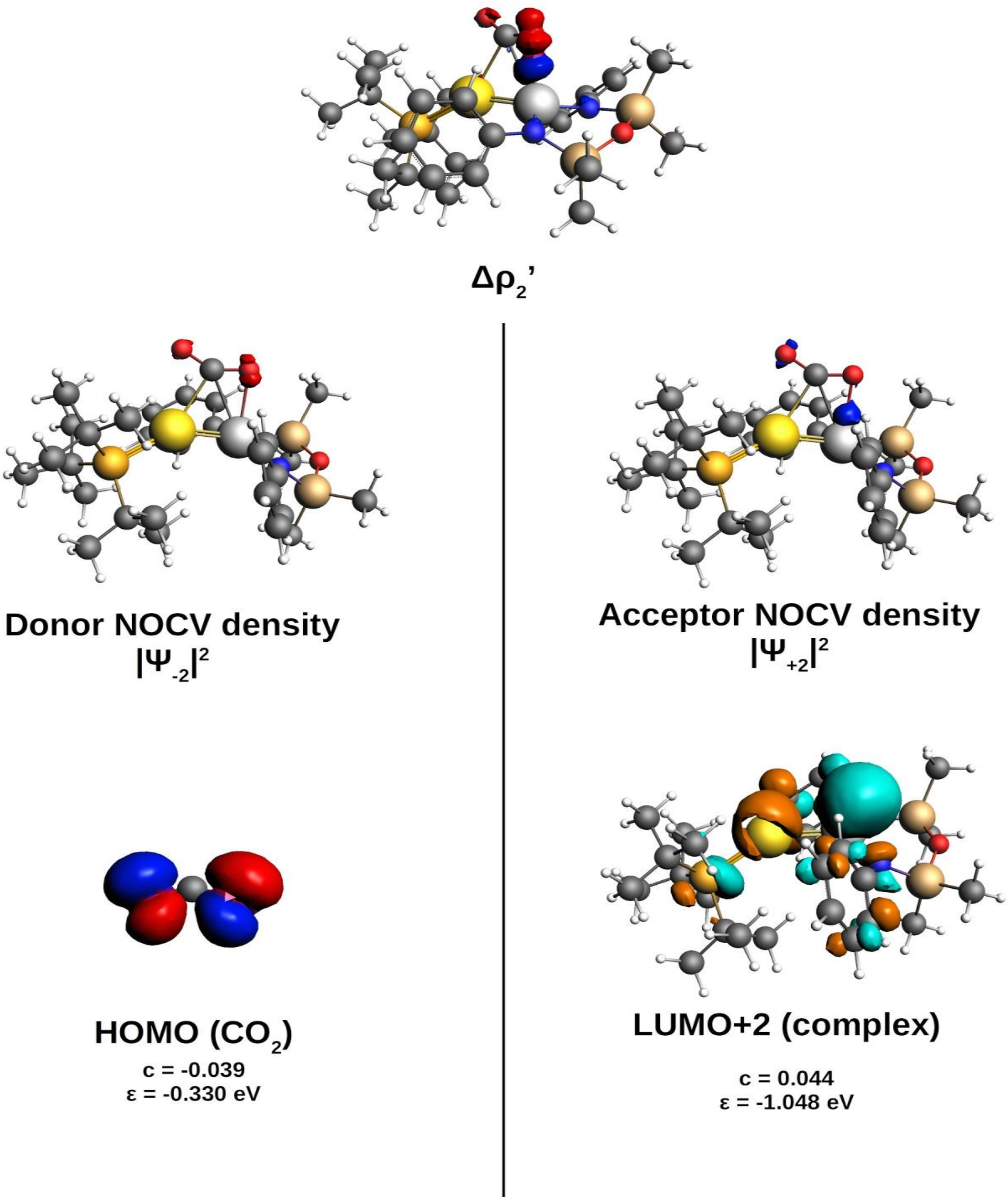

Figure S11. Breakdown of the donor ($|\Psi_2|^2$) and acceptor ($|\Psi_{-2}|^2$) NOCV densities that are associated with the deformation density $\Delta\rho'_2$ in the transition state **TSI^{Ga}** into the most important MOs of the fragments frozen at their TS geometry. The mixing coefficients are given in parentheses.

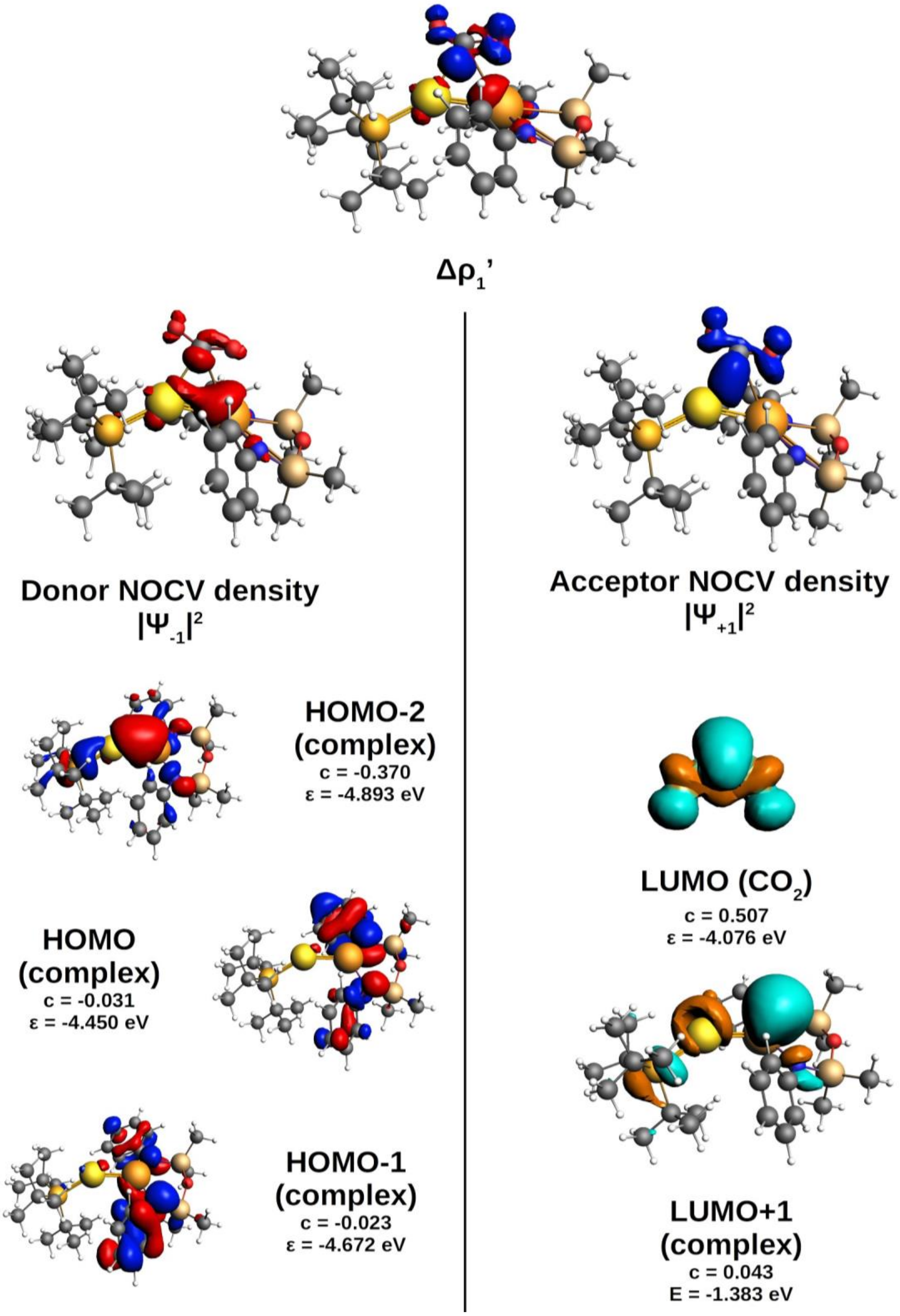

Figure S12. Breakdown of the donor ($|\Psi_1|^2$) and acceptor ($|\Psi_{-1}|^2$) NOCV densitie that are associated with the deformation density $\Delta\rho'_1$ in the transition state **TSI^{In}** into the most important MOs of the fragments frozen at their TS geometry. The mixing coefficients are given in parentheses.

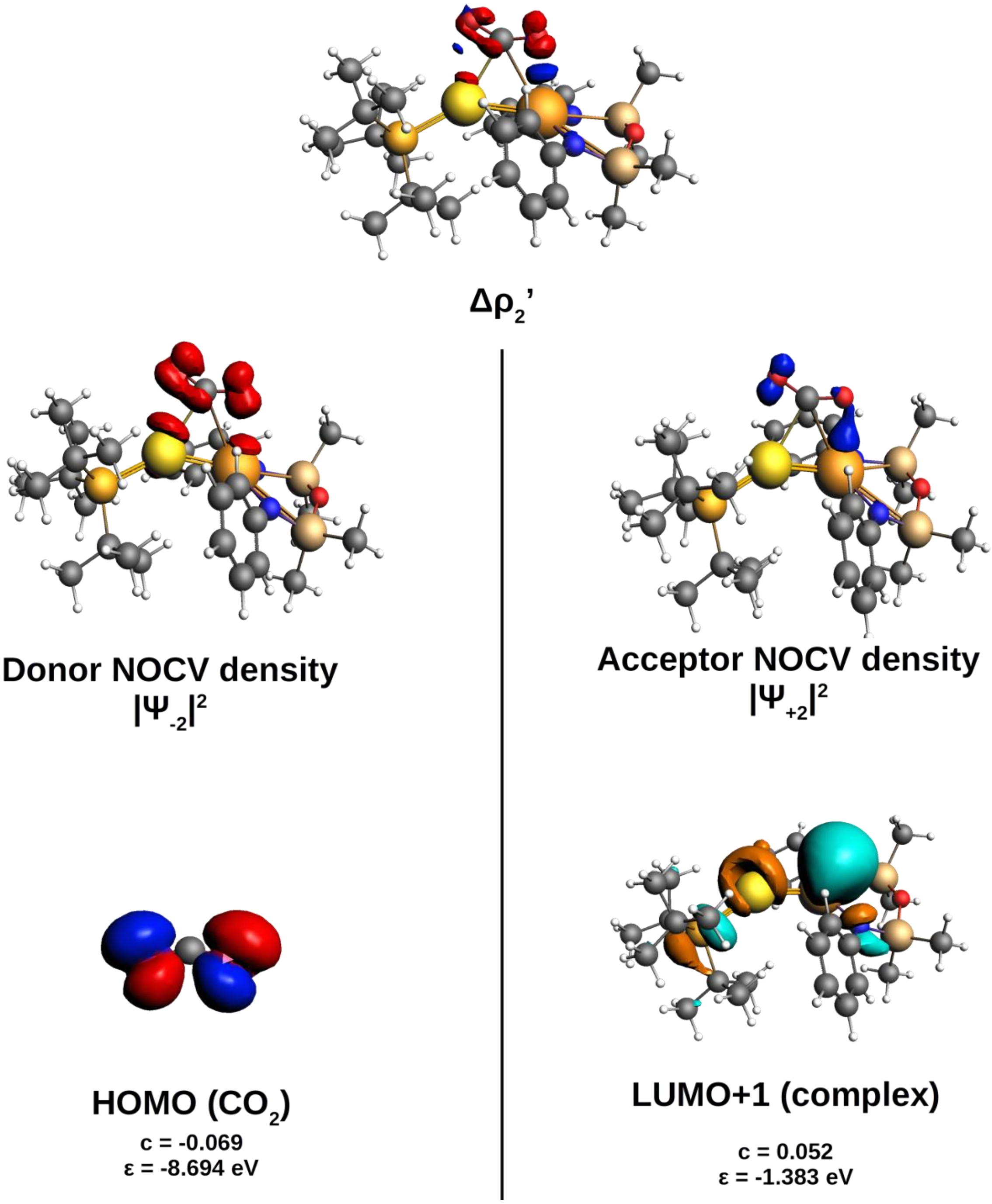

Figure S13. Breakdown of the donor ($|\Psi_2|^2$) and acceptor ($|\Psi_{-2}|^2$) NOCV densitie that are associated with the deformation density $\Delta\rho'_2$ in the transition state **TSI$^{\mathbf{In}}$** into the most important MOs of the fragments frozen at their TS geometry. The mixing coefficients are given in parentheses.

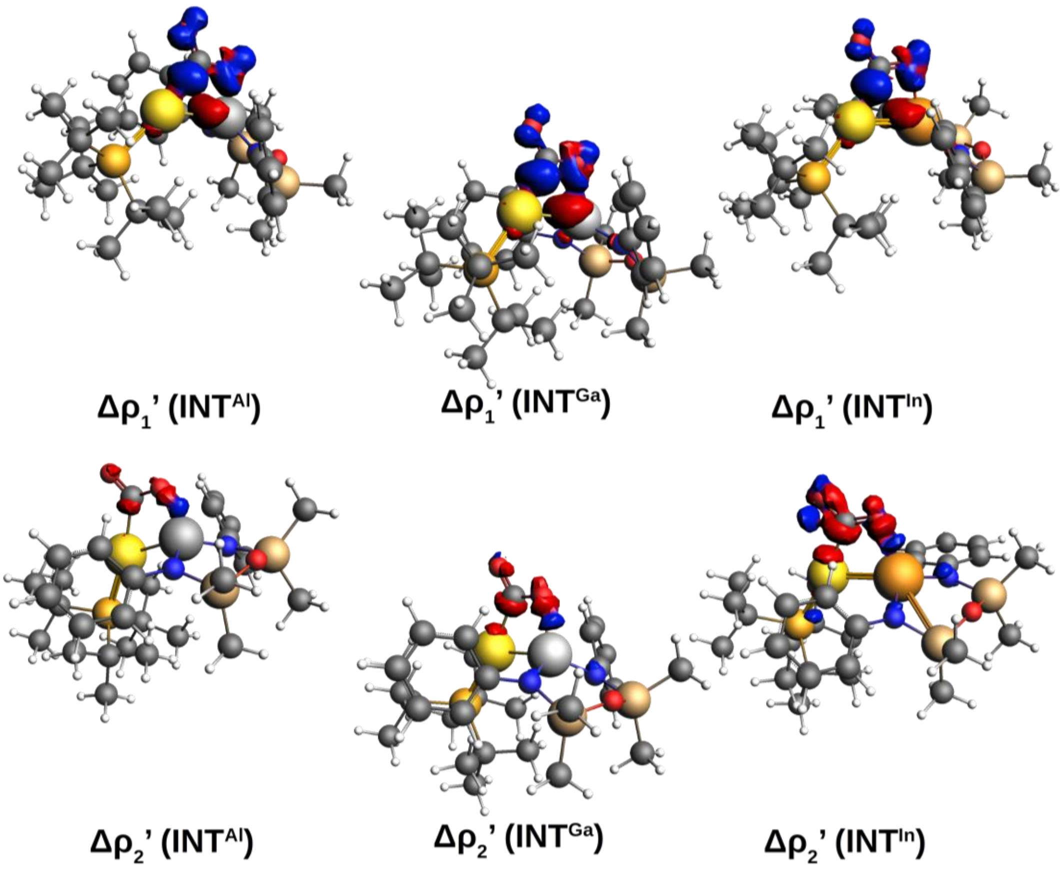

Figure S14. Isodensity surfaces of the main NOCV deformation densities ($\Delta\rho_1$' and $\Delta\rho_2$') for the [CO_2]-[^{t}Bu$_3$PAuAl(^{Si}NON')], [CO_2]-[^{t}Bu$_3$PAuGa(^{Si}NON')] and [CO_2]-[^{t}Bu$_3$PAuIn(^{Si}NON')] interaction in complexes **INT^{Al}**, **INT^{Ga}**, and **INT^{In}**, respectively.

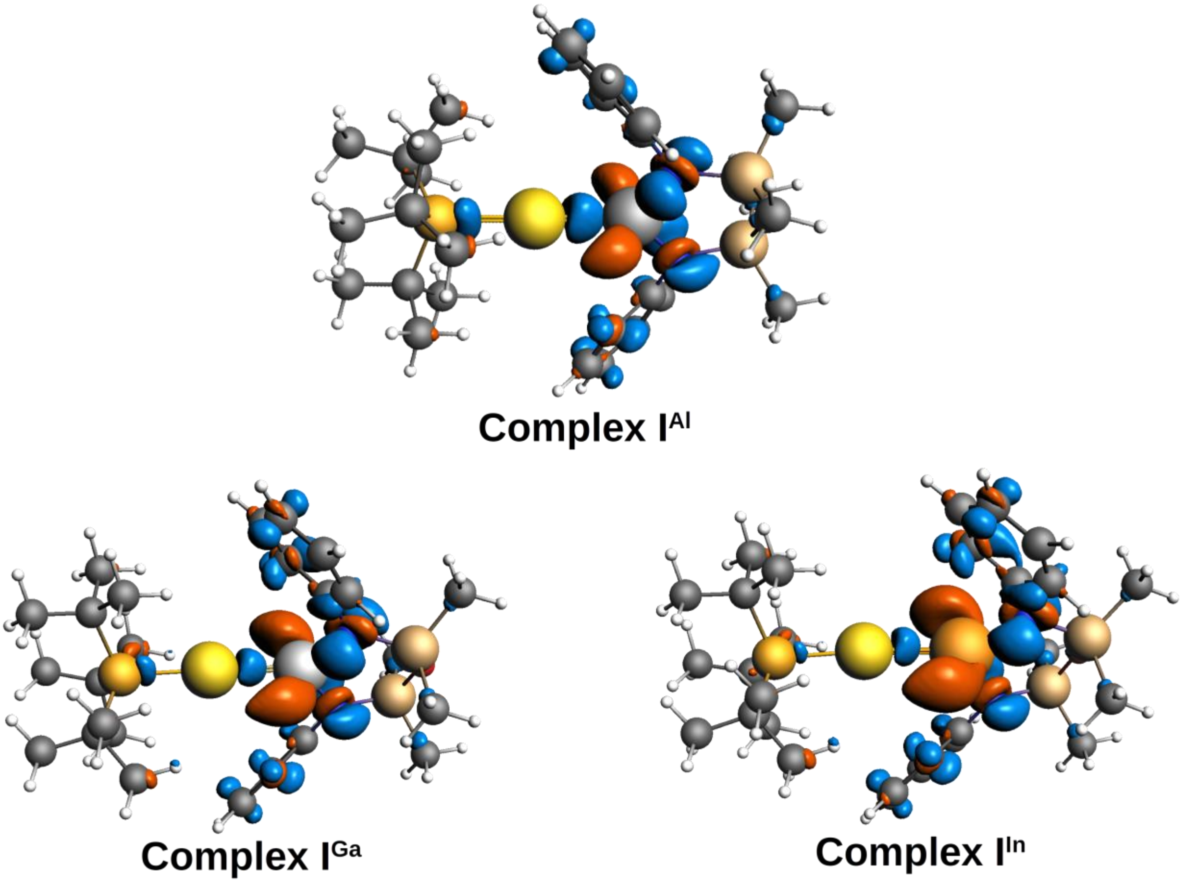

Figure S15. Plots (isovalue 2 me/a_0) of the dual electrophilicity and nucleophilicity descriptor for complexes $\mathbf{I}^{X}$ (X=Al, Ga, In). Orange areas identify electrophilic regions, whereas light blue areas identify nucleophilic regions.

	I^{Al}	I^{Ga}	I^{In}
**Donor MO energy (eV)**	-4.853	-4.827	-4.893
**Donor MO composition**	13.2% 3s Al<br>12.6% $2p_z$ N<br>10.2% $3p_x$ Al<br>7.7% 6s Au<br>7.0% $3p_x$ P<br>6.7% $6p_x$ Au	16.4% $2p_z$ N<br>8.3% 4s Ga<br>7.7% $4p_x$ Ga<br>4.8% $6p_z$ Au<br>4.8% $3p_x$ P	10.3% 5s In<br>8.2% $6p_x$ Au<br>7.7% $3p_x$ P<br>6.7% $5p_x$ In

Table S10. Energies and most relevant atomic orbitals' contributions to the main donor MO of complexes $\mathbf{I^{Al}}$, $\mathbf{I^{Ga}}$ and $\mathbf{I^{In}}$ (HOMO-1 for $\mathbf{I^{Al}}$ and $\mathbf{I^{Ga}}$ and HOMO-2 for $\mathbf{I^{In}}$) identified via NOCV analysis for the transition states $\mathbf{TSI^{X}}$.

	[^{t}Bu$_3$PAu]· - [Al(^{Si}NON')]·	[^{t}Bu$_3$PAu]$^{+}$- [Al(^{Si}NON')]$^{-}$	[^{t}Bu$_3$PAu]$^{-}$- [Al(^{Si}NON')]$^{+}$
**ΔE_{Pauli}**	158.71	221.72	182.70
**ΔE_{Elst}**	-166.04	-285.64	-239.69
**ΔE_{Steric}**	-7.33	-63.92	-56.98
**ΔE_{oi}**	-70.86	-100.62	-176.43
**ΔE_{disp}**	-8.94	-8.94	-8.94
**ΔE**	-87.13	-173.48	-242.35

Table S11. Energy Decomposition Analysis (EDA) of the interaction energy between [^{t}Bu$_3$PAu] and [Al(^{Si}NON')] fragments in complex **I^{Al}** using different fragmentations, i.e. doublet open shell neutral fragments (first column), singlet [^{t}Bu$_3$PAu]$^{+}$ and [Al(^{Si}NON')]$^{-}$ fragments (second column) and singlet [^{t}Bu$_3$PAu]$^{-}$ and [Al(^{Si}NON')]$^{+}$ fragments (third column). Energies are reported in kcal/mol.

	[^{t}Bu$_3$PAu]· - [Ga(^{Si}NON')]·	[^{t}Bu$_3$PAu]$^{+}$- [Ga(^{Si}NON')]$^{-}$	[^{t}Bu$_3$PAu]$^{-}$- [Ga(^{Si}NON')]$^{+}$
**ΔE_{Pauli}**	159.01	170.01	232.76
**ΔE_{Elst}**	-155.51	-229.44	-264.12
**ΔE_{Steric}**	3.50	-59.43	-31.36
**ΔE_{oi}**	-81.96	-89.84	-201.50
**ΔE_{disp}**	-8.71	-8.71	-8.71
**ΔE**	-87.16	-157.97	-241.56

Table S12. Energy Decomposition Analysis (EDA) of the interaction energy between [^{t}Bu$_3$PAu] and [Ga(^{Si}NON')] fragments in complex **I^{Ga}** using different fragmentations, i.e. doublet open shell neutral fragments (first column), singlet [^{t}Bu$_3$PAu]$^{+}$ and [Ga(^{Si}NON')]$^{-}$ fragments (second column) and singlet [^{t}Bu$_3$PAu]$^{-}$ and [Ga(^{Si}NON')]$^{+}$ fragments (third column). Energies are reported in kcal/mol.

	$[^tBu_3PAu]\cdot$ - $[In(^{Si}NON')]\cdot$	$[^tBu_3PAu]^+$- $[In(^{Si}NON')]^-$	$[^tBu_3PAu]^-$- $[In(^{Si}NON')]^+$
**ΔE_{Pauli}**	146.68	128.89	230.77
**ΔE_{Elst}**	-141.81	-181.24	-265.56
**ΔE_{Steric}**	4.87	-52.35	-34.79
**ΔE_{oi}**	-76.88	-80.59	-189.61
**ΔE_{disp}**	-7.36	-7.36	-7.36
**ΔE**	-79.37	-140.30	-231.75

Table S13. Energy Decomposition Analysis (EDA) of the interaction energy between $[^tBu_3PAu]$ and $[In(^{Si}NON')]$ fragments in complex $\mathbf{I^{In}}$ using different fragmentations, i.e. doublet open shell neutral fragments (first column), singlet $[^tBu_3PAu]^+$ and $[In(^{Si}NON')]^-$ fragments (second column) and singlet $[^tBu_3PAu]^-$ and $[In(^{Si}NON')]^+$ fragments (third column). Energies are reported in kcal/mol.

Complex I^{Al}						
**Net CT (e)**	**-0.023**					
	**α**			**β**		
**k**	**$\lvert\nu_k\rvert$**	**ΔE_{oi}^k (kcal/mol)**	**CT^k (e)**	**$\lvert\nu_k\rvert$**	**ΔE_{oi}^k (kcal/mol)**	**CT^k (e)**
1	0.44	-33.09	-0.312	0.41	-23.42	0.299
2	0.08	-2.04	-0.019	0.09	-2.23	-0.019
3	0.07	-1.74	-0.013	0.07	-1.85	-0.014
**Complex I^{Ga}**						
**Net CT (e)**	**-0.042**					
	**α**			**β**		
**k**	**$\lvert\nu_k\rvert$**	**ΔE_{oi}^k (kcal/mol)**	**CT^k (e)**	**$\lvert\nu_k\rvert$**	**ΔE_{oi}^k (kcal/mol)**	**CT^k (e)**
1	0.56	-47.43	-0.364	0.39	-21.55	0.286
2	0.08	-1.76	-0.012	0.08	-1.83	-0.018
3	0.06	-1.69	0.008	0.06	-1.26	-0.004
**Complex I^{In}**						
**Net CT (e)**	**-0.152**					
	**α**			**β**		
**k**	**$\lvert\nu_k\rvert$**	**ΔE_{oi}^k (kcal/mol)**	**CT^k (e)**	**$\lvert\nu_k\rvert$**	**ΔE_{oi}^k (kcal/mol)**	**CT^k (e)**
1	0.61	-45.29	-0.420	-0.41	-20.28	0.260
2	0.08	-2.08	0.005	-0.07	-1.37	-0.011
3	0.07	-1.37	-0.010	-0.06	-1.04	-0.007

Table S14. Eigenvalues ($\lvert\nu_k\rvert$), orbital interaction energies (ΔE_{oi}^k) and charge transfer (CT_k) associated to the first three NOCV deformation densities (k=1-3) and to the corresponding α and β components for complexes $\mathbf{I^{Al}}$, $\mathbf{I^{Ga}}$ and $\mathbf{I^{In}}$.

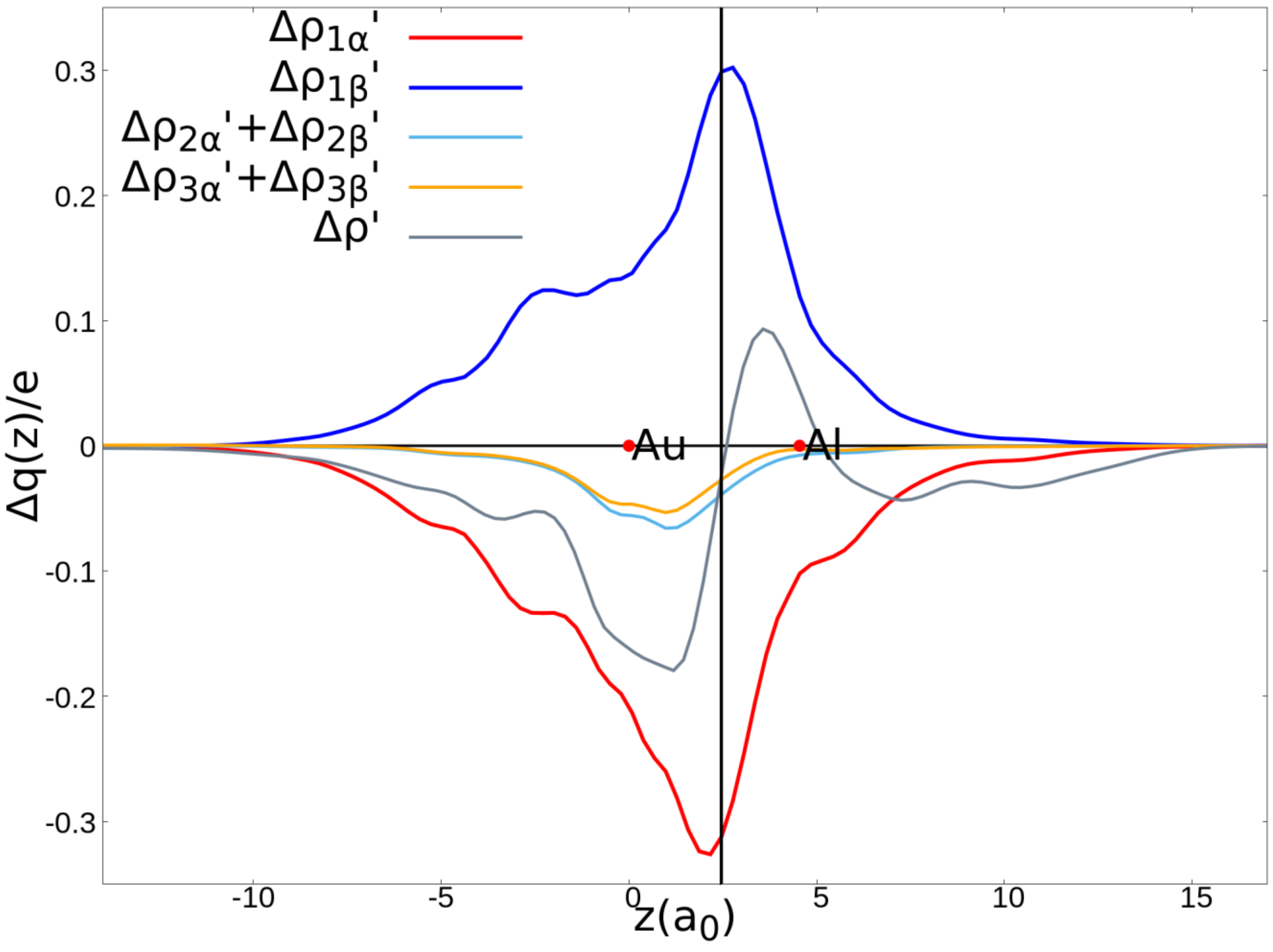

Figure S16. Charge Displacement (CD-NOCV) curves for the interaction between doublet [^{t}Bu$_3$PAu]· and [Al(^{Si}NON')]· fragments in the [^{t}Bu$_3$PAuAl(^{Si}NON')] complex. Red dots indicate the position of the nuclei along the z axis. The vertical solid line marks the isodensity boundary between the fragments. Positive (negative) values of the curve indicate right-to-left (left-to-right) charge transfer.

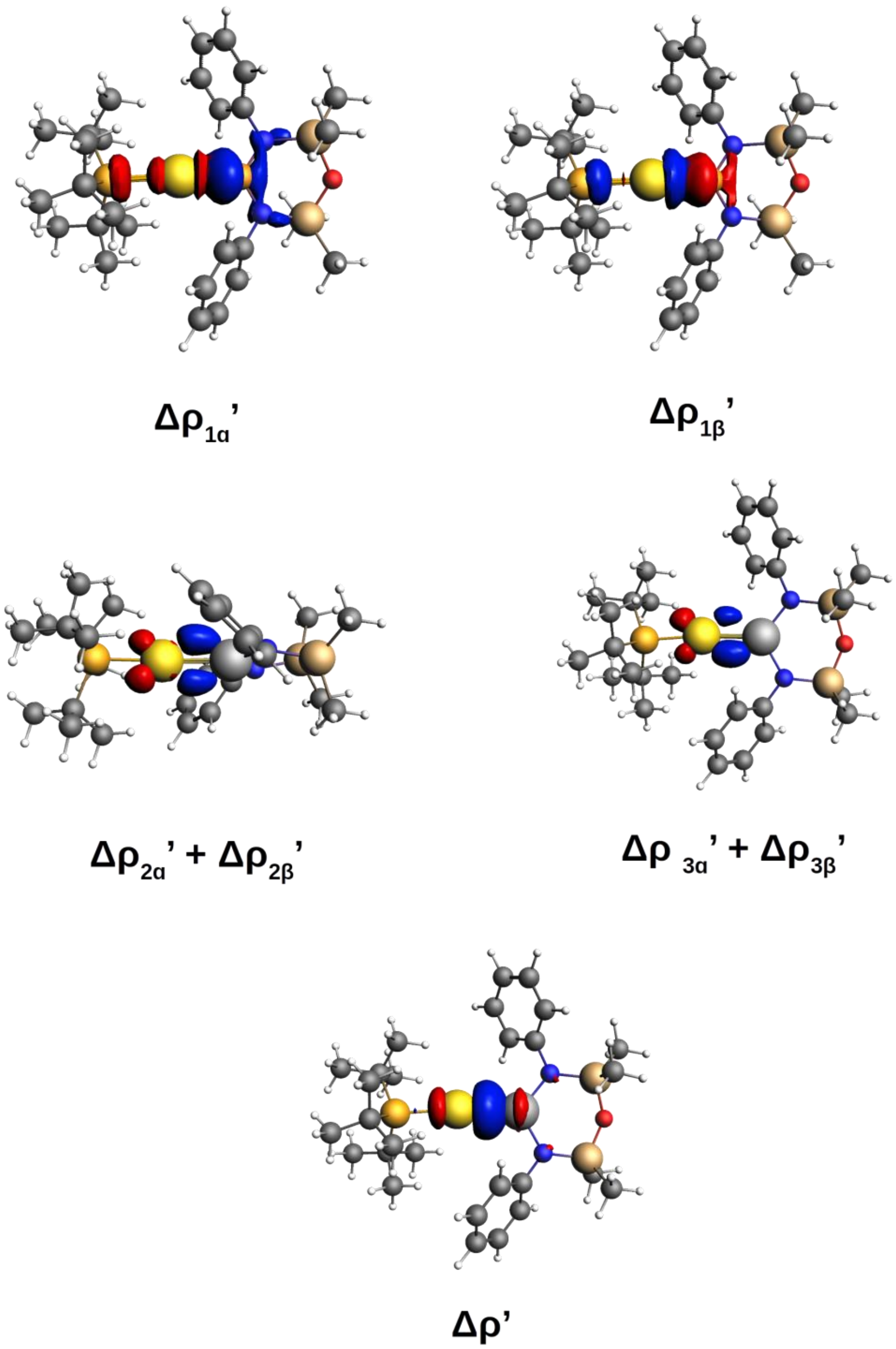

Figure S17. Isodensity surfaces of the $\Delta\rho_{1\alpha}$' and $\Delta\rho_{1\beta}$', $\Delta\rho_2$', $\Delta\rho_3$' and $\Delta\rho$' NOCV deformation densities for the interaction between doublet [^{t}Bu$_3$PAu]· and [Al(^{Si}NON')]· fragments in the [^{t}Bu$_3$PAuAl(^{Si}NON')] complex. Blue regions indicate electron density accumulation areas, whereas red regions indicate depletion areas.

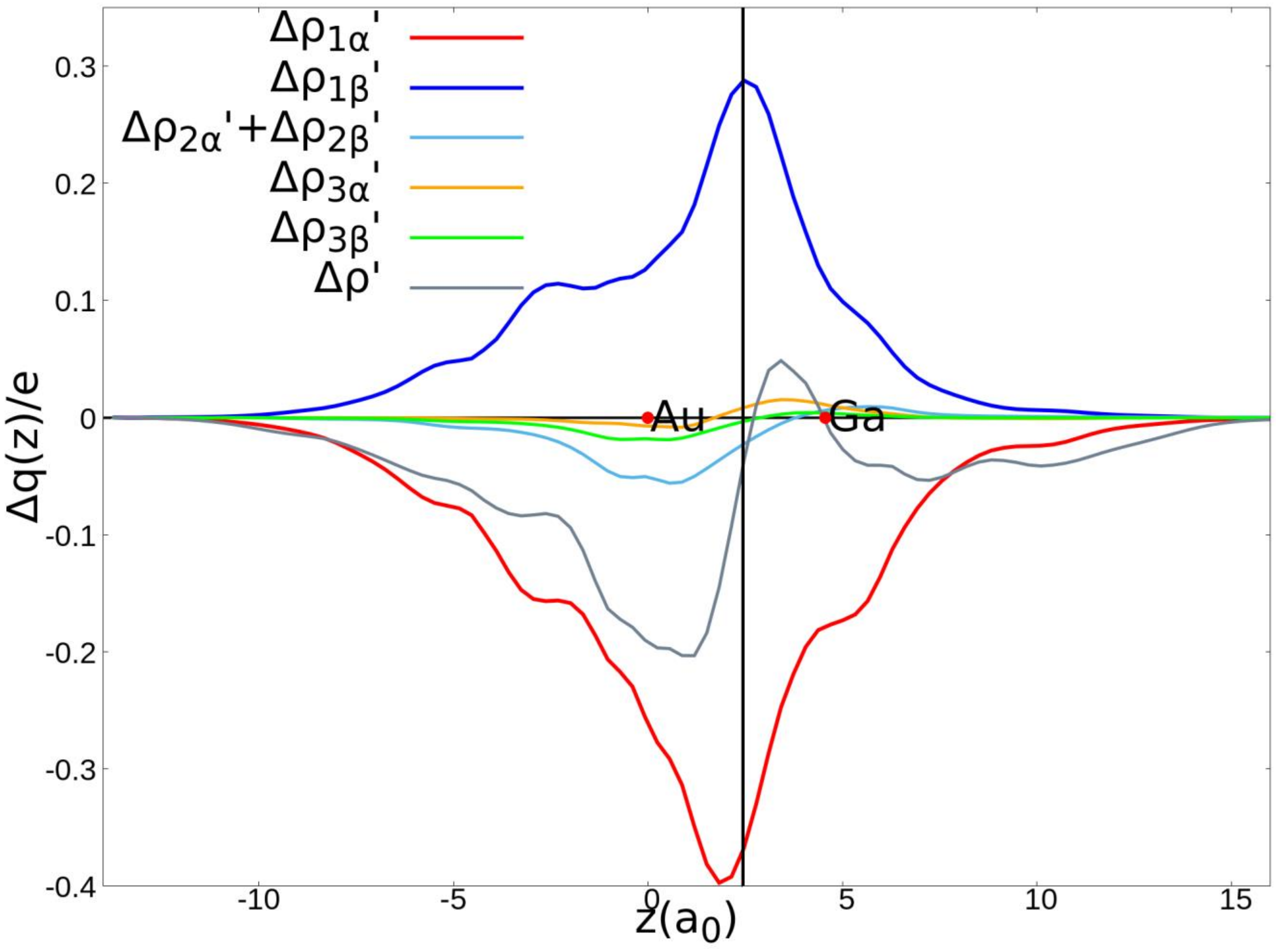

Figure S18. Charge Displacement (CD-NOCV) curves for the interaction between doublet [^{t}Bu$_3$PAu]· and [Ga(^{Si}NON')]· fragments in the [^{t}Bu$_3$PAuGa(^{Si}NON')] complex. Red dots indicate the position of the nuclei along the z axis. The vertical solid line marks the isodensity boundary between the fragments. Positive (negative) values of the curve indicate right-to-left (left-to-right) charge transfer.

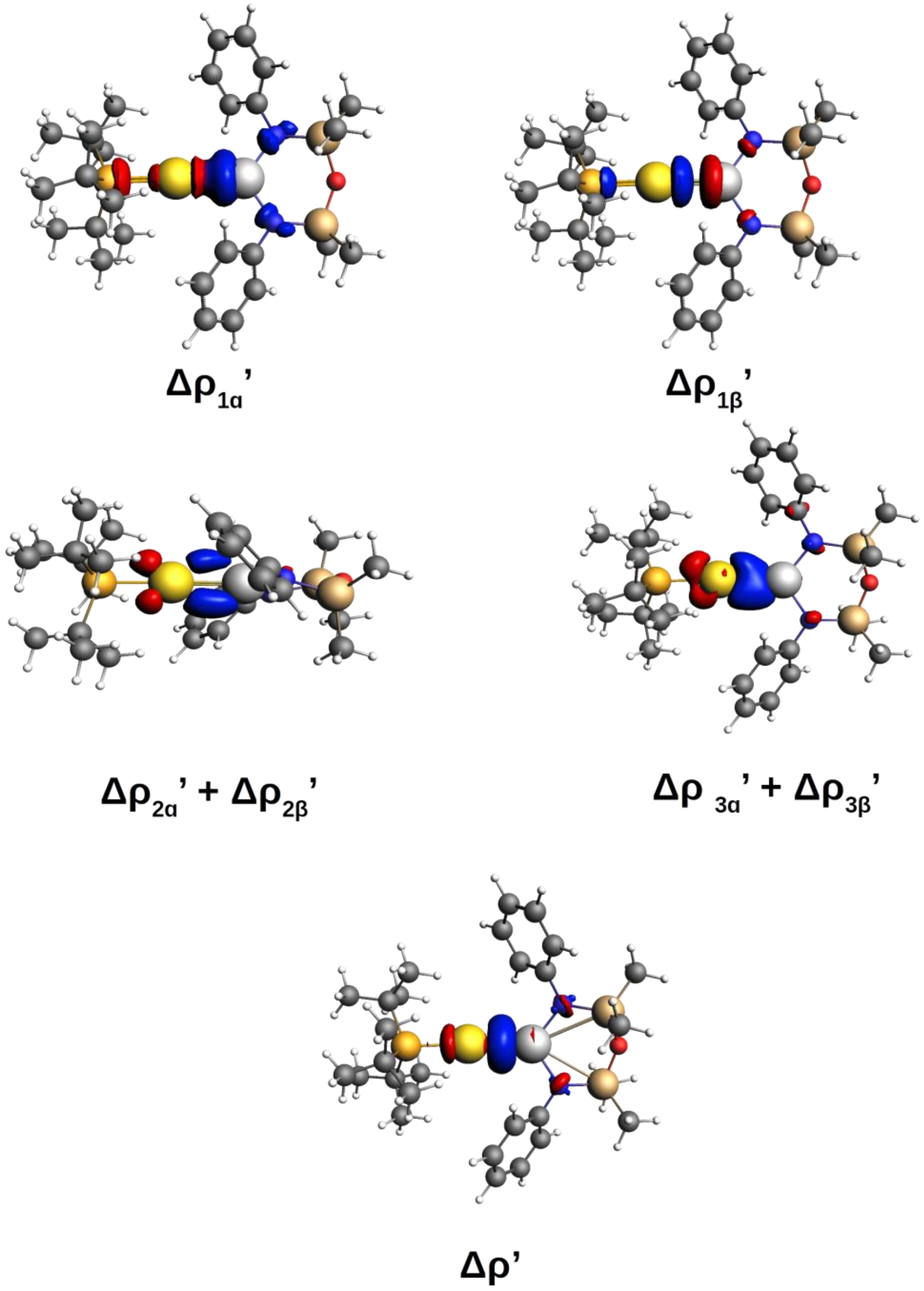

Figure S19. Isodensity surfaces of the $\Delta\rho_{1\alpha}$' and $\Delta\rho_{1\beta}$', $\Delta\rho_{2}$', $\Delta\rho_{3}$' and $\Delta\rho$' NOCV deformation densities for the interaction between doublet [^{t}Bu$_3$PAu]· and [Ga(^{Si}NON')]· fragments in the [^{t}Bu$_3$PAuGa(^{Si}NON')] complex. Blue regions indicate electron density accumulation areas, whereas red regions indicate depletion areas.

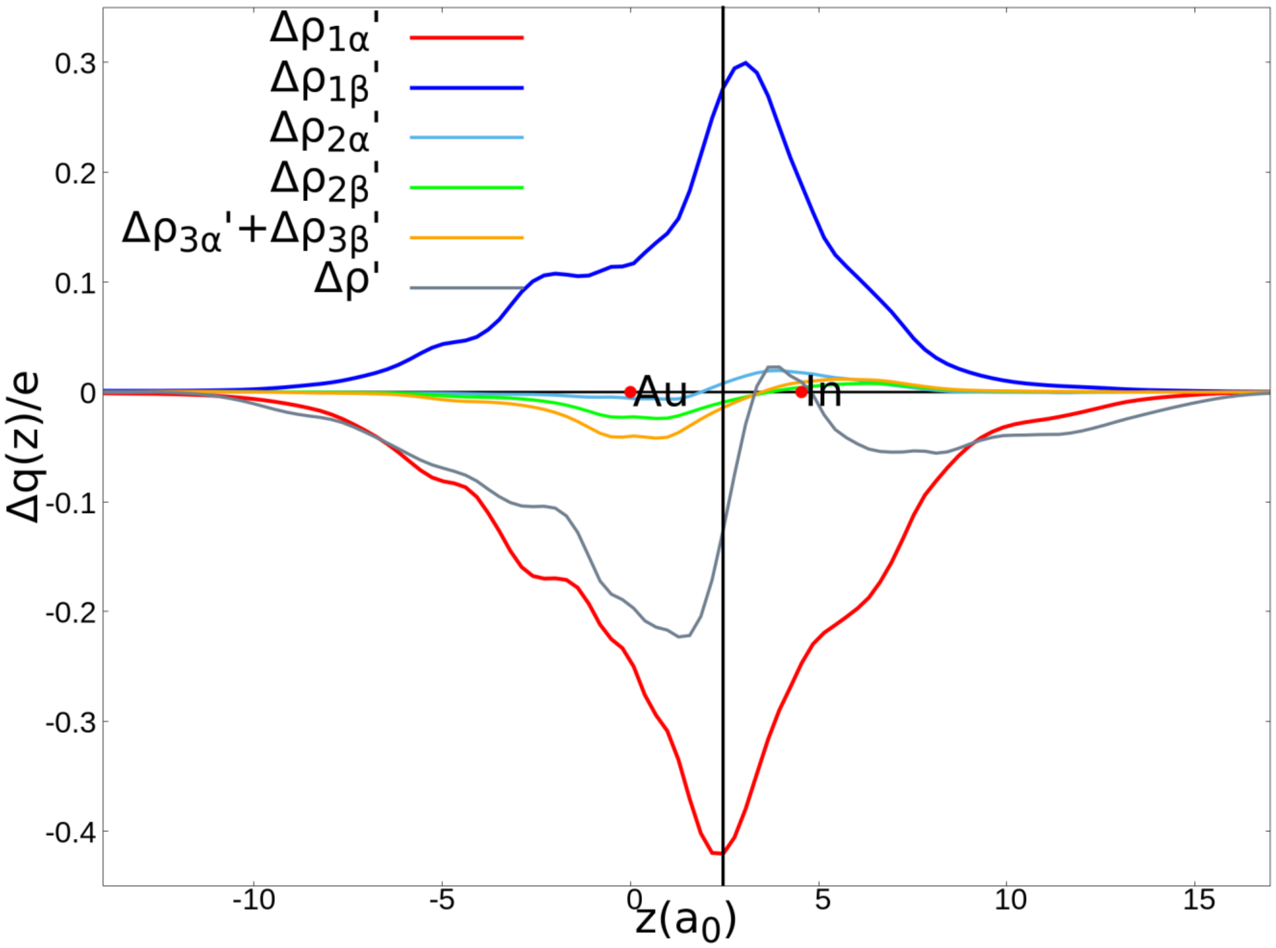

Figure S20. Charge Displacement (CD-NOCV) curves for the interaction between doublet [$^{t}Bu_3PAu$]· and [In(^{Si}NON')]· fragments in the [$^{t}Bu_3PAuIn$(^{Si}NON')] complex. Red dots indicate the position of the nuclei along the z axis. The vertical solid line marks the isodensity boundary between the fragments. Positive (negative) values of the curve indicate right-to-left (left-to-right) charge transfer.

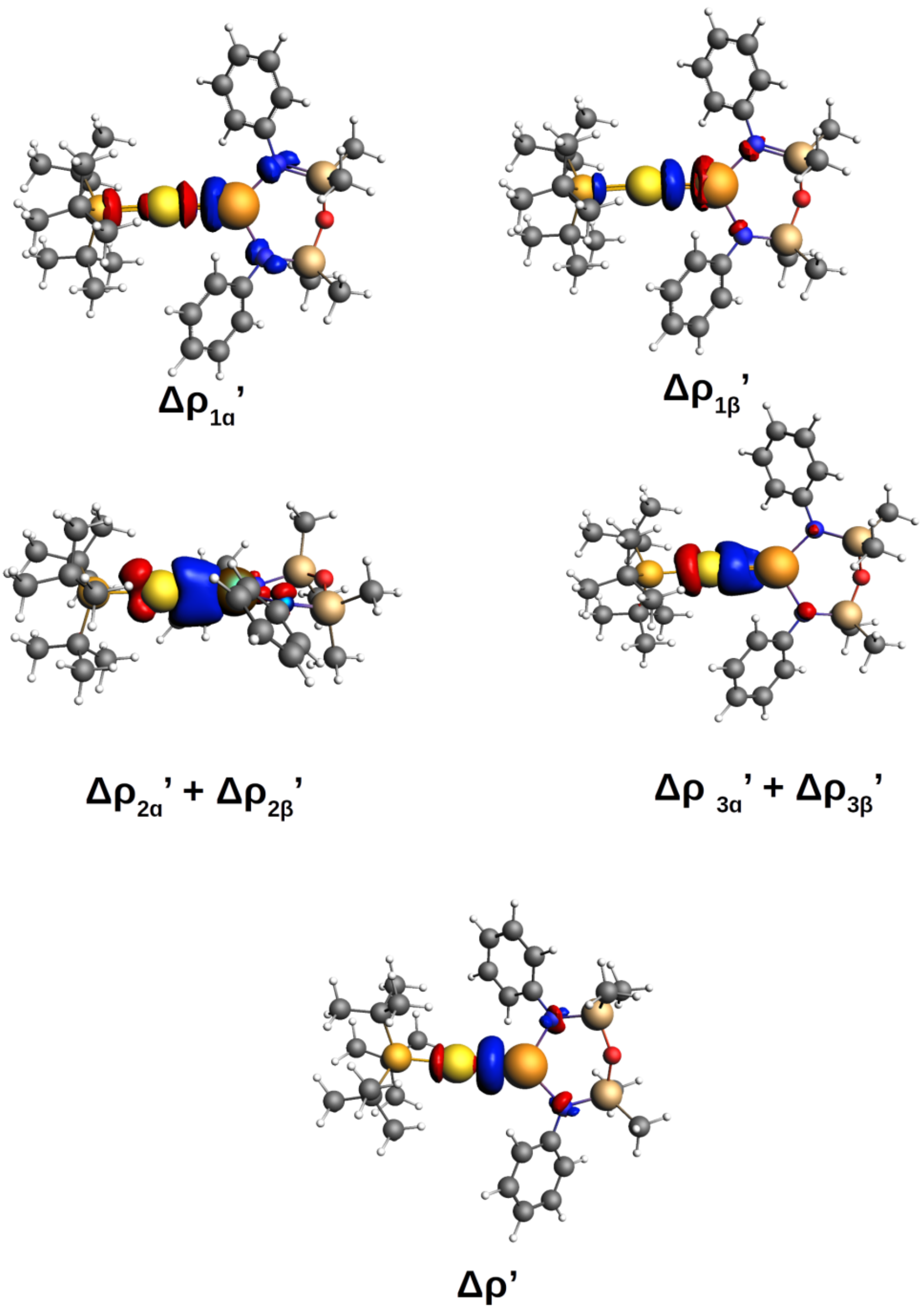

Figure S21. Isodensity surfaces of the $\Delta\rho_{1\alpha}$' and $\Delta\rho_{1\beta}$', $\Delta\rho_{2}$', $\Delta\rho_{3}$' and $\Delta\rho$' NOCV deformation densities for the interaction between doublet [^{t}Bu$_3$PAu]· and [In(^{Si}NON')]· fragments in the [^{t}Bu$_3$PAuIn(^{Si}NON')] complex. Blue regions indicate electron density accumulation areas, whereas red regions indicate depletion areas.

	I^{Al}		I^{Ga}		I^{In}	
	**[^{t}Bu$_3$Au]**	**[Al (^{Si}NON')]**	**[^{t}Bu$_3$Au]**	**[Ga (^{Si}NON')]**	**[^{t}Bu$_3$Au]**	**[In (^{Si}NON')]**
**IE**	5.36	7.01	5.26	6.93	5.26	6.81
**EA**	-0.23	-1.54	-0.17	-2.15	-0.14	-2.56
**M**	-2.80	-4.29	-2.73	-4.54	-2.70	-4.68
**H**	5.13	5.49	5.12	4.78	4.25	6.33
**ω^-**	0.76	1.68	0.73	2.15	0.71	2.58
**N**	3.08	1.71	3.19	1.45	3.23	1.29

Table S15. Electron attachment energy (EA), electronic chemical potential (μ), molecular hardness (η), electrophilicity (ω^-) and nucleophilicity (N) indexes calculated for the neutral [^{t}Bu$_3$Au]· and [X(^{Si}NON')]· (X=B,Al, Ga, In) fragments in complexes **I^{Al}**, **I^{Ga}** and **I^{In}**. The calculations have been carried out in gas phase and fragments have been kept frozen at their in-adduct geometries. Descriptors are expressed in eV.

	**[Al(^{Si}NON)]$^-$**	**[Ga(^{Si}NON)]$^-$**	**[In(^{Si}NON)]$^-$**
**Proton affinity**	-354.5	-334.8	-294.2
**ε_{HOMO} (eV)**	-0.356	-1.095	-1.320
**HOMO composition**	59.0% 3s Al<br>22.4% 3p$_x$ Al<br>4.6% 2p$_x$+2p$_y$ N<br>2.2% 2p$_z$ N<br>1.8% 4s Al	35.8% 4s Ga<br>17.4% 4p$_x$ Ga<br>11.0% 2p$_z$ N<br>9.5% 2p$_x$+2p$_y$ N<br>2.1% 2p$_z$ C<br>3.0% 2p$_x$ C	15.7% 5s In<br>17.1% 2p$_x$ N<br>12.8% 2p$_z$ N<br>10.5% 5p$_z$ In<br>6.1% 2p$_x$ C<br>6.0% 2p$_z$ C

Table S16. Proton affinity (i.e. energy associated to the $X^- + H^+ \rightarrow XH$ reaction), HOMO energy and relative atomic contributions for the three [X(^{Si}NON')]$^-$ (X= Al, Ga, In) anions. The geometry of the anions has been optimized at the PBE/TZ2P level in gas phase.

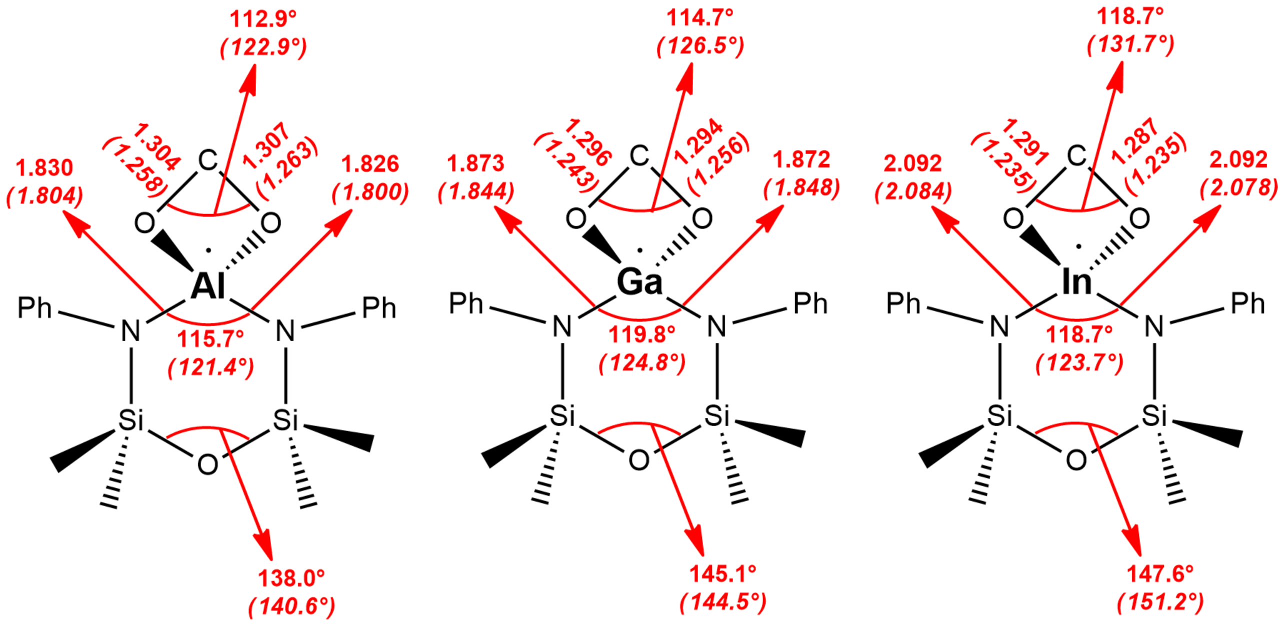

Figure S22. Schematic representation of product-like structures of doublet open shell neutral [CO_2X(^{Si}NON')]· (X=Al, Ga, In) species. Values in parenthesis represent the bond lengths and angles for the relaxed [CO_2X(^{Si}NON')]· geometries.

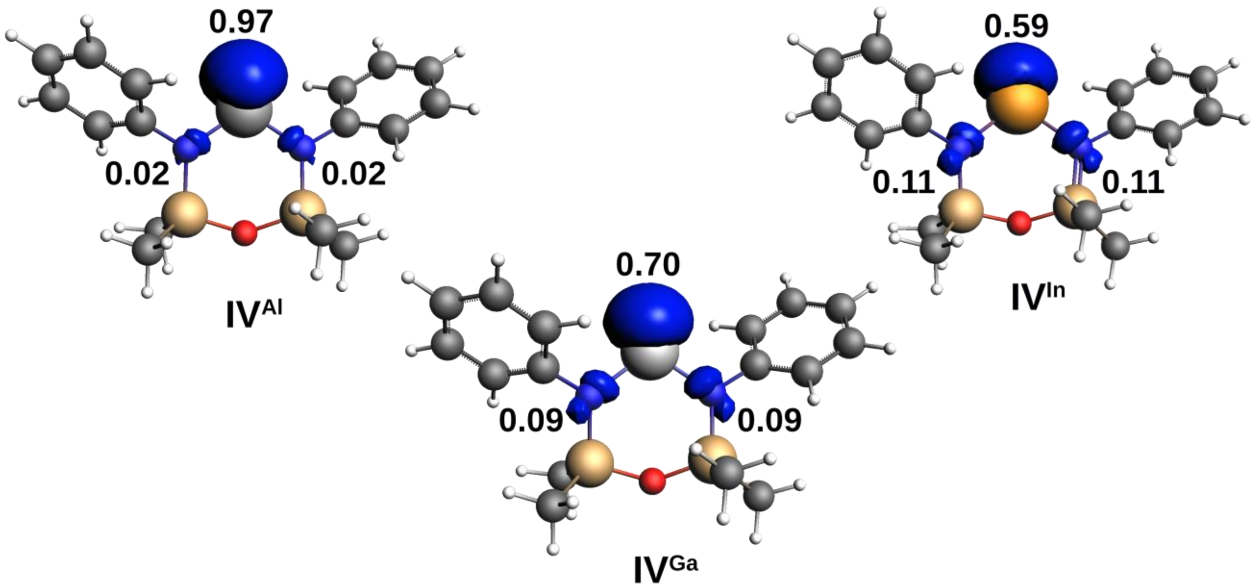

Figure S23. Spin densities (isodensity value: 5 me/a_0) for the doublet open shell neutral [X(^{Si}NON')]· (X=Al, Ga, In) species.

References

(1) Mitoraj, M.; Michalak, A. Natural Orbitals for Chemical Valence as Descriptors of Chemical Bonding in Transition Metal Complexes. *J. Mol. Model.* **2007**, *13* (2), 347–355.

(2) Michalak, A.; Mitoraj, M.; Ziegler, T. Bond Orbitals from Chemical Valence Theory. *J. Phys. Chem. A* **2008**, *112* (9), 1933–1939.

(3) Nalewajski, R. F.; ozek, J. Modified Valence Indices from the Two□particle Density Matrix. *Int. J. Quantum Chem.* **1994**, *51* (4), 187–200.

(4) Nalewajski, R. F.; Mrozek, J.; Michalak, A. Two-Electron Valence Indices from the Kohn-Sham Orbitals. *Int. J. Quantum Chem.* **1997**, *61* (3), 589–601.

(5) Lu, T.; Chen, F. Bond Order Analysis Based on the Laplacian of Electron Density in Fuzzy Overlap Space. *J. Phys. Chem. A* **2013**, *117* (14), 3100–3108.

(6) Bistoni, G.; Rampino, S.; Tarantelli, F.; Belpassi, L. Charge-Displacement Analysis via Natural Orbitals for Chemical Valence: Charge Transfer Effects in Coordination Chemistry. *J. Chem. Phys.* **2015**, *142* (8), 084112.

(7) Belpassi, L.; Infante, I.; Tarantelli, F.; Visscher, L. The Chemical Bond between Au(I) and the Noble Gases. Comparative Study of NgAuF and NgAu+ (Ng = Ar, Kr, Xe) by Density Functional and Coupled Cluster Methods. *J. Am. Chem. Soc.* **2008**, *130* (3), 1048–1060.

(8) Salvi, N.; Belpassi, L.; Tarantelli, F. On the Dewar-Chatt-Duncanson Model for Catalytic Gold(I) Complexes. *Chem. - A Eur. J.* **2010**, *16* (24), 7231–7240.

(9) Bistoni, G.; Belpassi, L.; Tarantelli, F. Advances in Charge Displacement Analysis. *J. Chem. Theory Comput.* **2016**, *12* (3), 1236–1244.

(10) Ziegler, T.; Rauk, A. On the Calculation of Bonding Energies by the Hartree Fock Slater Method. *Theor. Chim. Acta* **1977**, *46* (1), 1–10.

(11) Morokdma, K. Molecular Orbital Studies of Hydrogen Bonds. III. C=O □H-O Hydrogen Bond in H2CO □H2O and H2CO □2H2O. *J. Chem. Phys.* **1971**, *55* (3), 1236–1244.

(12) Mitoraj, M. P.; Michalak, A.; Ziegler, T. A Combined Charge and Energy Decomposition Scheme for Bond Analysis. *J. Chem. Theory Comput.* **2009**, *5* (4), 962–975.

(13) Ferná, I.; Bickelhaupt, F. M. The Activation Strain Model and Molecular Orbital Theory: Understanding and Designing Chemical Reactions. *Chem. Soc. Rev* **2014**, *43*, 4953.

(14) Bickelhaupt, F. M.; Houk, K. N. Analyzing Reaction Rates with the Distortion/Interaction-Activation Strain Model. *Angewandte Chemie - International Edition*. Wiley-VCH Verlag August 14, 2017, pp 10070–10086.

(15) Vermeeren, P.; van der Lubbe, S. C. C.; Fonseca Guerra, C.; Bickelhaupt, F. M.; Hamlin, T. A. Understanding Chemical Reactivity Using the Activation Strain Model. *Nat. Protoc.* **2020**, *15* (2), 649–667.

(16) Geerlings, P.; De Proft, F.; Langenaeker, W. Conceptual Density Functional Theory. *Chem. Rev.* **2003**, *103* (5), 1793–1873.

(17) Chermette, H. Chemical Reactivity Indexes in Density Functional Theory. *J Comput Chem* **1999**, *20*, 129154.

(18) Hoffmann, G.; Tognetti, V.; Joubert, L. Can Molecular and Atomic Descriptors Predict the Electrophilicity of Michael Acceptors? *J. Mol. Model. 2018 2410* **2018**, *24* (10), 1–12.

(19) te Velde, G.; Bickelhaupt, F. M.; Baerends, E. J.; Fonseca Guerra, C.; van Gisbergen, S. J. A.; Snijders, J. G.; Ziegler, T. Chemistry with ADF. *J. Comput. Chem.* **2001**, *22* (9), 931–967.

(20) Gázquez, J. L.; Cedillo, A.; Vela, A. Electrodonating and Electroaccepting Powers. *J. Phys. Chem. A* **2007**, *111* (10), 1966–1970.

(21) Pratihar, S.; Roy, S. Reactivity and Selectivity of Organotin Reagents in Allylation and Arylation: Nucleophilicity Parameter as a Guide. *Organometallics* **2011**, *30* (12), 3257–3269.

(22) Fukui, K.; Yonezawa, T.; Shingu, H. A Molecular Orbital Theory of Reactivity in Aromatic Hydrocarbons. *J. Chem. Phys.* **2004**, *20* (4), 722.

(23) Fukui, K.; Yonezawa, T.; Nagata, C.; Shingu, H. Molecular Orbital Theory of Orientation in Aromatic, Heteroaromatic, and Other Conjugated Molecules. *J. Chem. Phys.* **2004**, *22* (8), 1433.

(24) Morell, C.; Grand, A.; Toro-Labbé, A. New Dual Descriptor for Chemical Reactivity. *J. Phys. Chem. A* **2005**, *109* (1), 205–212.

(25) Sorbelli, D.; Belpassi, L.; Belanzoni, P. Reactivity of a Gold-Aluminyl Complex with Carbon Dioxide: A Nucleophilic Gold? *J. Am. Chem. Soc.* **2021**, *143* (36), 14433–14437.

Geometries-xyz

85
Complex I^{Al}

C	1.527215	2.851744	-1.094443
C	2.519404	2.521385	-0.147280
C	3.017614	3.565798	0.658134
C	2.548799	4.871482	0.519882
C	1.552190	5.176291	-0.410871
C	1.043253	4.153146	-1.213623
N	2.964935	1.189724	-0.016154
Si	4.664186	0.811097	0.191173
C	5.224502	0.859414	1.984325
Al	1.665685	-0.124322	0.077285
Au	-0.722828	0.181532	0.155562
P	-3.127048	0.281057	0.110521
C	-3.745644	-1.165671	-0.987114
C	-3.548697	-2.483813	-0.218824
N	2.387517	-1.829106	0.048384
Si	4.044217	-2.192413	-0.385302
C	4.780965	-3.384048	0.860208
C	1.469207	-2.871727	0.312580
C	1.259830	-3.933006	-0.589722
C	0.319371	-4.927865	-0.326721
C	-0.453426	-4.887847	0.836639
C	-0.261272	-3.841343	1.740485
C	0.690637	-2.854327	1.487990
C	4.278065	-2.848379	-2.130633
C	-3.693325	1.956243	-0.629208
C	-5.169843	2.304068	-0.403795
C	-3.821500	0.084652	1.887017
C	-2.975837	-0.984998	2.607721
C	-2.794430	3.061476	-0.037282
C	-3.401681	1.946341	-2.138877
C	5.728369	1.919194	-0.878366
O	4.851605	-0.747496	-0.337594
C	-3.578699	1.395430	2.653741
C	-5.308411	-0.282472	1.972106
C	-5.205782	-1.057533	-1.443867
C	-2.817073	-1.261814	-2.214914
H	0.270452	4.370806	-1.952883
H	1.182085	6.196783	-0.511621
H	2.958418	5.656535	1.157716
H	5.412839	1.850201	-1.928747
H	6.781493	1.610862	-0.812081
H	5.658206	2.970357	-0.568566
H	5.829210	-3.607904	0.616398
H	4.221865	-4.330619	0.868120
H	3.947159	-3.890898	-2.232870
H	5.344700	-2.807192	-2.396551
H	3.721644	-2.234560	-2.852889
H	-0.851290	-3.794353	2.657265
H	-1.194194	-5.662692	1.035259
H	0.179263	-5.735058	-1.047673
H	1.144899	2.066559	-1.748491
H	3.768514	3.341764	1.416610
H	0.853956	-2.053809	2.211121
H	1.824309	-3.954619	-1.521616
H	-3.557807	2.966621	-2.520492
H	-2.359384	1.668069	-2.344802
H	-4.066539	1.280402	-2.698415
H	-3.016377	4.002051	-0.564683
H	-2.960355	3.232306	1.029024
H	-1.730338	2.829646	-0.186595
H	-5.389207	3.255341	-0.913562
H	-5.845144	1.543966	-0.813074
H	-5.408227	2.441742	0.656845
H	-3.808447	1.215354	3.714629
H	-2.528871	1.711245	2.588426
H	-4.219155	2.215442	2.313375
H	-5.602063	-0.316307	3.033082
H	-5.950273	0.452380	1.473065
H	-5.517638	-1.269760	1.545228
H	-3.270022	-0.997681	3.668446
H	-3.115371	-1.992237	2.209155
H	-1.904966	-0.744670	2.548453
H	-3.079434	-2.175358	-2.770353
H	-2.909267	-0.416179	-2.900510
H	-1.765667	-1.341213	-1.902607
H	-3.734499	-3.312280	-0.919051
H	-2.520381	-2.591016	0.151736
H	-4.246842	-2.599382	0.616656
H	-5.462046	-1.961680	-2.018162
H	-5.904283	-0.990738	-0.602191
H	-5.374300	-0.196900	-2.101069
H	6.258113	0.493919	2.072526
H	5.187190	1.874757	2.402801
H	4.580567	0.215779	2.601317
H	4.742661	-2.956633	1.871895

88
RC^{Al}

C	-0.835816	2.648816	1.638989
C	-1.550066	2.871714	0.442482
C	-1.345269	4.100875	-0.215007
C	-0.468521	5.055301	0.298504
C	0.244196	4.810035	1.475193
C	0.053867	3.597604	2.140296
N	-2.400713	1.868776	-0.069519
Si	-4.014823	2.239638	-0.639929
O	-4.781226	0.773712	-0.765945
Si	-4.601088	-0.742958	-0.113311
C	-5.693919	-1.913736	-1.081087
N	-2.906889	-1.144446	-0.321547
Al	-1.628653	0.186483	-0.185593
Au	0.768791	-0.051991	-0.091404
P	3.178205	-0.043233	-0.027336
C	3.786896	-0.098832	1.790162
C	5.245932	0.324620	2.000390
C	-2.447112	-2.474489	-0.363132
C	-1.413480	-2.845241	-1.249650
C	-0.908053	-4.142996	-1.270059
C	-1.432613	-5.123830	-0.425584
C	-2.467745	-4.780380	0.446640
C	-2.960636	-3.476722	0.486811
C	-5.113545	-0.653508	1.693316
C	3.770572	1.582618	-0.855086
C	2.887332	1.830512	-2.095009
C	3.854544	-1.558207	-0.987930
C	3.614742	-1.327653	-2.489555
C	3.481278	2.755907	0.097151
C	5.250355	1.605558	-1.257425
C	-4.890955	3.334864	0.604650
C	-4.086870	2.998871	-2.355292
C	5.338075	-1.871724	-0.757318
C	2.991753	-2.779345	-0.607828
C	2.852532	0.800250	2.625340
C	3.595554	-1.524241	2.333926
H	-0.102798	-4.392459	-1.963137
H	-1.041986	-6.141338	-0.447354
H	-2.889364	-5.531379	1.116836
H	-5.407734	-1.910047	-2.141921
H	-6.744759	-1.600103	-1.004418
H	-5.615548	-2.943718	-0.708776
H	-5.919014	3.550252	0.280568
H	-4.362351	4.290933	0.725219
H	-3.724651	4.036160	-2.365924
H	-5.125526	3.002339	-2.717541
H	-3.479972	2.413880	-3.060682
H	0.592817	3.389763	3.066063
H	0.934824	5.555692	1.869602
H	-0.331192	5.997004	-0.235637

H	-1.015082	-2.092920	-1.931760
H	-3.742867	-3.219648	1.201899
H	-1.005764	1.718794	2.184570
H	-1.862168	4.292829	-1.155266
H	3.834993	-2.268499	-3.016138
H	2.567436	-1.067610	-2.694117
H	4.263940	-0.554107	-2.912628
H	3.262872	-3.611158	-1.275958
H	3.143984	-3.116441	0.420117
H	1.921321	-2.567438	-0.740448
H	5.620212	-2.726749	-1.391639
H	5.989102	-1.031506	-1.024252
H	5.547936	-2.154228	0.280343
H	3.773898	-1.498285	3.419554
H	2.573208	-1.888344	2.171286
H	4.300151	-2.243151	1.903321
H	5.496702	0.204083	3.066033
H	5.945842	-0.290847	1.423485
H	5.417611	1.375535	1.742823
H	3.106367	0.666450	3.688068
H	2.944573	1.862660	2.388997
H	1.800856	0.514218	2.481501
H	3.136121	2.823825	-2.499143
H	3.038069	1.098943	-2.892335
H	1.822196	1.829488	-1.821945
H	3.657436	3.689794	-0.457800
H	2.434662	2.763483	0.429890
H	4.137870	2.768000	0.973271
H	5.485610	2.597664	-1.673736
H	5.919630	1.435222	-0.406661
H	5.481805	0.865498	-2.032023
H	-6.137533	-0.263773	1.787573
H	-5.077325	-1.637840	2.180644
H	-4.437319	0.017243	2.243054
H	-4.932094	2.842068	1.586029
C	-0.610317	-2.219315	2.466664
O	0.174612	-3.025711	2.148346
O	-1.386422	-1.414292	2.816481

88

TSi^{Al}

C	1.440081	2.542466	-1.325011
C	2.460303	2.332072	-0.373110
C	2.997123	3.469707	0.262020
C	2.536918	4.750684	-0.041184
C	1.514814	4.938509	-0.974906
C	0.968633	3.821870	-1.611840
N	2.898122	1.025937	-0.073084
Si	4.599258	0.636274	0.069119
O	4.661165	-1.021190	-0.038042
Si	3.719893	-2.203375	-0.732247
C	4.407280	-3.866226	-0.219135
N	2.117013	-1.955341	-0.069506
Al	1.601266	-0.227276	0.331857
Au	-0.774282	0.213756	0.305529
P	-3.050891	0.303497	-0.486419
C	-3.220660	-1.029659	-1.855951
C	-4.454652	-0.873506	-2.753676
C	1.155986	-2.981464	-0.016496
C	0.318252	-3.124259	1.110360
C	-0.659766	-4.115788	1.167072
C	-0.824913	-5.012462	0.109402
C	0.002394	-4.893321	-1.009903
C	0.968344	-3.890918	-1.077861
C	3.796187	-1.972207	-2.597113
C	-3.405151	2.040751	-1.213925
C	-2.781297	3.085266	-0.265864
C	-4.266738	-0.073218	0.946075
C	-4.332982	1.154845	1.869109
C	-2.647036	2.181793	-2.544623
C	-4.888307	2.357813	-1.442445
C	5.556610	1.429733	-1.332584
C	5.369413	1.057674	1.728659
C	-5.686747	-0.455886	0.510009
C	-3.654915	-1.204822	1.797077
C	-1.938962	-1.003448	-2.714288
C	-3.245623	-2.418189	-1.196243
H	-1.290443	-4.193682	2.054345
H	-1.584816	-5.792664	0.156422
H	-0.113096	-5.580862	-1.849454
H	4.397228	-3.958471	0.875709
H	5.444568	-3.972453	-0.567244
H	3.818074	-4.692948	-0.637629
H	6.617919	1.147537	-1.284442
H	5.491178	2.525552	-1.282483
H	5.482620	2.141373	1.870499
H	6.367063	0.601525	1.807973
H	4.746897	0.667296	2.546074
H	0.174822	3.947989	-2.349830
H	1.151833	5.940564	-1.204336
H	2.973706	5.609992	0.470385
H	0.464760	-2.459375	1.961151
H	1.578573	-3.792735	-1.976399
H	1.029934	1.677437	-1.849732
H	3.769430	3.339808	1.020555
H	-4.886674	0.866904	2.775354
H	-3.331239	1.479630	2.180925
H	-4.859459	2.002150	1.417896
H	-4.285770	-1.341059	2.688672
H	-3.598895	-2.162657	1.275262
H	-2.641642	-0.941225	2.131169
H	-6.304743	-0.597853	1.410357
H	-6.160956	0.322110	-0.099047
H	-5.710024	-1.396062	-0.052166
H	-3.163370	-3.171172	-1.994345
H	-2.391901	-2.566279	-0.522770
H	-4.175334	-2.617247	-0.653085
H	-4.480286	-1.716889	-3.461144
H	-5.391093	-0.889727	-2.184469
H	-4.428137	0.047350	-3.347007
H	-1.993911	-1.836571	-3.431521
H	-1.811098	-0.079750	-3.283442
H	-1.047466	-1.153611	-2.088796
H	-2.875429	4.075090	-0.738227
H	-3.269599	3.134354	0.710142
H	-1.711754	2.885433	-0.109765
H	-2.713365	3.233682	-2.860868
H	-1.583581	1.935734	-2.427527
H	-3.069966	1.569265	-3.347104
H	-4.967777	3.355379	-1.901835
H	-5.369619	1.641493	-2.118006
H	-5.457585	2.384887	-0.506537
H	4.838718	-1.986845	-2.947065
H	3.246755	-2.757897	-3.134018
H	3.355235	-1.002754	-2.871630
H	5.154702	1.108082	-2.303380
C	0.695453	0.376302	2.799176
O	-0.233191	0.855557	3.337448
O	1.790740	-0.091702	2.639646

88

INT^{Al}

C	-0.696422	-4.088028	0.500317
C	-1.010492	-3.069590	-0.424382
C	-0.430166	-3.168039	-1.707191
C	0.436012	-4.210143	-2.031689
C	0.735764	-5.208512	-1.101910
C	0.149967	-5.141916	0.163223
N	-1.858421	-1.999024	-0.081514
Al	-1.678153	-0.297897	-0.774271

Si	-3.162779	-2.197456	1.079475
C	-2.621735	-2.177196	2.880446
O	-4.101738	-0.848120	0.812955
Si	-3.912961	0.795195	1.003970
C	-5.325483	1.660456	0.138001
N	-2.397986	1.099674	0.180468
C	-1.909136	2.424321	0.054611
C	-1.678490	3.222702	1.191815
C	-1.197638	4.525768	1.073869
C	-0.911351	5.065778	-0.181347
C	-1.127296	4.284685	-1.318244
C	-1.631171	2.989227	-1.205956
C	-3.939612	1.222093	2.832987
C	-4.193240	-3.707218	0.694090
Au	0.839221	0.151872	-1.052188
P	2.555812	0.188764	0.605331
C	4.190810	0.001552	-0.377854
C	3.976223	-1.063941	-1.472824
C	2.354000	-1.257311	1.838420
C	2.657846	-2.575595	1.107196
C	2.510582	1.866983	1.519609
C	3.815856	2.221662	2.244559
C	3.244843	-1.143262	3.082945
C	0.877893	-1.337060	2.268785
C	2.157475	2.964025	0.496175
C	1.358567	1.848962	2.536770
C	4.482614	1.315094	-1.123111
C	5.406509	-0.373182	0.479000
H	0.864549	-4.248738	-3.034293
H	1.405362	-6.028090	-1.362172
H	0.364012	-5.911287	0.907017
H	-4.569532	-3.653804	-0.336703
H	-5.053382	-3.760351	1.376517
H	-3.612874	-4.633121	0.800483
H	-4.840371	0.787022	3.290989
H	-3.980202	2.309236	2.985808
H	-5.166556	2.748600	0.147088
H	-6.284205	1.452402	0.633730
H	-5.394156	1.335275	-0.909102
H	-1.030744	5.118493	1.974823
H	-0.525928	6.081478	-0.272311
H	-0.917584	4.690309	-2.309028
H	-0.692528	-2.437401	-2.469759
H	-1.105975	-4.041725	1.508825
H	-1.862532	2.800402	2.178764
H	-1.836058	2.408301	-2.104194
H	5.329563	1.133977	-1.801292
H	3.630908	1.633258	-1.739348
H	4.764949	2.135366	-0.456014
H	4.892564	-1.114900	-2.079882
H	3.775866	-2.063657	-1.081810
H	3.149060	-0.788730	-2.143724
H	6.295921	-0.390811	-0.169287
H	5.594101	0.350733	1.279954
H	5.309843	-1.368770	0.925755
H	2.375661	-3.401567	1.775642
H	2.060587	-2.685743	0.192539
H	3.719137	-2.694564	0.866435
H	3.098132	-2.047009	3.693916
H	4.310238	-1.077880	2.838170
H	2.978067	-0.282584	3.706344
H	0.751155	-2.253909	2.863064
H	0.559834	-0.494509	2.886240
H	0.206772	-1.409703	1.404872
H	2.043768	3.912590	1.041245
H	2.922144	3.109125	-0.270485
H	1.199566	2.756108	0.000448
H	1.228554	2.874619	2.912184
H	0.410386	1.552172	2.071147
H	1.561599	1.204582	3.397976
H	3.666502	3.172490	2.778553
H	4.102655	1.466814	2.985339
H	4.654130	2.365164	1.554157
H	-3.502494	-2.071752	3.530340
H	-2.098815	-3.097849	3.171999
H	-1.948507	-1.331589	3.074942
H	-3.062219	0.828580	3.362943
O	-1.698167	-0.127792	-2.586261
C	-0.404534	0.159377	-2.741834
O	0.116710	0.409358	-3.816695

88

TSi^{Al}

C	3.114512132	4.463323677	-0.693933567
C	3.056251750	3.240616338	-1.384497154
C	2.578873183	3.244487514	-2.705460287
C	2.177560717	4.431643185	-3.314997178
C	2.255828940	5.644521689	-2.626231338
C	2.726870051	5.652277920	-1.312216500
N	3.484251914	2.030435479	-0.763396235
Al	2.399349108	0.590800435	-0.664357355
Si	5.164756866	1.888405293	-0.272641661
C	5.610275728	2.923370749	1.227927093
O	5.328497993	0.282349834	0.117728410
Si	4.524997775	-0.879863496	0.988862999
C	5.317024908	-2.536673476	0.652016907
N	2.887222205	-0.840844825	0.333524320
C	2.021309850	-1.945300124	0.546388842
C	1.846714466	-2.497195329	1.828773334
C	1.027820751	-3.607939532	2.026105652
C	0.338462406	-4.179773628	0.955601332
C	0.484596969	-3.626362359	-0.318168438
C	1.324251395	-2.533802599	-0.523574233
C	4.626712178	-0.423614924	2.807840466
C	6.296333307	2.287668553	-1.703984840
Au	-1.755565055	-0.083800090	-0.418450873
P	-3.419038334	-0.344640157	1.209762250
C	-4.775696540	0.984333281	0.956379324
C	-4.084386442	2.319318770	0.612036424
C	-2.614333162	-0.139986942	2.936073233
C	-2.295857309	1.347806428	3.159291512
C	-4.182860066	-2.095049625	1.049851557
C	-5.513948139	-2.288552775	1.787750002
C	-3.462040877	-0.654430907	4.106619879
C	-1.253628873	-0.863737484	2.917904728
C	-4.367534851	-2.403091933	-0.449915006
C	-3.163970351	-3.125160614	1.563903672
C	-5.604405115	0.608531493	-0.283671334
C	-5.716268273	1.181124971	2.151923508
H	1.806355068	4.408054775	-4.340515973
H	1.948304895	6.573100063	-3.107769557
H	2.784263558	6.589348983	-0.756331666
H	6.111495635	1.606390035	-2.545642592
H	7.350943827	2.199908448	-1.407144414
H	6.124092121	3.316237294	-2.051660231
H	5.669384775	-0.198782890	3.075417007
H	4.283887107	-1.242019414	3.455447729
H	4.752177843	-3.348422327	1.130310642
H	6.345021891	-2.558569936	1.040274104
H	5.347969377	-2.729736697	-0.428991243
H	0.918131530	-4.019463665	3.030662002
H	-0.307880087	-5.043535187	1.111855469
H	-0.049726379	-4.053874773	-1.167564032
H	2.522592460	2.301625902	-3.250676464
H	3.452973211	4.471586297	0.342000855
H	2.347710796	-2.036369653	2.679883614
H	1.448533581	-2.131702456	-1.529757793
H	-6.257699350	1.460606239	-0.523682016
H	-4.963791534	0.423123710	-1.156004424
H	-6.247570381	-0.262030318	-0.119960394

H	-4.864095739	3.044459621	0.333289002
H	-3.517471039	2.743156166	1.444061762
H	-3.403620418	2.201346221	-0.242424694
H	-6.482107483	1.922067630	1.874807898
H	-6.234501356	0.258266059	2.435355392
H	-5.193215997	1.568466735	3.033435000
H	-1.676380290	1.426974627	4.065275638
H	-1.719697593	1.765331637	2.322809844
H	-3.190219488	1.958792440	3.318522859
H	-2.929843615	-0.435387164	5.045264693
H	-4.443113480	-0.169478790	4.160855876
H	-3.614611671	-1.738568782	4.065335027
H	-0.738267132	-0.647038816	3.866226565
H	-1.331161435	-1.948423902	2.821794278
H	-0.621892924	-0.503206107	2.094610405
H	-4.680141399	-3.454144517	-0.544857433
H	-5.127765591	-1.784700062	-0.932512744
H	-3.422946769	-2.274619648	-0.996484129
H	-3.536329633	-4.126625580	1.299950645
H	-2.182464770	-3.000292142	1.089218078
H	-3.039339374	-3.099119497	2.651319991
H	-5.825272814	-3.338407250	1.673204086
H	-5.433080672	-2.082098509	2.860837472
H	-6.315527954	-1.665765971	1.375066819
H	6.593182909	2.611978619	1.611104250
H	5.670685958	3.990843235	0.976060144
H	4.872626238	2.799561538	2.032700910
H	4.017246524	0.464311477	3.026766539
O	0.858870262	0.736226609	-1.380766948
C	-0.257039729	0.070359546	-1.818617978
O	-0.263581815	-0.383411611	-2.953229428

88

PC^{Al}

C	-4.157781	-3.998070	0.461432
C	-3.751234	-2.931677	-0.364208
C	-3.143321	-3.252561	-1.593308
C	-2.949115	-4.578334	-1.976497
C	-3.372846	-5.625493	-1.154563
C	-3.980272	-5.323426	0.065973
N	-3.953075	-1.579492	0.010280
Si	-5.529998	-1.073096	0.599141
C	-6.866976	-1.743746	-0.529470
O	-5.504264	0.584794	0.553317
Si	-4.706365	1.784713	-0.272962
C	-5.047601	3.418253	0.571730
N	-3.011897	1.365739	-0.144544
Al	-2.557219	-0.405366	-0.071008
O	-1.025810	-0.780977	0.969876
C	-0.331695	-0.781339	-0.138272
Au	1.692342	-0.544496	-0.202414
P	3.954068	0.014211	-0.315150
C	4.989309	-1.544162	-0.715120
C	4.760102	-1.925001	-2.187316
C	-2.005264	2.352493	-0.259589
C	-1.991232	3.275380	-1.324348
C	-0.996193	4.246621	-1.424725
C	0.026044	4.317348	-0.475578
C	0.028573	3.406465	0.582797
C	-0.973349	2.443881	0.695225
C	-5.356036	1.788050	-2.037137
C	-5.884802	-1.513562	2.388955
C	4.489221	0.751621	1.369251
C	5.808998	1.533081	1.330653
C	4.151992	1.323247	-1.701213
C	3.275881	0.902879	-2.898589
C	3.358087	1.666410	1.880141
C	4.603309	-0.389599	2.393795
C	3.571780	2.660205	-1.209215
C	5.598462	1.538949	-2.165399
C	6.494051	-1.390778	-0.459306
C	4.433723	-2.718397	0.115797
H	0.812091	3.450650	1.340947
H	0.807865	5.072763	-0.559274
H	-1.015227	4.947661	-2.260781
H	-4.726839	3.377963	1.621952
H	-6.122314	3.646753	0.544325
H	-4.509450	4.240286	0.080576
H	-7.859360	-1.415216	-0.189488
H	-6.856120	-2.842439	-0.543427
H	-6.106893	-2.580790	2.522978
H	-6.757341	-0.942729	2.739633
H	-5.028297	-1.255411	3.027578
H	-2.474546	-4.793463	-2.935167
H	-3.229990	-6.662067	-1.460450
H	-4.310821	-6.127141	0.725853
H	-0.971654	1.748681	1.534442
H	-2.764513	3.212243	-2.090886
H	-2.830034	-2.443839	-2.254577
H	-4.601291	-3.779015	1.432430
H	4.740869	0.062434	3.387135
H	3.687569	-0.994688	2.428060
H	5.459991	-1.045887	2.210471
H	3.611094	1.980790	2.903866
H	3.223079	2.567106	1.277815
H	2.399572	1.130145	1.913850
H	6.049120	1.858334	2.354425
H	6.647394	0.926810	0.970324
H	5.741628	2.433961	0.710724
H	3.540390	3.347031	-2.068069
H	2.543454	2.549720	-0.840324
H	4.184694	3.133387	-0.435361
H	5.605247	2.347428	-2.912230
H	6.262640	1.838724	-1.347154
H	6.020936	0.649619	-2.646215
H	3.313623	1.711277	-3.644282
H	3.609628	-0.016156	-3.385737
H	2.228527	0.770906	-2.593656
H	4.950901	-3.635214	-0.205103
H	4.590397	-2.605997	1.190898
H	3.357920	-2.853896	-0.062739
H	5.224088	-2.908097	-2.355871
H	3.690906	-2.016904	-2.419693
H	5.218563	-1.220277	-2.888194
H	6.996292	-2.318619	-0.773170
H	6.935036	-0.565681	-1.029725
H	6.723669	-1.240189	0.601249
H	-6.450062	1.900833	-2.043476
H	-4.927704	2.609345	-2.628600
H	-5.105811	0.842680	-2.539977
H	-6.715910	-1.387611	-1.557993
O	-1.079546	-0.723304	-1.205294

85

Complex I^{Ga}

C	2.514532	-3.663916	-0.786603
C	2.193352	-2.639511	0.129696
C	1.255962	-2.948570	1.140019
C	0.661018	-4.205609	1.218051
C	0.995712	-5.209043	0.305867
C	1.932492	-4.926740	-0.692154
N	2.752634	-1.356623	0.048117
GA	1.511045	0.093669	0.144729
Au	-0.904695	-0.073254	0.112888
P	-3.264641	-0.153037	0.048321
C	-3.864507	-1.826826	0.762829
C	-2.947786	-2.939550	0.214576
Si	4.411174	-1.033573	-0.397468
C	4.633743	-0.830398	-2.253422
O	4.796036	0.412331	0.317684

Si	4.138173	1.918174	0.532111
C	4.479542	2.368727	2.323699
N	2.443521	1.767082	0.127235
C	1.603899	2.874937	-0.081744
C	0.680743	2.877992	-1.151029
C	-0.207589	3.934848	-1.340650
C	-0.189641	5.038816	-0.485907
C	0.734222	5.065588	0.562434
C	1.611549	4.001753	0.766559
C	4.956424	3.147682	-0.624127
C	5.567583	-2.336809	0.288712
C	-3.859067	0.035030	-1.765028
C	-3.580425	-1.279933	-2.512546
C	-3.935430	1.298128	1.106600
C	-5.415694	1.184859	1.491686
C	-5.340564	0.401002	-1.923194
C	-2.981396	1.102880	-2.448938
C	-3.069391	1.401830	2.378746
C	-3.707325	2.613896	0.342359
C	-3.642552	-1.813266	2.284643
C	-5.331000	-2.167538	0.469093
H	-0.062008	-4.406622	2.010580
H	0.537742	-6.196045	0.372966
H	2.205345	-5.695600	-1.417228
H	5.432552	-2.431244	1.375173
H	6.611181	-2.051665	0.093327
H	5.390573	-3.320788	-0.164722
H	6.028794	3.240155	-0.401016
H	4.498981	4.142409	-0.526855
H	4.214352	3.410335	2.550866
H	5.549937	2.238966	2.541810
H	3.910873	1.712901	2.998189
H	-0.910885	3.899552	-2.174503
H	-0.880442	5.868594	-0.636468
H	0.763884	5.919372	1.241541
H	1.011937	-2.182548	1.877620
H	3.214685	-3.454968	-1.596610
H	0.683616	2.036716	-1.845466
H	2.295593	4.023294	1.614985
H	-3.822238	-2.831429	2.661056
H	-2.608982	-1.541292	2.537466
H	-4.328459	-1.142011	2.811504
H	-3.194589	-3.873765	0.741894
H	-3.074657	-3.120298	-0.855335
H	-1.888694	-2.714099	0.401361
H	-5.576993	-3.116207	0.971013
H	-6.022908	-1.404844	0.843754
H	-5.519250	-2.308871	-0.601078
H	-3.755318	-1.103168	-3.584312
H	-2.536697	-1.599435	-2.392399
H	-4.241097	-2.095698	-2.202267
H	-5.580734	0.429443	-2.997386
H	-6.005967	-0.332311	-1.453764
H	-5.571777	1.389948	-1.512209
H	-3.228401	1.112422	-3.521404
H	-3.139665	2.111035	-2.059688
H	-1.914607	0.861253	-2.342607
H	-3.373623	2.307733	2.924933
H	-3.180930	0.550927	3.054699
H	-2.005479	1.500027	2.119729
H	-3.930932	3.443835	1.029357
H	-2.662304	2.723128	0.021973
H	-4.364523	2.722929	-0.526432
H	-5.703078	2.091609	2.046226
H	-6.072437	1.108831	0.617793
H	-5.611911	0.328071	2.146211
H	5.665809	-0.538327	-2.495954
H	4.405562	-1.759896	-2.794204
H	3.958645	-0.047174	-2.628494
H	4.845045	2.821399	-1.667670

88

RC^{Ga}

C	-0.708403	2.679151	1.706813
C	-1.487188	2.907111	0.550110
C	-1.333242	4.152825	-0.094364
C	-0.446362	5.112332	0.390110
C	0.330773	4.860007	1.523898
C	0.191044	3.633717	2.177018
N	-2.346047	1.907344	0.068180
SI	-3.940129	2.239452	-0.568279
O	-4.707921	0.773544	-0.652017
SI	-4.528452	-0.756741	-0.038301
C	-5.641860	-1.895069	-1.023159
N	-2.846752	-1.171373	-0.276097
GA	-1.518111	0.181006	-0.061247
AU	0.892665	-0.027326	0.016375
P	3.254700	-0.034730	0.012288
C	3.900776	-0.041648	1.816357
C	5.370274	0.368827	1.975040
C	-2.367559	-2.485077	-0.375216
C	-1.314254	-2.799633	-1.262176
C	-0.797715	-4.090159	-1.344979
C	-1.326606	-5.120141	-0.563640
C	-2.378501	-4.832381	0.309087
C	-2.884308	-3.537324	0.411835
C	-5.018599	-0.707505	1.775234
C	3.833273	1.561343	-0.879949
C	2.924727	1.779233	-2.106707
C	3.888639	-1.584833	-0.919426
C	3.619441	-1.396391	-2.421986
C	3.578734	2.766086	0.042430
C	5.303805	1.555379	-1.317105
C	-4.863287	3.384110	0.595769
C	-3.961013	2.918932	-2.319605
C	5.374704	-1.901257	-0.708689
C	3.027835	-2.788675	-0.485175
C	3.001378	0.895983	2.646646
C	3.703987	-1.446935	2.407932
H	0.019837	-4.295187	-2.038332
H	-0.926788	-6.131800	-0.634273
H	-2.803319	-5.621974	0.931212
H	-5.366525	-1.864732	-2.086497
H	-6.689207	-1.574792	-0.927543
H	-5.568862	-2.935622	-0.680289
H	-5.876906	3.586230	0.221273
H	-4.339913	4.344479	0.703281
H	-3.601258	3.955845	-2.371455
H	-4.987847	2.899461	-2.713737
H	-3.330910	2.302851	-2.976816
H	0.777664	3.419821	3.072082
H	1.029077	5.610372	1.895194
H	-0.351482	6.064441	-0.134920
H	-0.913391	-2.008194	-1.896877
H	-3.679698	-3.325550	1.127453
H	-0.839394	1.740360	2.247584
H	-1.899165	4.354427	-1.003993
H	3.823334	-2.354201	-2.923652
H	2.569607	-1.136313	-2.612815
H	4.264211	-0.640448	-2.881848
H	3.289072	-3.642489	-1.128864
H	3.192773	-3.091549	0.551322
H	1.956283	-2.580291	-0.611108
H	5.637893	-2.773773	-1.326671
H	6.026294	-1.073102	-1.009468
H	5.601911	-2.160091	0.331393
H	3.903449	-1.388743	3.488440
H	2.674329	-1.803261	2.275697
H	4.391558	-2.187489	1.986642
H	5.646067	0.276837	3.037104

H	6.047749	-0.272523	1.399885
H	5.547511	1.409491	1.681911
H	3.290874	0.799218	3.704091
H	3.092208	1.948000	2.368035
H	1.943822	0.612640	2.550461
H	3.180270	2.753362	-2.551013
H	3.044544	1.017713	-2.880796
H	1.866659	1.806568	-1.809334
H	3.748863	3.679872	-0.546472
H	2.541008	2.794323	0.401409
H	4.258053	2.799163	0.900367
H	5.537840	2.531784	-1.768972
H	5.991481	1.405256	-0.477353
H	5.509694	0.789709	-2.073580
H	-6.046893	-0.335611	1.892043
H	-4.958703	-1.700583	2.242397
H	-4.344038	-0.036340	2.326611
H	-4.945837	2.926912	1.591709
C	-0.496844	-2.387590	2.438692
O	0.320530	-3.127933	2.048536
O	-1.302312	-1.647015	2.856309

88

TSI^{Ga}

C	1.481631	2.621597	-1.157124
C	2.614763	2.228055	-0.413811
C	3.424443	3.250688	0.121046
C	3.117003	4.594723	-0.084949
C	1.982439	4.965393	-0.810681
C	1.164426	3.964867	-1.341250
N	2.913388	0.867831	-0.230314
SI	4.545314	0.226382	-0.254203
O	4.364024	-1.401447	-0.521803
SI	3.210146	-2.445755	-1.094657
C	3.807629	-4.188676	-0.772975
N	1.768898	-2.087009	-0.159114
GA	1.485853	-0.264492	0.276204
AU	-0.835678	0.399401	0.521204
P	-2.998204	0.528999	-0.464655
C	-3.085302	-0.798078	-1.844547
C	-4.255847	-0.610836	-2.818864
C	0.730172	-3.000258	0.068461
C	0.020603	-2.996972	1.288843
C	-1.010731	-3.903460	1.526092
C	-1.358144	-4.855899	0.565143
C	-0.664355	-4.874590	-0.647167
C	0.351485	-3.955041	-0.899101
C	2.977148	-2.109908	-2.928788
C	-3.255351	2.276858	-1.203856
C	-2.673827	3.303793	-0.211467
C	-4.309652	0.171730	0.883504
C	-4.417345	1.398059	1.804819
C	-2.417557	2.408357	-2.486722
C	-4.715967	2.622432	-1.521987
C	5.514182	1.001011	-1.658132
C	5.450259	0.382266	1.379738
C	-5.701071	-0.181797	0.342504
C	-3.782416	-0.974666	1.770070
C	-1.751535	-0.794415	-2.619119
C	-3.183289	-2.185971	-1.192300
H	-1.535656	-3.874738	2.482299
H	-2.157160	-5.572096	0.756822
H	-0.926489	-5.604147	-1.415220
H	3.945864	-4.347789	0.305348
H	4.774534	-4.345050	-1.272409
H	3.103718	-4.944119	-1.144596
H	6.522861	0.566767	-1.707804
H	5.615037	2.087070	-1.528797
H	5.703081	1.425638	1.613613
H	6.385641	-0.195530	1.354593
H	4.824756	-0.007594	2.195050
H	0.278133	4.232956	-1.918526
H	1.740631	6.017465	-0.961507
H	3.765302	5.361268	0.342424
H	0.318452	-2.294140	2.066619
H	0.854696	-3.963422	-1.866635
H	0.859433	1.845510	-1.607108
H	4.293026	2.983699	0.723483
H	-5.042512	1.116844	2.665184
H	-3.435723	1.699932	2.194125
H	-4.891384	2.258179	1.321385
H	-4.493643	-1.116950	2.597724
H	-3.680206	-1.926822	1.245480
H	-2.805156	-0.720631	2.204056
H	-6.384013	-0.310476	1.196167
H	-6.114721	0.605735	-0.297218
H	-5.702613	-1.121721	-0.220285
H	-3.060202	-2.938363	-1.985103
H	-2.381635	-2.353638	-0.462394
H	-4.151702	-2.367972	-0.715251
H	-4.253796	-1.453268	-3.527296
H	-5.227096	-0.607324	-2.311723
H	-4.169870	0.309184	-3.407531
H	-1.789411	-1.610277	-3.356662
H	-1.558214	0.135366	-3.159101
H	-0.906826	-0.990490	-1.945108
H	-2.726974	4.298259	-0.679917
H	-3.217124	3.352109	0.734887
H	-1.618048	3.089855	0.006084
H	-2.448302	3.462241	-2.800807
H	-1.366848	2.144770	-2.311788
H	-2.802641	1.807190	-3.316336
H	-4.744550	3.621975	-1.982186
H	-5.167954	1.918148	-2.229637
H	-5.342461	2.659942	-0.624121
H	3.922937	-2.241188	-3.474189
H	2.229582	-2.779999	-3.376258
H	2.635787	-1.074957	-3.078328
H	5.009347	0.815734	-2.616557
C	0.544556	0.582347	2.553926
O	-0.254861	1.294448	3.103133
O	1.590878	-0.037349	2.685241

88

INT^{Ga}

C	0.018173	-4.041150	-0.100253
C	-0.555011	-3.012702	-0.877452
C	-0.101510	-2.875021	-2.206241
C	0.894925	-3.704320	-2.716462
C	1.456919	-4.713875	-1.931917
C	1.000083	-4.881371	-0.623326
N	-1.536706	-2.158405	-0.344359
GA	-1.563001	-0.322517	-0.776610
SI	-2.766031	-2.663347	0.804028
C	-2.137666	-2.909851	2.559907
O	-3.823990	-1.380979	0.801735
SI	-3.828398	0.222859	1.243883
C	-5.414068	1.004109	0.641795
N	-2.456902	0.876560	0.372066
C	-2.077209	2.233521	0.418321
C	-1.974037	2.914716	1.648278
C	-1.591106	4.254048	1.700418
C	-1.271690	4.948306	0.532138
C	-1.362863	4.285036	-0.693280
C	-1.771508	2.953821	-0.753918
C	-3.718502	0.331297	3.116625
C	-3.644586	-4.189807	0.184724
AU	0.878222	0.483342	-1.071348
P	2.530012	0.524419	0.658714
C	3.995317	-0.537718	0.028907

C	3.429040	-1.833168	-0.586264
C	1.878281	-0.171001	2.317292
C	1.737056	-1.696548	2.191386
C	3.060267	2.352712	0.865512
C	4.415674	2.546352	1.557145
C	2.769938	0.161619	3.521344
C	0.458642	0.369906	2.564083
C	3.085837	3.008112	-0.530381
C	1.971494	3.097318	1.654923
C	4.684129	0.211079	-1.124769
C	5.039756	-0.882945	1.098846
H	1.224899	-3.565072	-3.747029
H	2.233696	-5.363018	-2.335923
H	1.421396	-5.665810	0.007472
H	-4.107976	-3.993654	-0.791588
H	-4.428420	-4.501809	0.889171
H	-2.938116	-5.023323	0.067683
H	-4.465878	-0.344805	3.557794
H	-3.934761	1.346298	3.476352
H	-5.388036	2.091568	0.801849
H	-6.284476	0.600353	1.178208
H	-5.547008	0.819810	-0.432981
H	-1.522629	4.751746	2.669086
H	-0.959006	5.991652	0.575280
H	-1.129352	4.811475	-1.619787
H	-0.558860	-2.124122	-2.848049
H	-0.295802	-4.168290	0.935371
H	-2.174381	2.376153	2.572830
H	-1.884428	2.466071	-1.720572
H	5.412732	-0.475062	-1.581333
H	3.970440	0.499998	-1.908418
H	5.230889	1.100396	-0.795421
H	4.266331	-2.388105	-1.035621
H	2.942871	-2.493444	0.134441
H	2.702642	-1.620361	-1.382391
H	5.863472	-1.428981	0.614015
H	5.466456	0.007275	1.574504
H	4.634919	-1.534563	1.881019
H	1.208820	-2.058373	3.086118
H	1.140174	-1.983030	1.316132
H	2.701124	-2.213132	2.150295
H	2.350815	-0.338202	4.408033
H	3.798747	-0.192870	3.396402
H	2.796306	1.235755	3.734850
H	0.067562	-0.118997	3.469491
H	0.419925	1.448366	2.726532
H	-0.217367	0.130149	1.734191
H	3.307128	4.077933	-0.398065
H	3.841955	2.591666	-1.199604
H	2.104837	2.932093	-1.022129
H	2.197639	4.172717	1.604590
H	0.974410	2.954413	1.219266
H	1.944911	2.817866	2.713121
H	4.593777	3.626187	1.675212
H	4.445712	2.094749	2.554850
H	5.247182	2.142002	0.969299
H	-2.986039	-2.960146	3.257539
H	-1.565270	-3.841794	2.663849
H	-1.489077	-2.078205	2.865657
H	-2.728413	0.034074	3.486879
O	-1.594050	0.129853	-2.659932
C	-0.336606	0.546664	-2.772154
O	0.146577	0.960549	-3.815388

88

TSII^{Ga}

C	-4.515696645	-3.161283726	1.137319597
C	-4.018312480	-2.371539495	0.085640815
C	-3.714079755	-3.002288998	-1.132927062
C	-3.905093536	-4.372712224	-1.292375214
C	-4.418989208	-5.145835196	-0.248014186
C	-4.724149230	-4.530238450	0.966714290
N	-3.863582873	-0.970087532	0.251032927
GA	-2.336212132	-0.111242774	-0.346523355
SI	-5.215900137	0.018849504	0.789780766
C	-5.574221715	-0.111787964	2.627005365
O	-4.732630453	1.572713671	0.470060489
SI	-3.436848353	2.592948211	0.623282398
C	-3.791738597	4.173394385	-0.303173791
N	-2.110688602	1.718748318	-0.144474959
C	-0.989157374	2.411157951	-0.658498620
C	-0.347433432	3.402955991	0.106352224
C	0.706943037	4.144786545	-0.423329789
C	1.170850884	3.893102574	-1.715846217
C	0.560999164	2.891068585	-2.473270586
C	-0.512520640	2.168598470	-1.958056470
C	-3.145295553	2.904547804	2.450960397
C	-6.735504134	-0.371625343	-0.222263251
AU	1.756580099	-0.602357603	-0.572157314
P	3.668130866	-0.173493772	0.715669098
C	4.613029372	-1.822806797	0.958501476
C	3.583530007	-2.938158066	1.231568707
C	3.125352003	0.525264360	2.414488419
C	2.497223992	-0.612547565	3.236497163
C	4.793273693	1.093814504	-0.180489800
C	6.236785237	1.155926251	0.336277901
C	4.249251644	1.175605330	3.231245833
C	1.993593420	1.544217509	2.174498959
C	4.789506255	0.757633094	-1.685241901
C	4.155540132	2.486870572	-0.063932552
C	5.290065277	-2.195002463	-0.371887728
C	5.663393706	-1.806928137	2.076400017
H	-3.658203181	-4.838429782	-2.247359466
H	-4.575464866	-6.216681662	-0.379426827
H	-5.115735533	-5.119788014	1.797019337
H	-6.547398046	-0.179242861	-1.287330365
H	-7.587458102	0.243424299	0.099953760
H	-7.013906127	-1.428637927	-0.108294549
H	-4.075319501	3.241677512	2.931102837
H	-2.382464462	3.678098037	2.615579344
H	-2.927067219	4.849884757	-0.282880282
H	-4.646610684	4.695213007	0.149592299
H	-4.032462860	3.954428727	-1.352344760
H	1.179677693	4.914886895	0.188238737
H	2.002836123	4.466091030	-2.125228545
H	0.913464141	2.673487254	-3.482057674
H	-3.318645420	-2.407031076	-1.956239476
H	-4.723450840	-2.695666827	2.100286454
H	-0.675103143	3.582901117	1.130299412
H	-0.994972620	1.408267176	-2.572695508
H	5.678704098	-3.220044115	-0.277788337
H	4.574383662	-2.184629798	-1.204662391
H	6.135219652	-1.544824949	-0.618958512
H	4.117316151	-3.900719415	1.229013561
H	3.079730072	-2.839344400	2.195689054
H	2.816910366	-2.968128531	0.444789397
H	6.181144087	-2.778599859	2.082176506
H	6.421049173	-1.028738597	1.932467634
H	5.213719311	-1.672888627	3.066687700
H	2.024760473	-0.166343231	4.124400038
H	1.716154365	-1.135506637	2.668642736
H	3.233463188	-1.342355927	3.588592058
H	3.839016680	1.485913964	4.204697239
H	5.079361726	0.487445445	3.425611198
H	4.650967464	2.072472310	2.746975986
H	1.593561923	1.846913628	3.154492884
H	2.317574361	2.446426741	1.651835928
H	1.175763326	1.097952954	1.592118094
H	5.333380072	1.555879265	-2.212841164
H	5.276766190	-0.190988967	-1.922736328

H	3.763359797	0.724355293	-2.076721720
H	4.710323133	3.166963739	-0.728018912
H	3.109612466	2.485068406	-0.394648344
H	4.211678740	2.900592293	0.948182034
H	6.765526571	1.953797375	-0.207752819
H	6.290265353	1.392142671	1.405015380
H	6.785444538	0.224178636	0.159683258
H	-6.239177832	0.711149762	2.927811278
H	-6.074359323	-1.056024064	2.881018768
H	-4.650613363	-0.040190017	3.218020145
H	-2.812039208	1.986452942	2.955272439
O	-1.069145322	-1.223423994	-0.984022899
C	0.083301093	-0.982568505	-1.706632981
O	0.035322356	-1.046764668	-2.925068866

88

PC^{Ga}

C	4.150636	4.178353	0.324381
C	3.532866	3.104124	-0.349182
C	2.600860	3.422537	-1.357789
C	2.297165	4.745473	-1.668054
C	2.926239	5.799088	-1.000326
C	3.858312	5.500961	-0.004796
N	3.844810	1.765812	-0.039740
SI	5.440120	1.263520	0.516123
C	6.743165	1.883492	-0.676224
O	5.395554	-0.390171	0.517333
SI	4.701765	-1.732543	-0.163999
C	5.019246	-3.218698	0.924152
N	2.994385	-1.357524	-0.181414
GA	2.514547	0.452199	-0.157810
O	0.814273	0.831572	0.867215
C	0.137787	0.734363	-0.234502
AU	-1.890686	0.473405	-0.258479
P	-4.154979	-0.090592	-0.291423
C	-5.212598	1.466186	-0.638340
C	-5.044958	1.858276	-2.115742
C	1.990386	-2.341914	-0.316541
C	2.042218	-3.301806	-1.345789
C	1.055805	-4.279892	-1.466781
C	-0.020120	-4.316899	-0.577123
C	-0.085424	-3.368371	0.445935
C	0.908108	-2.400644	0.582079
C	5.463889	-1.963315	-1.864786
C	5.841289	1.746200	2.284382
C	-4.627788	-0.841754	1.406860
C	-5.945716	-1.627415	1.411633
C	-4.406566	-1.390444	-1.677397
C	-3.584788	-0.956586	-2.908425
C	-3.476599	-1.756212	1.870802
C	-4.708385	0.290520	2.444671
C	-3.797927	-2.727085	-1.218778
C	-5.869462	-1.611782	-2.083402
C	-6.705721	1.306343	-0.324764
C	-4.628986	2.635954	0.179655
H	-0.912059	-3.386860	1.157657
H	-0.795789	-5.076421	-0.679119
H	1.122773	-5.010616	-2.274408
H	4.670128	-3.023345	1.947313
H	6.091278	-3.457771	0.958711
H	4.484237	-4.099598	0.542256
H	7.748101	1.594650	-0.337141
H	6.711877	2.978460	-0.761291
H	6.134583	2.799500	2.385801
H	6.679418	1.129343	2.640717
H	4.979040	1.563609	2.940883
H	1.567622	4.952682	-2.452589
H	2.692846	6.833949	-1.250494
H	4.357144	6.306356	0.536793
H	0.857474	-1.674669	1.392991
H	2.857005	-3.260523	-2.069153
H	2.112255	2.617670	-1.907788
H	4.853028	3.975091	1.131626
H	-4.808392	-0.171087	3.438159
H	-3.794201	0.898829	2.450813
H	-5.573517	0.945077	2.298503
H	-3.695138	-2.082875	2.898638
H	-3.357326	-2.649908	1.254769
H	-2.520543	-1.214669	1.879385
H	-6.146179	-1.961290	2.441178
H	-6.798889	-1.021296	1.087442
H	-5.899171	-2.523377	0.782725
H	-3.796138	-3.407747	-2.083093
H	-2.756724	-2.609683	-0.889438
H	-4.375867	-3.209722	-0.424129
H	-5.902052	-2.413841	-2.836533
H	-6.498260	-1.921972	-1.241432
H	-6.316011	-0.720737	-2.538586
H	-3.640994	-1.764854	-3.653021
H	-3.952320	-0.042418	-3.380106
H	-2.528229	-0.808404	-2.645647
H	-5.158565	3.554426	-0.115340
H	-4.746798	2.515331	1.258876
H	-3.560356	2.773665	-0.035886
H	-5.515941	2.842215	-2.257921
H	-3.986379	1.951998	-2.392023
H	-5.532557	1.158553	-2.801954
H	-7.222439	2.234682	-0.612819
H	-7.166620	0.483644	-0.882850
H	-6.893418	1.148425	0.742930
H	6.560078	-2.005476	-1.784488
H	5.125358	-2.892297	-2.344291
H	5.199220	-1.123427	-2.523074
H	6.576968	1.458550	-1.675850
O	0.858149	0.622562	-1.304646

85

Complex I^{In}

C	1.490176	-3.544110	0.265850
C	2.499840	-2.953709	-0.530749
C	3.057069	-3.759536	-1.551318
C	2.633172	-5.071767	-1.753947
C	1.625210	-5.629966	-0.962848
C	1.055708	-4.848517	0.046596
N	2.905613	-1.636186	-0.312903
SI	4.524217	-1.031551	-0.549522
C	4.851879	-0.475797	-2.317029
IN	1.374172	-0.217419	0.093176
AU	-1.222790	-0.275231	0.177243
P	-3.571353	-0.178384	0.181072
C	-4.127644	1.282196	1.289709
C	-3.831250	2.602893	0.559245
N	2.346706	1.695438	0.213378
SI	4.014532	1.804605	0.695720
C	4.905289	3.086788	-0.345630
C	1.477399	2.778906	0.092216
C	1.508720	3.890609	0.966575
C	0.604244	4.942378	0.838350
C	-0.376973	4.928439	-0.157505
C	-0.422458	3.844917	-1.037620
C	0.490550	2.799236	-0.923953
C	4.299189	2.140393	2.525180
C	-4.263849	-1.831681	0.860168
C	-5.750864	-2.073170	0.570453
C	-4.169661	0.099683	-1.618131
C	-3.237571	1.132978	-2.282494
C	-3.423048	-2.983892	0.273115
C	-4.031418	-1.871530	2.379758
C	5.804811	-2.274619	0.023607
O	4.652601	0.296980	0.434530

C	-3.979572	-1.207433	-2.405655
C	-5.628144	0.558945	-1.743097
C	-5.607523	1.248137	1.693890
C	-3.238197	1.294262	2.549070
H	0.272448	-5.264210	0.683250
H	1.292003	-6.654610	-1.128392
H	3.088812	-5.661478	-2.551571
H	5.618409	-2.550636	1.070818
H	6.811583	-1.838367	-0.044869
H	5.786869	-3.192207	-0.578934
H	5.963324	3.163816	-0.057569
H	4.443142	4.076463	-0.220141
H	4.051835	3.174189	2.803393
H	5.356073	1.968333	2.777074
H	3.687057	1.464782	3.139671
H	-1.166741	3.819221	-1.835788
H	-1.086996	5.750417	-0.249590
H	0.659365	5.779455	1.536872
H	1.055484	-2.959058	1.078988
H	3.820685	-3.337005	-2.205796
H	0.470137	1.982235	-1.647832
H	2.239347	3.908169	1.775481
H	-4.271244	-2.886099	2.731020
H	-2.981959	-1.670410	2.632801
H	-4.671381	-1.173508	2.928722
H	-3.725949	-3.915513	0.774716
H	-3.565131	-3.123043	-0.801116
H	-2.350978	-2.826657	0.458969
H	-6.051245	-3.015842	1.053297
H	-6.390761	-1.277730	0.968597
H	-5.955137	-2.178009	-0.500969
H	-4.158781	-0.990370	-3.469126
H	-2.954482	-1.589865	-2.311031
H	-4.682483	-1.991940	-2.107692
H	-5.878663	0.635453	-2.812385
H	-6.331093	-0.147487	-1.287505
H	-5.793402	1.546530	-1.298686
H	-3.499974	1.191931	-3.349560
H	-3.327002	2.135862	-1.859692
H	-2.186322	0.822101	-2.203228
H	-3.477416	2.201030	3.124981
H	-3.393052	0.432884	3.202894
H	-2.174403	1.335307	2.275419
H	-3.997406	3.423503	1.273084
H	-2.785651	2.661931	0.228923
H	-4.491608	2.776057	-0.296600
H	-5.829254	2.155591	2.276404
H	-6.279061	1.238258	0.828083
H	-5.849139	0.387870	2.328104
H	5.847882	-0.017968	-2.405661
H	4.798070	-1.316238	-3.023513
H	4.103317	0.269973	-2.622630
H	4.852221	2.818932	-1.409965

88

RC^{In}

C	0.505522	-2.681510	1.366477
C	1.460799	-2.975428	0.362581
C	1.471755	-4.301321	-0.131873
C	0.582185	-5.262182	0.343247
C	-0.366877	-4.942412	1.318726
C	-0.394446	-3.640758	1.824287
N	2.329071	-1.989174	-0.099802
SI	3.968245	-2.262504	-0.614128
O	4.652931	-0.752954	-0.720047
SI	4.578512	0.704768	0.074423
C	5.936256	1.795317	-0.616750
N	3.000559	1.343854	-0.298552
In	1.417795	-0.073316	-0.434544
AU	-1.172633	0.129064	-0.441294
P	-3.518106	0.098673	-0.259184
C	-3.991813	0.180632	1.596573
C	-5.429264	-0.258153	1.905433
C	2.613449	2.678923	-0.198472
C	1.608448	3.206715	-1.044608
C	1.185341	4.528555	-0.939635
C	1.758638	5.389108	0.000403
C	2.760143	4.893744	0.839212
C	3.173673	3.565345	0.752280
C	4.822249	0.376431	1.912253
C	-4.136307	-1.546853	-1.022669
C	-3.347998	-1.800165	-2.323989
C	-4.262652	1.594419	-1.194022
C	-4.134702	1.341418	-2.705599
C	-3.761453	-2.701165	-0.077265
C	-5.642196	-1.590118	-1.311543
C	4.880501	-3.315605	0.644582
C	4.145805	-2.994527	-2.336966
C	-5.728893	1.895941	-0.857485
C	-3.387323	2.828461	-0.894584
C	-2.998014	-0.694586	2.385724
C	-3.785454	1.617505	2.103152
H	0.404623	4.893658	-1.609382
H	1.431237	6.425877	0.078258
H	3.218920	5.545920	1.584731
H	5.816614	1.900884	-1.704050
H	6.921587	1.350013	-0.418298
H	5.917682	2.799369	-0.172378
H	5.925429	-3.470107	0.340005
H	4.405738	-4.301075	0.751057
H	3.789185	-4.032760	-2.385891
H	5.201006	-2.984864	-2.647423
H	3.570417	-2.402208	-3.062857
H	-1.111432	-3.371919	2.602082
H	-1.064093	-5.696276	1.684524
H	0.624204	-6.274007	-0.064296
H	1.169211	2.554713	-1.802267
H	3.932704	3.194663	1.442188
H	0.502415	-1.683494	1.809819
H	2.178662	-4.568993	-0.917852
H	-4.402344	2.272538	-3.226875
H	-3.104362	1.086021	-2.986809
H	-4.808642	0.557625	-3.066017
H	-3.725465	3.651291	-1.542481
H	-3.455090	3.171109	0.140377
H	-2.330086	2.627592	-1.118285
H	-6.066413	2.733427	-1.487452
H	-6.387888	1.043715	-1.058196
H	-5.865286	2.199476	0.186251
H	-3.892553	1.606181	3.198113
H	-2.780827	1.992640	1.870185
H	-4.526410	2.320476	1.709631
H	-5.612114	-0.116912	2.981827
H	-6.174238	0.334188	1.362197
H	-5.599892	-1.317247	1.683449
H	-3.201613	-0.560549	3.458868
H	-3.080746	-1.759485	2.158429
H	-1.961158	-0.386966	2.190610
H	-3.609482	-2.804943	-2.688935
H	-3.575371	-1.086783	-3.119273
H	-2.264835	-1.777719	-2.137462
H	-3.967065	-3.645186	-0.603582
H	-2.693089	-2.691884	0.176889
H	-4.350120	-2.707516	0.845829
H	-5.894668	-2.591354	-1.693500
H	-6.248170	-1.414988	-0.415807
H	-5.940102	-0.865802	-2.078059
H	5.779880	-0.130712	2.099785
H	4.808064	1.306679	2.497652
H	4.012445	-0.267128	2.285765

H	4.874010	-2.827277	1.628823
C	0.327724	2.307150	2.280504
O	-0.363379	3.211021	2.012460
O	1.001422	1.392620	2.567925

88
TSI^{In}

C	1.530465	2.722264	-1.243429
C	2.755340	2.331797	-0.654324
C	3.704480	3.354340	-0.426142
C	3.442113	4.678271	-0.772904
C	2.219161	5.042997	-1.341118
C	1.263508	4.048888	-1.567794
N	3.010736	0.998668	-0.328627
SI	4.581525	0.229986	-0.357625
O	4.251560	-1.393638	-0.253047
SI	3.254301	-2.578344	-0.854995
C	3.918722	-4.243551	-0.323915
N	1.715718	-2.243722	-0.101991
IN	1.395052	-0.199031	0.300117
AU	-1.087936	0.499686	0.650757
P	-3.225743	0.584331	-0.393291
C	-3.177999	-0.552465	-1.935512
C	-4.337258	-0.321104	-2.913955
C	0.643219	-3.125393	0.030802
C	-0.215312	-3.057551	1.152000
C	-1.289641	-3.931648	1.296910
C	-1.543716	-4.915307	0.337357
C	-0.701971	-5.003023	-0.774303
C	0.364776	-4.120930	-0.932768
C	3.217985	-2.412706	-2.728004
C	-3.591975	2.392358	-0.906582
C	-3.126252	3.316563	0.235937
C	-4.542189	-0.040024	0.849484
C	-4.772753	1.042723	1.916980
C	-2.720749	2.754841	-2.120465
C	-5.063615	2.670251	-1.239743
C	5.461821	0.629222	-1.964838
C	5.664131	0.613854	1.124226
C	-5.886215	-0.421099	0.214668
C	-3.953435	-1.252331	1.598360
C	-1.833138	-0.347614	-2.661153
C	-3.181122	-2.019668	-1.475455
H	-1.927403	-3.850411	2.178555
H	-2.380656	-5.603396	0.455444
H	-0.883577	-5.761197	-1.538026
H	4.011035	-4.282433	0.770082
H	4.910959	-4.417542	-0.763783
H	3.255084	-5.059016	-0.641650
H	6.433566	0.117421	-2.011432
H	5.639919	1.709350	-2.059655
H	6.001333	1.659496	1.130691
H	6.556239	-0.029277	1.117313
H	5.113419	0.428045	2.057152
H	0.303262	4.306962	-2.018176
H	2.014676	6.081063	-1.602681
H	4.201763	5.437983	-0.581355
H	-0.004489	-2.321883	1.929809
H	0.989348	-4.188163	-1.824365
H	0.781928	1.955532	-1.459372
H	4.653322	3.104718	0.049137
H	-5.399356	0.604100	2.707616
H	-3.830472	1.362977	2.381558
H	-5.299368	1.921612	1.531496
H	-4.671691	-1.551913	2.376463
H	-3.767551	-2.117685	0.959116
H	-3.008057	-0.989756	2.093739
H	-6.580116	-0.710504	1.018661
H	-6.342131	0.410942	-0.333624
H	-5.799257	-1.277066	-0.463384
H	-2.985162	-2.646966	-2.357447
H	-2.385760	-2.224704	-0.747460
H	-4.142298	-2.335182	-1.057251
H	-4.258282	-1.062030	-3.724182
H	-5.317335	-0.450398	-2.441945
H	-4.303517	0.671609	-3.376491
H	-1.793804	-1.054350	-3.503488
H	-1.695651	0.658692	-3.062997
H	-0.992244	-0.574266	-1.992015
H	-3.241384	4.358044	-0.100244
H	-3.701047	3.198265	1.157153
H	-2.064597	3.152933	0.469632
H	-2.821749	3.836323	-2.295304
H	-1.659421	2.547510	-1.930663
H	-3.026903	2.243254	-3.038184
H	-5.155373	3.717821	-1.565124
H	-5.436307	2.036416	-2.052085
H	-5.719471	2.541317	-0.371868
H	4.230877	-2.492246	-3.148672
H	2.595306	-3.190227	-3.192602
H	2.804048	-1.434763	-3.014807
H	4.858034	0.308100	-2.824598
C	0.252926	0.760232	2.655095
O	-0.562321	1.493171	3.157676
O	1.301302	0.158889	2.849392

88
INT^{In}

C	0.304986	-4.115542	-0.691616
C	0.599415	-3.080906	0.225179
C	-0.139850	-3.061188	1.429649
C	-1.130683	-4.005000	1.687816
C	-1.404420	-5.022829	0.770490
C	-0.667133	-5.073752	-0.414679
N	1.581633	-2.127901	-0.039014
IN	1.440761	-0.149887	0.666055
SI	2.933076	-2.312330	-1.136230
C	2.476293	-2.136472	-2.950275
O	3.900760	-1.031337	-0.709376
SI	4.184105	0.600726	-0.835948
C	5.728284	0.922901	0.172633
N	2.724308	1.299520	-0.159330
C	2.414392	2.658870	-0.154295
C	1.096404	3.120554	0.047995
C	0.780991	4.474883	0.041315
C	1.771003	5.435508	-0.179822
C	3.083493	5.003309	-0.382556
C	3.404269	3.647852	-0.364132
C	4.409320	1.091698	-2.630750
C	3.866283	-3.896277	-0.804401
AU	-1.239046	0.284509	1.062283
P	-2.965373	0.288644	-0.602747
C	-4.574896	-0.223139	0.303718
C	-4.241785	-1.401350	1.241485
C	-2.589903	-0.939932	-2.020145
C	-2.797006	-2.374459	-1.510093
C	-3.127954	2.069027	-1.291970
C	-4.453880	2.348700	-2.012279
C	-3.443812	-0.724141	-3.277509
C	-1.095542	-0.832439	-2.378080
C	-2.957997	3.060971	-0.123873
C	-1.961059	2.340102	-2.256893
C	-5.023129	0.930205	1.216947
C	-5.735332	-0.605952	-0.624119
H	-1.682233	-3.951614	2.627645
H	-2.174018	-5.765863	0.978551
H	-0.862419	-5.859530	-1.146287
H	4.187436	-3.928862	0.245681
H	4.758369	-3.954081	-1.443989
H	3.246294	-4.781192	-1.000269

H	5.293440	0.594766	-3.055086
H	4.546308	2.176607	-2.733366
H	6.063805	1.966518	0.110409
H	6.546030	0.282567	-0.188837
H	5.547193	0.686056	1.230277
H	-0.252947	4.782203	0.206256
H	1.526233	6.497226	-0.187872
H	3.878744	5.732187	-0.547431
H	0.095954	-2.311672	2.184895
H	0.836959	-4.157575	-1.642305
H	0.303044	2.385312	0.212985
H	4.444196	3.351520	-0.499950
H	-5.834823	0.554554	1.857156
H	-4.213996	1.269728	1.877576
H	-5.414367	1.788189	0.661052
H	-5.147167	-1.641126	1.819078
H	-3.926761	-2.306565	0.718449
H	-3.451638	-1.130677	1.956946
H	-6.617264	-0.824265	-0.002720
H	-6.006736	0.203168	-1.311179
H	-5.522693	-1.504212	-1.213588
H	-2.407469	-3.060403	-2.276067
H	-2.234258	-2.570286	-0.589654
H	-3.850732	-2.623784	-1.351026
H	-3.203828	-1.523060	-3.995611
H	-4.518286	-0.777504	-3.070073
H	-3.232351	0.230351	-3.771723
H	-0.888016	-1.555501	-3.180990
H	-0.794986	0.156126	-2.733577
H	-0.466393	-1.107260	-1.522570
H	-2.906014	4.076227	-0.544788
H	-3.783870	3.039239	0.590145
H	-2.024861	2.877280	0.426360
H	-1.984989	3.407761	-2.520511
H	-0.987645	2.143579	-1.789675
H	-2.033985	1.769873	-3.188100
H	-4.421659	3.373993	-2.411559
H	-4.626754	1.670684	-2.854829
H	-5.314768	2.289879	-1.337473
H	3.382014	-2.113529	-3.573288
H	1.850181	-2.970692	-3.295452
H	1.917727	-1.205563	-3.121085
H	3.531109	0.803096	-3.223924
O	1.220123	0.124720	2.810730
C	-0.065986	0.364328	2.812730
O	-0.737111	0.633553	3.801894

88

TSII^{In}

C	-4.959420090	-3.191102606	0.863193708
C	-4.080298166	-2.492582780	0.006234975
C	-3.486068091	-3.224821159	-1.041597980
C	-3.758424005	-4.577526906	-1.223666416
C	-4.642333612	-5.251181203	-0.376421683
C	-5.239118235	-4.542327226	0.666899637
N	-3.829403575	-1.124113319	0.190415606
IN	-2.172978800	-0.170356551	-0.629679704
SI	-4.893068379	-0.023591757	1.057808606
C	-4.852873569	-0.246921874	2.921688341
O	-4.248756477	1.462350660	0.702328849
SI	-2.996671811	2.543345899	0.811912492
C	-3.592123783	4.201005824	0.191523180
N	-1.798374086	1.839742771	-0.262196228
C	-0.728424056	2.548257288	-0.824266449
C	-0.159543207	3.653014124	-0.154513414
C	0.859376558	4.402356328	-0.738399160
C	1.368774016	4.057722525	-1.991668895
C	0.837575427	2.947600607	-2.652262949
C	-0.199628925	2.213157170	-2.084826216
C	-2.427120235	2.618238519	2.598679207
C	-6.636149408	-0.085722450	0.389331644
AU	2.025452305	-0.788253782	-0.830519531
P	3.747859579	-0.215263150	0.650238526
C	4.794866865	-1.760625376	1.080255528
C	3.839110123	-2.958972780	1.248595501
C	2.924321297	0.475518175	2.239975695
C	2.305113848	-0.695188723	3.022108428
C	4.856149130	1.126859653	-0.147267597
C	6.200530003	1.357430249	0.554412255
C	3.869413392	1.256213884	3.162488051
C	1.743169774	1.375385368	1.826743636
C	5.092999138	0.737204242	-1.620570082
C	4.069050610	2.446272578	-0.185953399
C	5.685810017	-2.100521358	-0.126217190
C	5.674169482	-1.616890224	2.329350177
H	-3.275363569	-5.108993858	-2.044952209
H	-4.858139569	-6.309086578	-0.525209303
H	-5.926147769	-5.045777238	1.349018640
H	-6.644811144	0.196128608	-0.672414349
H	-7.285923346	0.608824927	0.940317257
H	-7.060133793	-1.094930151	0.478564153
H	-3.264844075	2.886052592	3.258446278
H	-1.633235948	3.364236107	2.741653213
H	-2.781923873	4.940804838	0.161215418
H	-4.385121898	4.585896745	0.848261607
H	-4.000880762	4.097632494	-0.822965947
H	1.268762331	5.256252681	-0.196282766
H	2.173903750	4.638302441	-2.441405763
H	1.225044064	2.648963589	-3.627013007
H	-2.789313602	-2.726187069	-1.718168829
H	-5.421689967	-2.674402807	1.703826255
H	-0.514579188	3.920003601	0.841040833
H	-0.610596877	1.364728503	-2.634778894
H	6.148748038	-3.080737775	0.062005554
H	5.100110796	-2.180365215	-1.051579763
H	6.495072319	-1.378904013	-0.276603808
H	4.449131723	-3.868459244	1.358801237
H	3.194317945	-2.882495143	2.127008191
H	3.199359889	-3.079629146	0.363416370
H	6.269867737	-2.535833625	2.443566222
H	6.372803303	-0.776000291	2.256354646
H	5.083380839	-1.497183758	3.244252025
H	1.689539465	-0.272189078	3.830204208
H	1.648795296	-1.302414867	2.384417517
H	3.053402627	-1.345081372	3.486757224
H	3.311417419	1.549492588	4.065334368
H	4.731422663	0.661316860	3.484181782
H	4.238628729	2.176202710	2.696088729
H	1.203922718	1.671617996	2.739984538
H	2.041531245	2.286458589	1.304968034
H	1.041286332	0.835498491	1.176094200
H	5.611932939	1.573314351	-2.113532859
H	5.709801210	-0.156194861	-1.742668292
H	4.139185970	0.571302972	-2.140327873
H	4.634548677	3.158881460	-0.804994473
H	3.082657375	2.321287552	-0.649352606
H	3.943823838	2.899966275	0.802636573
H	6.720830467	2.182835493	0.044304304
H	6.081722728	1.640489497	1.606304050
H	6.855708505	0.480577484	0.504250823
H	-5.305105385	0.630078586	3.407443401
H	-5.411329166	-1.134462840	3.248084407
H	-3.820057512	-0.346431297	3.283451369
H	-2.033286463	1.643946521	2.921347969
O	-0.714342872	-1.437175513	-1.262516681
C	0.370032769	-1.100645395	-2.018738383
O	0.266854779	-0.926893446	-3.225960818

88

PC^{In}

C	4.635249	4.354795	-0.135490
C	3.672214	3.340569	-0.345118
C	2.448986	3.736000	-0.929189
C	2.202363	5.060101	-1.278943
C	3.169087	6.047585	-1.071079
C	4.386895	5.677029	-0.497810
N	3.925353	2.010523	-0.001154
SI	5.509309	1.329600	0.343185
C	6.681572	1.580337	-1.096655
O	5.196878	-0.283903	0.560911
SI	4.604671	-1.698390	-0.082831
C	5.090461	-3.126746	1.019592
N	2.881134	-1.435409	-0.036538
IN	2.384261	0.595461	0.037767
O	0.428277	0.953451	1.050432
C	-0.207024	0.846365	-0.069242
AU	-2.229189	0.506863	-0.145023
P	-4.466870	-0.137606	-0.287551
C	-5.561994	1.402097	-0.596028
C	-5.353438	1.872363	-2.045069
C	1.895063	-2.420695	-0.178525
C	2.050799	-3.499181	-1.076223
C	1.069687	-4.481588	-1.201908
C	-0.106870	-4.415355	-0.451728
C	-0.276325	-3.353172	0.439623
C	0.708566	-2.379528	0.583579
C	5.319680	-1.895287	-1.809725
C	6.259523	1.918494	1.956183
C	-4.973534	-0.989333	1.351576
C	-6.264693	-1.814131	1.269627
C	-4.627859	-1.371394	-1.745584
C	-3.779680	-0.848886	-2.922933
C	-3.810025	-1.890704	1.810156
C	-5.126145	0.086594	2.439924
C	-3.992141	-2.711205	-1.335176
C	-6.068772	-1.617357	-2.212825
C	-7.059887	1.182146	-0.347632
C	-5.045586	2.547236	0.298577
H	-1.181450	-3.285548	1.045187
H	-0.877238	-5.179192	-0.559880
H	1.222841	-5.300829	-1.906503
H	4.788575	-2.925781	2.056490
H	6.176793	-3.291218	0.995875
H	4.596731	-4.052854	0.693144
H	7.648246	1.098915	-0.890433
H	6.864297	2.647398	-1.282508
H	6.588635	2.965135	1.916475
H	7.134094	1.297916	2.200395
H	5.530400	1.820587	2.772581
H	1.242869	5.319569	-1.729152
H	2.976454	7.083266	-1.350045
H	5.158580	6.427954	-0.320383
H	0.569034	-1.570100	1.300485
H	2.945177	-3.550049	-1.698223
H	1.679064	2.986422	-1.128096
H	5.589534	4.107311	0.328465
H	-5.246221	-0.426322	3.405604
H	-4.232735	0.721208	2.508676
H	-6.005961	0.721361	2.294019
H	-4.050459	-2.273920	2.813287
H	-3.644190	-2.749761	1.156467
H	-2.872373	-1.321513	1.876953
H	-6.490774	-2.205496	2.273332
H	-7.125101	-1.218974	0.944190
H	-6.166570	-2.675486	0.599757
H	-3.939778	-3.345409	-2.232594
H	-2.966695	-2.579494	-0.964293
H	-4.580350	-3.252298	-0.587076
H	-6.050469	-2.379460	-3.006824
H	-6.714098	-1.990890	-1.410119
H	-6.529730	-0.718293	-2.636942
H	-3.782687	-1.618753	-3.709289
H	-4.161445	0.075940	-3.361000
H	-2.738431	-0.680462	-2.615176
H	-5.591949	3.463181	0.027067
H	-5.199328	2.371296	1.365606
H	-3.974439	2.726254	0.130742
H	-5.848638	2.848314	-2.156759
H	-4.288991	2.010076	-2.276708
H	-5.794839	1.194287	-2.782313
H	-7.594810	2.107976	-0.609196
H	-7.473602	0.375370	-0.962867
H	-7.281794	0.965093	0.703025
H	6.416465	-1.819285	-1.781554
H	5.061175	-2.870312	-2.246047
H	4.937156	-1.113601	-2.481249
H	6.265008	1.142697	-2.013919
O	0.493635	0.775007	-1.146643

44
[Al(^{Si}NON')]·

Al	-0.075371	0.256554	-2.439142
H	-1.656665	-2.406227	-5.641722
C	2.671017	1.899974	-1.952661
C	2.635449	0.859257	-2.901175
C	3.851178	0.226438	-3.224487
C	5.048198	0.626387	-2.632081
C	5.066055	1.653547	-1.685731
C	3.866306	2.282752	-1.345891
N	1.412446	0.469037	-3.492761
Si	1.284485	0.150203	-5.222691
C	1.674500	-1.632064	-5.663779
H	1.082822	-2.319222	-5.041866
H	-4.270424	0.283433	-3.355111
H	-2.706447	2.318047	-4.929499
N	-1.615121	-0.245404	-3.311388
Si	-1.816710	-0.000554	-5.046675
C	-2.379038	-1.601907	-5.837440
C	-2.628990	-0.866138	-2.543072
C	-3.985613	-0.509281	-2.664054
C	-4.962897	-1.135442	-1.891307
C	-4.615685	-2.118566	-0.961766
C	-3.273235	-2.477125	-0.823297
C	-2.296766	-1.869679	-1.611455
C	-2.948334	1.419026	-5.513297
H	-6.006345	-0.838987	-2.007839
H	-2.813503	1.655785	-6.578997
H	3.850980	-0.600718	-3.934972
H	1.431478	-1.827544	-6.718278
H	-4.008263	1.178382	-5.355860
H	2.736469	-1.869693	-5.511577
C	2.327533	1.358059	-6.194804
O	-0.309933	0.433296	-5.584214
H	-5.381815	-2.599905	-0.354026
H	-2.982569	-3.247619	-0.107943
H	-1.254408	-2.188539	-1.528484
H	1.742801	2.422629	-1.709509
H	3.860362	3.090885	-0.613101
H	6.002467	1.959675	-1.219461
H	5.974776	0.119198	-2.904979
H	2.050553	2.390564	-5.941279
H	2.166482	1.209452	-7.271990
H	3.397605	1.228623	-5.986484
H	-2.479280	-1.480686	-6.925343
H	-3.353831	-1.913408	-5.436798

47
[CO_2Al(^{Si}NON')]·

C	-3.733658717	0.727677017	-0.963106350
C	-2.766561021	0.614890285	0.049381292

C	-3.006019008	1.252415009	1.277751238
C	-4.176943716	1.979813992	1.487147273
C	-5.137634511	2.076578120	0.477944994
C	-4.909825675	1.446234498	-0.746790216
N	-1.568406124	-0.130925811	-0.156352443
SI	-1.649208438	-1.879671547	-0.322734913
C	-2.514115220	-2.617921100	1.162630346
O	-0.055688646	-2.340291858	-0.396183931
SI	1.419386602	-1.978493566	0.280036129
C	2.763939482	-2.861102537	-0.666240884
N	1.565897802	-0.236705088	0.052494782
AL	0.039095031	0.673122853	-0.254802202
O	0.102787778	2.046219730	-1.697897693
C	0.107228627	2.858965164	-0.736413554
C	2.841443076	0.386153663	0.102965983
C	3.686506474	0.223363332	1.215073311
C	4.948870330	0.814646390	1.246992189
C	5.391654369	1.598422705	0.179809149
C	4.556324341	1.777188032	-0.924752613
C	3.301373021	1.171380697	-0.968695635
C	1.380230222	-2.500425750	2.080975561
C	-2.457359650	-2.448238211	-1.910998421
H	4.888584310	2.382626434	-1.769191385
H	6.376090476	2.065460460	0.209103944
H	5.586361089	0.670140777	2.120378489
H	2.726725614	-2.588470130	-1.729703966
H	2.645073597	-3.950231976	-0.579582616
H	3.755871495	-2.592950974	-0.277278712
H	-2.566079981	-3.712699395	1.080800074
H	-3.541516866	-2.232772792	1.234533279
H	-3.533012386	-2.225309321	-1.915592612
H	-2.337285957	-3.535669410	-2.023101644
H	-1.996626686	-1.958863068	-2.780194193
H	-4.341834138	2.469314775	2.447849994
H	-6.054555164	2.642366778	0.643843263
H	-5.647973688	1.522038771	-1.546440729
H	2.668989712	1.288011969	-1.849749620
H	3.336755850	-0.360005493	2.067017794
H	-2.260893326	1.164423437	2.069955582
H	-3.545077435	0.256616313	-1.927794624
H	1.075246542	-3.553942982	2.161300315
H	2.365185904	-2.398610436	2.556738850
H	0.662873887	-1.890296189	2.647840534
H	-1.989409557	-2.361274107	2.092968949
O	0.075708260	2.492788127	0.471682886

44

[Al(^{Si}NON’)]$^-$ (gas phase)

C	-2.663163	1.677309	-2.061027
C	-1.660292	2.277636	-2.863647
C	-1.597176	3.692273	-2.836800
C	-2.495459	4.452656	-2.092828
C	-3.482408	3.836744	-1.315637
C	-3.546174	2.441246	-1.300999
N	-0.777885	1.480734	-3.591054
Si	-0.277534	1.973380	-5.179743
O	0.075300	0.591220	-6.032662
Si	-0.208263	-0.989920	-5.589810
C	0.669861	-2.023170	-6.901614
N	0.437149	-1.228663	-3.996053
C	1.072434	-2.426454	-3.665502
C	2.284091	-2.422139	-2.932716
C	2.923255	-3.607340	-2.575337
C	2.392585	-4.847568	-2.940279
C	1.190352	-4.876797	-3.654213
C	0.537939	-3.694756	-3.995652
Al	0.101886	0.034261	-2.514366
C	-2.073226	-1.279032	-5.690455
C	-1.647399	2.869897	-6.121604
C	1.299804	3.011413	-5.225480
H	-2.564407	2.263793	-6.107528
H	-2.576116	-0.636034	-4.953936
H	1.169809	3.977827	-4.717546
H	-4.182922	4.433577	-0.729837
H	2.108037	2.468254	-4.714919
H	-2.740282	0.588939	-2.065540
H	-2.414206	5.542101	-2.107354
H	-0.420172	-3.738082	-4.516958
H	-2.463826	-1.034399	-6.689832
H	-2.331716	-2.323479	-5.462693
H	-0.808657	4.194043	-3.400175
H	2.713778	-1.457396	-2.660866
H	-4.303893	1.936897	-0.697041
H	1.614737	3.204388	-6.262164
H	3.857656	-3.560242	-2.011290
H	2.901804	-5.773571	-2.669211
H	0.746759	-5.834418	-3.936839
H	1.757948	-1.896774	-6.811771
H	0.363219	-1.678145	-7.900314
H	0.446399	-3.094239	-6.817960
H	-1.341933	3.007927	-7.169693
H	-1.884986	3.852435	-5.694108

44

[Ga(^{Si}NON’)]$^-$

Ga	-0.036425	-0.232754	2.459202
H	-3.355682	2.803859	0.013753
C	3.938210	0.355104	3.150850
C	2.797571	-0.423661	2.869722
C	2.937329	-1.477773	1.944840
C	4.158364	-1.737566	1.327503
C	5.284995	-0.967557	1.629244
C	5.164177	0.076537	2.548335
N	1.562925	-0.164132	3.487940
H	-1.914790	2.330172	5.755721
H	0.698912	2.643595	4.788609
H	-5.708020	2.196028	0.593403
Si	1.387320	0.292263	5.180967
C	1.441820	2.150453	5.431971
O	-0.122286	-0.251634	5.603249
Si	-1.704058	-0.065693	5.145780
C	-2.557401	-1.663312	5.624659
N	-1.629667	0.239145	3.409410
C	-2.713122	0.738407	2.668820
C	-2.497154	1.620214	1.589088
C	-3.558843	2.127673	0.845313
C	-4.876196	1.791265	1.169560
C	-5.109164	0.927217	2.241164
C	-4.047036	0.396869	2.971977
C	-2.470898	1.414698	5.999416
C	2.641125	-0.605825	6.236787
H	2.429078	2.564873	5.184532
H	-3.640631	-1.633270	5.446379
H	3.854620	1.197421	3.838463
H	-4.250623	-0.304756	3.780629
H	-2.139428	-2.506458	5.057058
H	1.216683	2.407596	6.477088
H	-1.473751	1.920776	1.350059
H	2.069439	-2.104128	1.726889
H	-2.397811	-1.858768	6.695231
H	-6.130002	0.646993	2.504806
H	4.233173	-2.558961	0.613437
H	6.242298	-1.177361	1.152393
H	6.030093	0.694642	2.790086
H	2.583756	-1.688321	6.059064
H	2.429975	-0.416418	7.299019
H	3.666349	-0.275833	6.026225
H	-2.448549	1.273467	7.089371
H	-3.515827	1.557247	5.693022

47

[CO$_2$Ga(^{Si}NON’)]$^{\cdot}$

C	3.626153	4.106509	0.680427
C	3.480509	3.105792	-0.294168
C	3.011315	3.471007	-1.566340
C	2.698036	4.797571	-1.854561
C	2.861291	5.788278	-0.884465
C	3.327942	5.435576	0.382363
N	3.815366	1.755862	-0.000029
Si	5.460891	1.256189	0.365246
C	6.580574	1.708666	-1.064819
O	5.337354	-0.386577	0.544877
Si	4.685542	-1.768957	-0.097060
C	4.950342	-3.191502	1.079661
N	2.966311	-1.396560	-0.214610
Ga	2.566653	0.405918	-0.131299
O	0.751588	0.977548	0.980982
C	0.282985	1.117612	-0.161934
C	1.966832	-2.387828	-0.361418
C	2.116243	-3.422725	-1.301401
C	1.154061	-4.424696	-1.418246
C	0.010039	-4.404685	-0.620167
C	-0.155331	-3.371818	0.304556
C	0.814526	-2.381551	0.442669
C	5.497555	-2.078780	-1.760689
C	6.122393	1.942819	1.973492
H	-1.043171	-3.341363	0.937325
H	-0.746748	-5.182420	-0.720570
H	1.294325	-5.217975	-2.153675
H	4.578226	-2.933247	2.080040
H	6.018731	-3.436673	1.157194
H	4.416157	-4.087939	0.736551
H	7.621031	1.417578	-0.863941
H	6.557561	2.794264	-1.237261
H	6.265633	3.030313	1.916681
H	7.096962	1.485744	2.198448
H	5.440022	1.722823	2.805479
H	2.329140	5.058894	-2.847017
H	2.619368	6.826366	-1.112382
H	3.447122	6.198385	1.152771
H	0.690870	-1.596612	1.189543
H	2.985777	-3.425447	-1.958386
H	2.893554	2.699128	-2.327806
H	3.957648	3.826260	1.679818
H	6.591916	-2.051428	-1.656249
H	5.226495	-3.059888	-2.173896
H	5.203916	-1.311032	-2.490119
H	6.253791	1.214271	-1.989796
O	0.883052	0.912312	-1.247101

44

[Ga(^{Si}NON’)]$^{-}$ (gas phase)

C	-2.603998	1.645828	-2.002723
C	-1.699114	2.290044	-2.889322
C	-1.745765	3.710013	-2.900556
C	-2.643760	4.422639	-2.111637
C	-3.525541	3.761601	-1.249326
C	-3.485289	2.364984	-1.200529
N	-0.826839	1.541052	-3.654001
Si	-0.312546	2.008318	-5.233241
O	0.134190	0.621042	-6.032176
Si	-0.164041	-0.968345	-5.629572
C	0.677348	-1.975241	-6.987344
N	0.508651	-1.265871	-4.067920
C	1.058099	-2.478142	-3.691227
C	2.211894	-2.523869	-2.865113
C	2.773097	-3.730092	-2.456009
C	2.224139	-4.952490	-2.856458
C	1.083063	-4.935934	-3.665201
C	0.505580	-3.732673	-4.060899
Ga	0.192391	0.071254	-2.496242
C	-2.035007	-1.233994	-5.709862
C	-1.703329	2.810045	-6.231697
C	1.211065	3.126089	-5.282074
H	-2.569374	2.133439	-6.262274
H	-2.517471	-0.603602	-4.949128
H	1.019063	4.097839	-4.804345
H	-4.225679	4.322963	-0.629243
H	2.037846	2.642237	-4.742307
H	-2.609534	0.554632	-1.981673
H	-2.646316	5.514464	-2.159685
H	-0.407002	-3.744821	-4.659833
H	-2.440109	-0.960558	-6.695960
H	-2.302150	-2.281111	-5.504356
H	-1.042540	4.252789	-3.534773
H	2.663563	-1.576854	-2.566766
H	-4.163121	1.824034	-0.535936
H	1.536399	3.307864	-6.317572
H	3.663595	-3.715828	-1.822869
H	2.673064	-5.896242	-2.543784
H	0.626454	-5.877060	-3.981674
H	1.763914	-1.812574	-6.952669
H	0.311528	-1.648522	-7.972072
H	0.493930	-3.052809	-6.886970
H	-1.370820	2.989825	-7.264982
H	-2.035418	3.763773	-5.800683

44

[In(^{Si}NON’)]$^{\cdot}$

In	0.663366	0.345973	-2.889443
H	-2.969081	0.651732	-5.716960
C	0.444531	-3.014322	-2.229744
C	0.637842	-2.739306	-3.606381
C	0.986509	-3.838989	-4.431222
C	1.104020	-5.125737	-3.912123
C	0.910581	-5.369979	-2.550572
C	0.584907	-4.296177	-1.713694
N	0.456866	-1.465398	-4.104082
Si	0.409640	-0.952678	-5.773429
C	-1.054686	-1.709657	-6.663607
H	-1.977765	-1.580221	-6.083704
H	-0.740350	4.366080	-5.529874
H	-4.017577	4.276276	0.034010
N	-1.001842	1.650548	-3.467094
Si	-0.873844	1.887986	-5.192601
C	-2.525846	1.606175	-6.029058
C	-1.760303	2.362816	-2.561283
C	-2.419553	3.571642	-2.899873
C	-3.229781	4.236020	-1.982887
C	-3.393218	3.746455	-0.684924
C	-2.734839	2.565411	-0.321867
C	-1.943723	1.886339	-1.239323
C	-0.096472	3.501721	-5.741368
H	0.862781	3.658297	-5.228412
H	-1.449356	0.956931	-0.942351
H	-3.730667	5.157052	-2.285285
H	1.159422	-3.677658	-5.495507
H	-1.190392	-1.245537	-7.650789
H	-0.897619	-2.787025	-6.813140
C	1.995599	-1.185396	-6.742069
O	0.233619	0.702508	-5.602454
H	-2.295359	3.987772	-3.900008
H	0.178216	-2.190926	-1.560551
H	-2.845978	2.167130	0.687738
H	0.092490	3.478210	-6.824582
H	0.433290	-4.462619	-0.646144
H	1.016417	-6.376144	-2.145788
H	1.361899	-5.947279	-4.582371
H	2.841235	-0.733727	-6.204784
H	1.910585	-0.695917	-7.723292

H	2.228189	-2.245181	-6.911904
H	-2.409575	1.600407	-7.122111
H	-3.229382	2.408386	-5.765798

47
[CO2In(SiNON')]·

C	4.287481975	0.195654711	-0.151367099
C	3.031547489	-0.355418364	0.183764562
C	3.026714703	-1.602987882	0.839666642
C	4.211900161	-2.264555962	1.145591683
C	5.447982869	-1.697718954	0.823564871
C	5.471711730	-0.461714260	0.175858767
N	1.846203847	0.327200420	-0.103052057
SI	1.675366388	2.064841046	-0.337374700
C	2.304101336	2.999967522	1.154411130
O	0.024411381	2.199133068	-0.493362791
SI	-1.512255301	2.130545316	0.143664139
C	-2.686724576	3.070077782	-0.959979277
N	-1.818986535	0.402269830	0.069585392
IN	-0.020333196	-0.591292866	-0.237214583
O	0.031768941	-2.525398849	-1.719843083
C	-0.090450256	-3.215825124	-0.703467884
C	-3.066063120	-0.222558846	0.181002337
C	-4.082772727	0.331267290	0.987142146
C	-5.339018235	-0.266849300	1.068879364
C	-5.614700689	-1.444643852	0.372743096
C	-4.612281085	-2.011286479	-0.419230035
C	-3.363172061	-1.405935610	-0.522378449
C	-1.472119101	2.829599165	1.881149727
C	2.394027598	2.727754112	-1.931497830
H	-4.807613969	-2.929905245	-0.974066153
H	-6.594138147	-1.916282762	0.447879634
H	-6.104841588	0.188616318	1.698426625
H	-2.598025468	2.722287403	-1.997975199
H	-2.465917046	4.146269974	-0.930130887
H	-3.727002746	2.925968471	-0.637463797
H	2.148935982	4.080509167	1.025328706
H	3.379672236	2.824998443	1.295968150
H	3.490574904	2.779807276	-1.910764823
H	2.013872701	3.745386171	-2.103817611
H	2.094847948	2.100480705	-2.782415068
H	4.166893843	-3.230413201	1.650338707
H	6.375739957	-2.213511357	1.069616101
H	6.424233834	-0.003042414	-0.093517278
H	-2.603884921	-1.840477509	-1.175983991
H	-3.878269422	1.232017250	1.566608356
H	2.074354108	-2.055488293	1.125280766
H	4.335777877	1.145541522	-0.683186028
H	-1.059428592	3.848894081	1.870526632
H	-2.478362416	2.881001626	2.319308543
H	-0.848040926	2.210514600	2.540646325
H	1.784300404	2.677975216	2.066589591
O	-0.199131807	-2.915560574	0.489143435

44
[In(SiNON')]- (gas phase)

C	-2.644303	1.722430	-1.940772
C	-1.829680	2.361547	-2.917486
C	-2.000504	3.769943	-3.033985
C	-2.915384	4.468892	-2.252863
C	-3.699249	3.812396	-1.297487
C	-3.544488	2.430279	-1.150512
N	-0.936431	1.629308	-3.664507
Si	-0.444171	2.024698	-5.264302
O	0.226390	0.655042	-5.923835
Si	-0.041787	-0.970530	-5.671251
C	0.865999	-1.848706	-7.075711
N	0.598888	-1.377842	-4.126216
C	1.012743	-2.640735	-3.763135
C	2.089949	-2.820199	-2.850624
C	2.519188	-4.082419	-2.454542
C	1.912732	-5.240908	-2.953042
C	0.851747	-5.096684	-3.852551
C	0.405422	-3.836148	-4.239560
In	0.356147	0.143494	-2.380604
C	-1.905950	-1.263736	-5.840753
C	-1.904916	2.555531	-6.343106
C	0.920289	3.329514	-5.402975
H	-2.652191	1.750366	-6.378869
H	-2.430148	-0.690892	-5.061735
H	0.580014	4.318640	-5.064012
H	-4.412968	4.364191	-0.684617
H	1.777516	3.037141	-4.778996
H	-2.568310	0.637530	-1.841753
H	-3.010635	5.549816	-2.384029
H	-0.446594	-3.755508	-4.916732
H	-2.280018	-0.936090	-6.822506
H	-2.163464	-2.324973	-5.710499
H	-1.379335	4.315445	-3.746496
H	2.592965	-1.928077	-2.473401
H	-4.147042	1.890215	-0.416237
H	1.269994	3.421753	-6.442308
H	3.352787	-4.164606	-1.752616
H	2.257707	-6.229639	-2.648029
H	0.353175	-5.983444	-4.252369
H	1.933308	-1.587200	-7.044011
H	0.462374	-1.532936	-8.049051
H	0.783679	-2.941156	-7.000447
H	-1.571598	2.762757	-7.370933
H	-2.398186	3.454872	-5.949354

97
TSI' [PtBu3AuAl(NON')]

C	0.248507	-3.519699	1.151121
C	1.084599	-2.702121	1.929095
C	1.070532	-2.851789	3.321496
C	0.228224	-3.789768	3.923336
C	-0.602017	-4.596057	3.143397
C	-0.585328	-4.459661	1.752481
N	1.876424	-1.700166	1.305269
AL	1.180717	0.068093	0.990613
AU	-1.135340	0.044877	0.226411
P	-3.319546	-0.003799	-0.870809
C	-4.485079	1.287470	-0.058794
C	-4.926794	0.757376	1.315694
O	2.667485	0.021653	-0.389397
C	3.422801	-1.161225	-0.397554
C	4.556513	-1.252145	-1.179520
C	4.935909	-0.036029	-2.037708
C	4.567945	1.230844	-1.250862
C	3.434699	1.194339	-0.464065
C	5.231473	-2.484028	-1.124177
C	4.733504	-3.509173	-0.310968
C	3.605060	-3.348719	0.499792
C	2.926507	-2.114224	0.502842
C	2.950165	2.199903	0.384470
C	3.641581	3.425250	0.313968
C	4.769586	3.529878	-0.506850
C	5.255266	2.457259	-1.264334
C	6.419261	-0.053687	-2.412565
C	4.081832	-0.070196	-3.331349
N	1.896844	1.839877	1.207530
C	1.132611	2.881236	1.801416
C	0.267685	3.654014	1.010021
C	-0.535091	4.635833	1.586873
C	-0.490632	4.858484	2.965752
C	0.367719	4.095056	3.758880
C	1.177821	3.114964	3.181254
C	-3.082059	0.420976	-2.726729
C	-2.780345	1.924287	-2.848393

C	-4.074836	-1.757955	-0.698688
C	-3.300933	-2.716698	-1.619156
C	-1.809848	-0.300390	-3.217547
C	-4.262786	0.068129	-3.639933
C	-3.800544	-2.247322	0.738189
C	-5.573117	-1.859336	-1.011872
C	-3.653110	2.554432	0.228334
C	-5.729591	1.652356	-0.877936
H	3.268508	-4.155848	1.149611
H	6.135114	-2.647452	-1.708347
H	6.159273	2.579061	-1.858020
H	3.315500	4.269622	0.920394
H	6.665737	0.809543	-3.043269
H	6.658062	-0.955265	-2.990448
H	7.058718	-0.029887	-1.520806
H	4.310836	0.803909	-3.956123
H	3.010121	-0.056883	-3.095333
H	4.300452	-0.983323	-3.901677
H	5.258402	-4.465412	-0.290221
H	5.303814	4.480636	-0.539329
H	-3.611828	-3.744084	-1.376368
H	-3.510050	-2.552845	-2.681273
H	-2.217661	-2.647559	-1.451524
H	-4.115750	-3.300040	0.807228
H	-2.728208	-2.199175	0.973993
H	-4.343785	-1.686093	1.501643
H	-5.880239	-2.913664	-0.926436
H	-6.185542	-1.285007	-0.307944
H	-5.811443	-1.525111	-2.028005
H	-1.581195	0.059005	-4.232627
H	-0.952405	-0.068703	-2.568971
H	-1.917013	-1.386693	-3.265067
H	-2.463813	2.123939	-3.883243
H	-3.652268	2.553927	-2.644392
H	-1.958936	2.224595	-2.183920
H	-4.029740	0.402810	-4.663038
H	-4.444942	-1.011463	-3.685421
H	-5.191719	0.561598	-3.332734
H	-4.267257	3.238831	0.833499
H	-2.746217	2.314597	0.800745
H	-3.351439	3.090088	-0.675111
H	-6.339195	2.359710	-0.293804
H	-5.478110	2.145081	-1.823778
H	-6.354842	0.779789	-1.099087
H	-5.444283	1.574601	1.840612
H	-5.627326	-0.081019	1.245139
H	-4.068159	0.458806	1.929810
H	0.225763	3.458994	-0.062191
H	-1.209512	5.220465	0.959427
H	-1.124100	5.621334	3.419389
H	0.409761	4.262622	4.835941
H	1.848901	2.514152	3.794864
H	1.720817	-2.219076	3.925267
H	0.224328	-3.891325	5.009429
H	-1.260797	-5.325256	3.615894
H	-1.237011	-5.078728	1.134005
H	0.253952	-3.391939	0.067976
O	0.463139	0.137891	3.189193
C	-0.722330	0.117447	3.013236
O	-1.872183	0.108173	3.257532
